# Global availability of data on HPV genotype-distribution in cervical, vulvar and vaginal disease and genotype-specific prevalence and incidence of HPV infection in females

**DOI:** 10.1186/s13027-015-0008-y

**Published:** 2015-04-28

**Authors:** Monika Wagner, Liga Bennetts, Harshila Patel, Sharon Welner, Silvia de Sanjose, Thomas W Weiss

**Affiliations:** LASER Analytica, Montréal, Québec Canada; Merck Center for Observational and Real-World Evidence, West Point, PA USA; Cancer Epidemiology Research Program, Institut Català d’Oncologia-Catalan Institute of Oncology, IDIBELL, L’Hospitalet de Llobregat, Barcelona, Spain; CIBER en Epidemiología y Salud Pública, Barcelona, Spain

**Keywords:** HPV, Genotype, Geographic region, Epidemiology, Genital infection, Cervical lesions, Cervical cancer

## Abstract

**Background:**

Country-level HPV genotyping data may be sought by decision-makers to gauge the genotype-specific burden of HPV-related diseases in their jurisdiction and assess the potential impact of HPV vaccines. We investigated, by country, the availability of published literature on HPV genotypes in cervical, vaginal and vulvar cancers and intraepithelial neoplasms (CINs, VaINs and VINs) and on prevalence and incidence of genital HPV infections among women without clinically manifest disease.

**Findings:**

Primary sources of publications were the PubMed/Medline and EMBASE databases. Original studies or meta-analyses published from 2000, covering genotypes 16 and 18 and at least one of genotypes 31/33/45/52/58, were included. Key exclusion criteria were language not English, cervical lesions not histologically confirmed (cytology only), special populations (e.g., immunocompromised) and, for cervical studies, small population (<50). A total of 727 studies reporting HPV genotype-specific data were identified: 366 for cervical cancers and CINs, 43 for vulvar or vaginal cancers and VINs/VaINs, and 395 and 21 for infection prevalence and incidence, respectively, in general female population samples. A large proportion of studies originated from a small set of countries. Cervical cancer/CIN typing data was scarce for several regions with the highest cervical cancer burden, including Eastern, Middle and Western Africa, Central America, South-East Asia, South Asia, and Eastern Europe. Data for vulvar/vaginal disease was limited outside of Europe and North America.

**Conclusions:**

Although a large body of published HPV genotype-specific data is currently available, data gaps exist for genotype-specific infection incidence and several world regions with the highest cervical cancer burden.

**Electronic supplementary material:**

The online version of this article (doi:10.1186/s13027-015-0008-y) contains supplementary material, which is available to authorized users.

## Findings

### Background

Human papillomaviruses (HPV) belong to the most prevalent sexually transmitted infections worldwide [1], with genotypes 16, 18, 52, 58, 31, 51, and 56 among the most frequently detected in women with normal cervical cytology [2-4]. Globally, approximately 70% of cervical cancers are attributable to genotypes 16 and/or 18, targeted by first-generation HPV vaccines (Gardasil® and Cervarix®), and 90% to 16, 18, 31, 33, 45, 52 or 58, targeted by the 9-valent vaccine (Gardasil® 9) [5]. These genotypes also contribute to the majority of HPV-related vulvar [6-8] and vaginal cancers [7,8].

At the country level, decision-makers are likely to seek data on the local genotype-specific burden of HPV-related diseases for baseline information against which the impact of HPV vaccination may be assessed. Focusing on the 7 high-risk (HR) genotypes covered by the 9-valent vaccine (16, 18, 31, 33, 45, 52 and 58), we investigated, by country, the availability of published literature on HPV genotypes in cervical, vaginal and vulvar cancers and intraepithelial neoplasms (CINs, VaINs and VINs) and on prevalence and incidence of genital HPV infections among women without clinically manifest disease.

## Methods

The primary sources of data were the National Library of Medicine’s PubMed/Medline and EMBASE databases. Bibliographies were screened to identify additional sources of information. The literature searches covered literature published from 2000 to May 2014; key search terms included epidemiology, human papillomavirus, genotyping, cervix, vagina, vulva, and intraepithelial lesions, cancer, in various combinations.

Publications meeting these criteria were included: original study or meta-analysis reporting (1) HPV genotype distribution in histologically confirmed CINs, VINs or VaINs or cervical, vulvar or vaginal cancers, or (2) genotype-specific genital HPV prevalence or incidence in women without clinically manifest disease (i.e., general populations, screening populations, women with normal cervical cytology, university students, convenience samples). Studies were included if they reported on genotypes 16 and 18, and at least one of the additional HR genotypes covered by the 9-valent vaccine (31, 33, 45, 52, 58). Key exclusion criteria were: publication not in English, small study (n < 50) for prevalence and cervical lesions/cancer studies, special populations (e.g., age <13 or >45 years, pregnant women, sex workers, HIV-infected, immunocompromised), duplicate publication. For each study, data was systematically extracted for country, world region, type and date of study, type of population, age, lesion type, sample type and collection method, HPV assay, number of subjects with valid HPV test results, and HPV genotyping results.

Study selection and data extraction were performed by three investigators. One of these investigators performed quality control for accuracy and conformity with selection criteria.

Availability of HPV genotyping data was compiled by world region and country [9]. For HPV prevalence studies (in women without clinically manifest disease), in addition to all studies with at least 50 subjects, availability of studies with at least 500 subjects was assessed, as larger studies provide more precise estimates of the prevalence of rare HPV infections. Maps were created using StatPlanet Plus (Version 3.21) visualization software (StatSilk, Melbourne, Australia; http://www.statsilk.com/software/statplanet).

## Results

**Figure 1:**
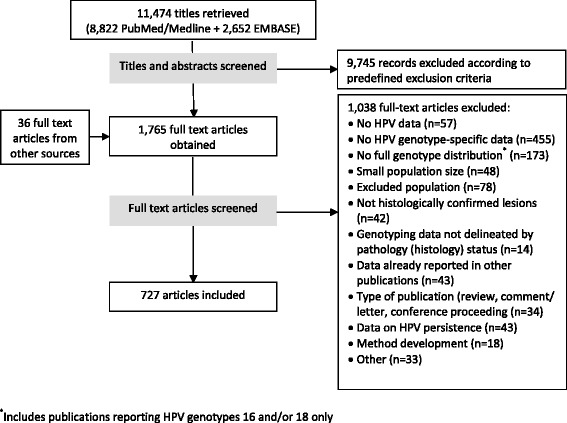
PRISMA diagram of study selection.

The literature searches yielded 11,474 records, of which 1,765 were screened in full-text and 727 included (Figure 1, Additional file 1). For full-text publications, the most common reason for exclusion was absence of HPV-genotype specific data.

Over one third (n = 256) of all publications originated from just six countries (China, Italy, United States, Brazil, South Korea and Japan).

**Figure 2:**
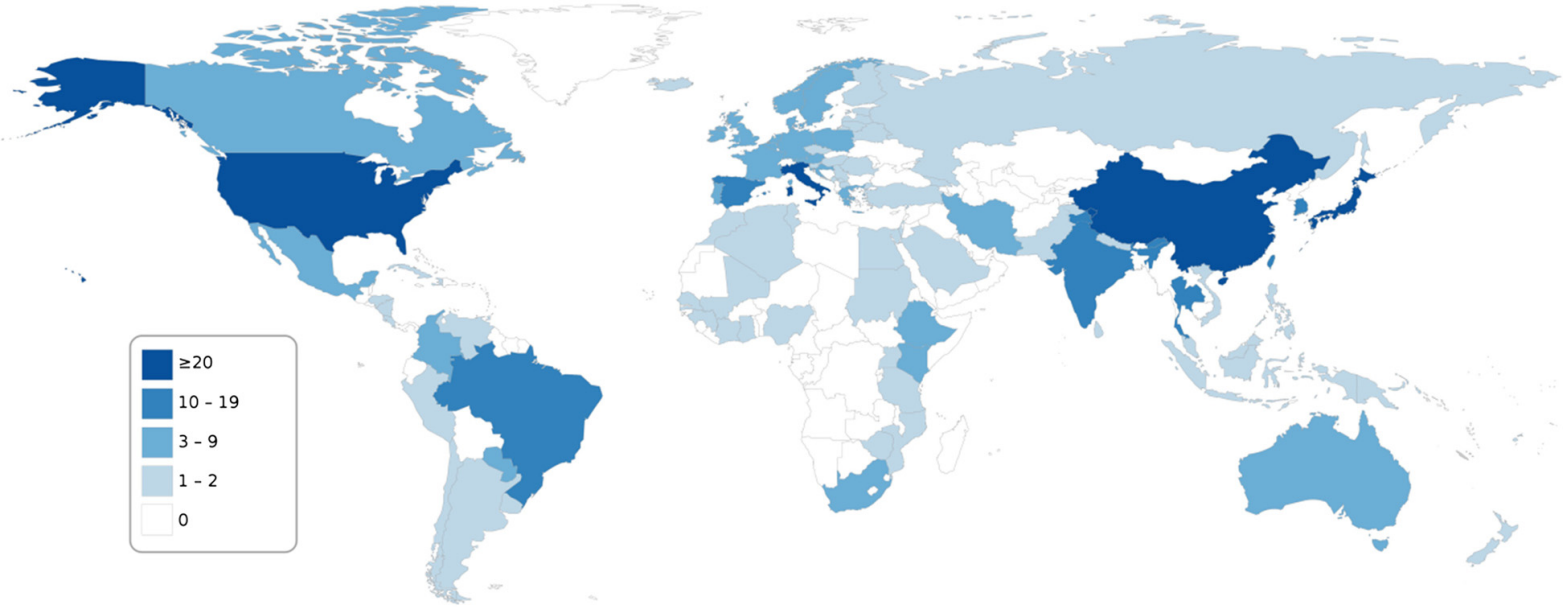
Number of publications, by country, reporting HPV genotype distribution in at least 50 cases of histologically confirmed cervical lesions (CINs) or cervical cancers. Original studies published between January 2000 and May 2014; reporting prevalence data for HPV genotypes 16 and 18 and at least one of genotypes 31, 33, 45, 52 or 58; ≥50 subjects with valid HPV test results; full-text English language publication. CIN: cervical intraepithelial lesion.

A large body of data (366 publications, Table 1) was available for HPV genotype distribution in at least 50 cervical cancers or CINs, with ten or more studies identified for ten countries (Brazil, China, India, Italy, Japan, Spain, South Korea, Thailand, Taiwan, the United States) (Figure 2). Conversely, no or few (0–2) such studies were identified for Central and Western Asia, most of Africa, particularly Central Africa, and several countries in Europe (mainly Eastern Europe), in Latin America and the Caribbean, and in South and South-East Asia.

**Table 1:** Distribution of included publications by world region and type of data*

**Category of HPV type-specific data**	**Africa**	**Asia/Pacific**	**Europe**	**Latin America and the Caribbean**	**Northern America**	**Multiple world regions**	**Globally**
**Any**	62	236	236	104	74	19	727
**Genital prevalence**	38	123	126	65	39	7	395
**Genital incidence**	4	3	5	3	6	0	21
**Cervical lesions or cancers**	30	142	107	52	25	11	366
**Vulvar or vaginal lesions or cancers**	1	4	23	2	9	4	43

Note: Sums of individual columns or rows exceed the total because several publications reported on multiple world regions or types of data.

*Including meta-analyses.

**Figure 3:**
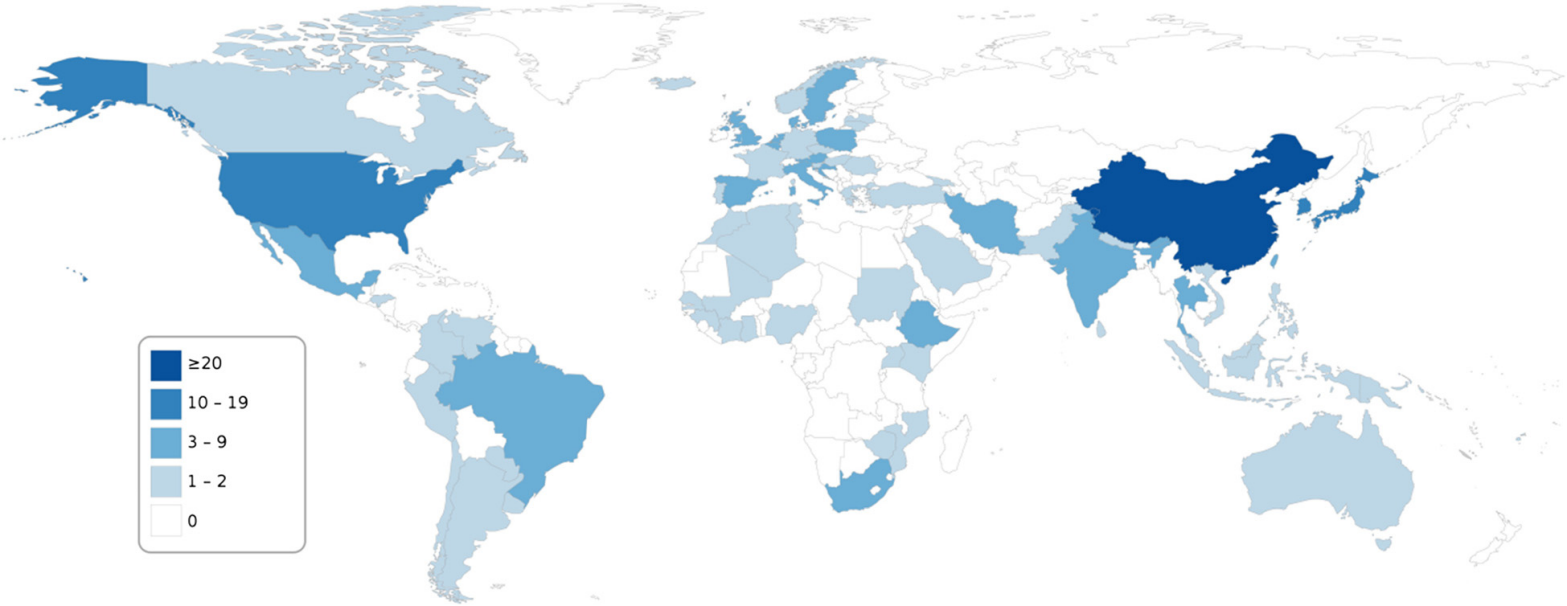
Number of publications, by country, reporting HPV genotype distribution in at least 50 cases of histologically confirmed cervical cancers. Original studies published between January 2000 and May 2014; reporting prevalence data for HPV genotypes 16 and 18 and at least one of genotypes 31, 33, 45, 52 or 58; ≥50 subjects with valid HPV test results; full-text English language publication.

For cervical cancer specifically, 208 publications from 73 countries were identified that reported HPV genotype distribution in at least 50 cases (Figure 3), and four countries (China, Japan, South Korea and the United States) had ten or more such publications. Among the 10 most populous countries, substantial cervical cancer data (≥8 publications) was available for China, the United States, Japan, India, and Brazil, but very limited data (0–2 publications) for Indonesia, Pakistan, Nigeria, Bangladesh and Russia (Figure 3).

Forty-three publications were retrieved for vulvar and vaginal cancers and VINs/VaINs. More than half (n = 23) of these studies originated from Europe, with very few available from Africa, Asia/Pacific and Latin America and the Caribbean (Table 1). Among four studies reporting vulvar and vaginal data across world regions, two were meta-analyses [7,8], pooling already existing data, and two were cross-sectional studies that generated new data but did not report country-specific findings [6,10].

**Figure 4:**
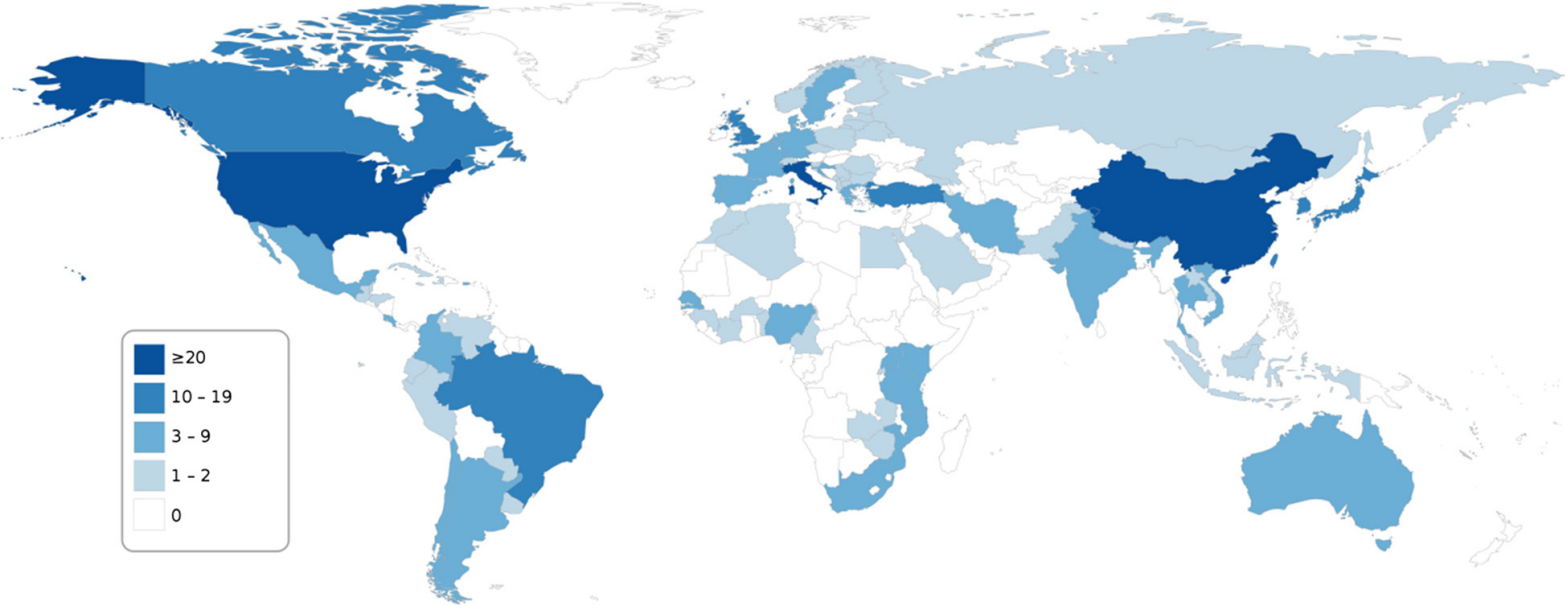
Number of publications, by country, reporting HPV genotype-specific genital prevalence among at least 50 women without clinically manifest disease*. Original studies published between January 2000 and May 2014; reporting prevalence data for HPV genotypes 16 and 18 and at least one of genotypes 31, 33, 45, 52 or 58; ≥50 subjects with valid HPV DNA test results; full-text English language publication. *General populations, screening populations, women with normal cervical cytology, or convenience samples.

A total of 395 publications reported HPV genotype-specific genital prevalence among women without clinically manifest disease. (Most of these referred to cervical infections and a small number to vaginal infections.) For ten countries, including Brazil and Turkey, ten or more such publications were available (Figure 4). However, few publications (0–2) were identified for most countries in Africa (except for those in Eastern Africa as well as Nigeria, Senegal and South Africa), Western Asia, Central Asia, South Asia (except India and Iran), South-East Asia (except Thailand and Vietnam), and Eastern Europe.

**Figure 5:**
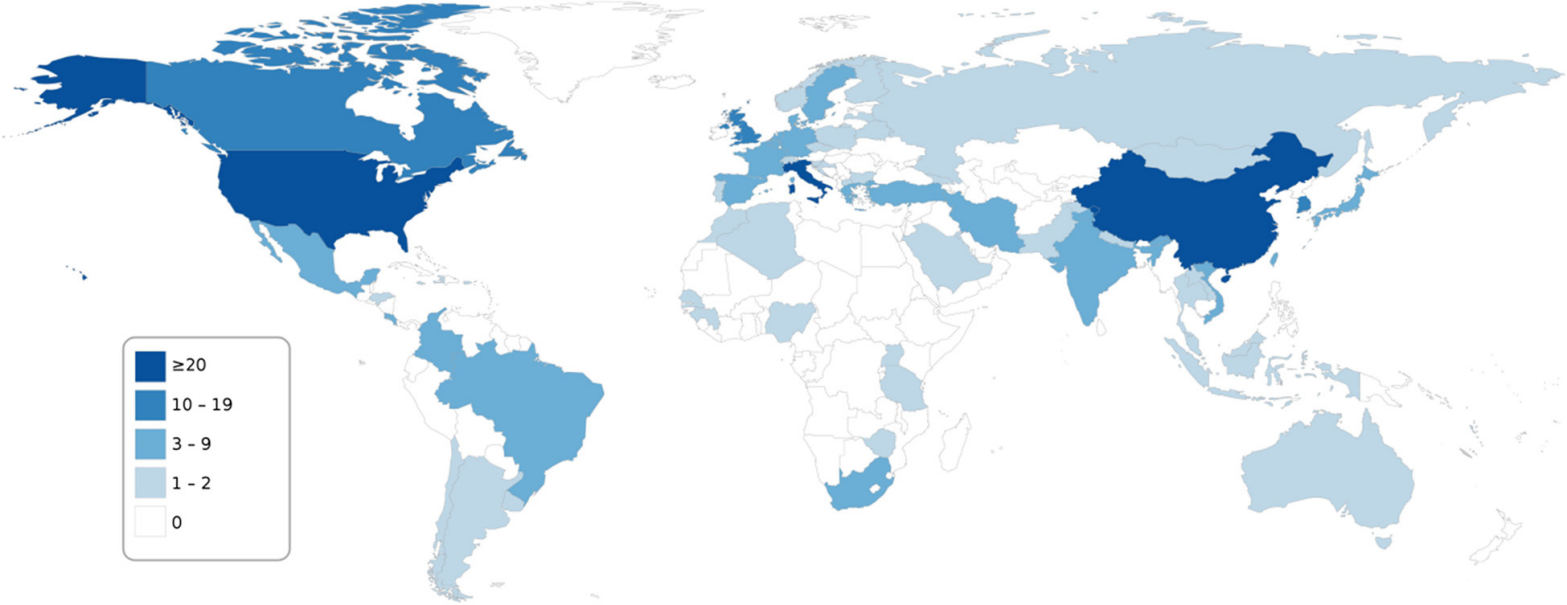
Number of publications, by country, reporting HPV genotype-specific genital prevalence among at least 500 women without clinically manifest disease*. Original studies published between January 2000 and May 2014; reporting prevalence data for HPV genotypes 16 and 18 and at least one of genotypes 31, 33, 45, 52 or 58; ≥500 subjects with valid HPV DNA test results; full-text English language publication. *General populations, screening populations, women with normal cervical cytology, or convenience samples.

A total of 238 larger prevalence studies, reporting genotype-specific prevalence in at least 500 subjects without clinically manifest disease, were identified for 68 countries, which, however, excluded most countries in Africa (Figure 5), a finding similar to an earlier report on Sub-Saharan Africa [11]. Further, no such large studies were identified for Central Asia and some countries in Latin America and the Caribbean, Europe, and Asia (Figure 5).

Only 21 publications were identified that reported type-specific incidence data in women without clinically manifest disease, 3 to 6 per world region (Table 1).

## Discussion and conclusions

Although region- or country-specific differences with respect to individual HPV genotypes exist, a solid body of research indicates that in every world region, taken together, genotypes 16/18/31/33/45/52/58 are responsible for approximately 90% of cervical cancers [12,13]. Nevertheless, decision-makers may seek local data to confirm that certain, maybe less common, vaccine-targeted genotypes circulate in their population, to assess the potential public health impacts of different HPV vaccination options, or to monitor the outcomes of vaccination programs in their jurisdiction.

Addressing this need, this study has uncovered a large body of published type-specific data but has also identified significant gaps for several world regions, including those with the highest age-standardized incidence rates of cervical cancer (ASR). Sub-regions that have a notable scarcity of cervical cancer/CIN genotyping data relative to their cervical cancer burden, as estimated by GLOBOCAN 2012 [4], include Eastern Africa (ASR per 100,000 women-years = 42.7), Middle Africa (30.6), Western Africa (29.3), Central America (23.5), South-Eastern Asia (16.3), South-Central Asia including India (19.3), and Central and Eastern Europe (16.3). An additional three sub-regions (Southern Africa, the Caribbean, and South America) have high cervical cancer ASRs (>20) but only a moderate quantity of typing data. There was also scarcity of data for vulvar/vaginal disease, outside of Europe and North America. Globally, cervical cancer ASRs are higher in less developed (15.7) compared to more developed regions (9.9) [4]. Clearly, most regions with the largest discrepancy between cervical cancer burden and availability of genotyping data belong to the less developed subset, where lack of screening and treatment of CIN are major drivers of cervical cancer incidence, and limited research infrastructure and funding are barriers for conducting HPV genotyping studies [14].

An additional finding is that, although there may be ample cervical lesion typing and prevalence data in many jurisdictions, genotype-specific incidence data is limited to few available prospective studies. Data, such as age-specific rates of HPV acquisition, are needed for informing dynamic models to obtain more accurate predictions of the population health impact of vaccination.

This study has both strengths and limitations. We did not include anal disease in our study, as anal cancer affects both genders and we intended to focus on females. However, as the burden of anal cancer is reported to be rising in Western countries [15], genotype-specific data on this HPV-related disease may become more important for decision-makers. Consistent with our objective of gauging the quantity of type-specific HPV data available, we excluded studies that reported non-type-specific data or types 16 or 18 only, which represent a large volume of published research in this field (see Figure 1). Also, inclusion was limited to published, English-language, peer-reviewed studies; thus, studies written in local languages or published in report format, for example on government websites, were not included. Limiting inclusion to peer-reviewed publications guarantees a minimum level of quality in the methods and reporting of the studies selected for our analysis. Inclusion of data from histologically-confirmed cervical lesions only (rather than cytologically defined lesions) is also consistent with our focus on higher-quality studies.

In conclusion, although a large body of HPV genotype-specific data on distribution in CINs and cervical cancers and female genital prevalence is available, significant data gaps exist for multiple world regions as well as for type-specific incidence.

## Additional file

Additional file 1: **Supplementary Material.** Full list of 727 references by world region.

## Abbreviations

CIN: Cervical intraepithelial neoplasms
HPV: Human papillomavirus
VaIN: Vaginal intraepithelial neoplasia
VIN: Vulvar intraepithelial neoplasia
