# Supplementary material for: Global availability of data on HPV genotype-distribution in cervical, vulvar and vaginal disease and genotype-specific prevalence and incidence of HPV infection in females
Source: Infect Agent Cancer. 2015 Apr 28;10:13. doi: 10.1186/s13027-015-0008-y (PMC4435914; doi:10.1186/s13027-015-0008-y)
Supplement: Supplementary file 1 — Supplementary Material. Full list of 727 references by world region. [file 13027_2015_8_MOESM1_ESM.pdf]

1 SUPPLEMENTARY MATERIAL

Full list of 727 references by world region

1.1 Africa

Abate E, Aseffa A, El-Tayeb M, El-Hassan I, Yamuah L, Mihret W, et al. Genotyping of human papillomavirus in paraffin embedded cervical tissue samples from women in Ethiopia and the Sudan. J Med Virol 2013;85(2):282-7.

Abd El-Azim S, Lotfy M, Omr A. Detection of human papillomavirus genotypes in cervical intraepithelial neoplasia and invasive cancer patients: Sharkia Governorate, Egypt. Clin Lab 2011;57(5-6):363-71.

Adjorlolo-Johnson G, Unger ER, Boni-Ouattara E, Toure-Coulibaly K, Maurice C, Vernon SD, et al. Assessing the relationship between HIV infection and cervical cancer in Cote d'Ivoire: a case-control study. BMC Infect Dis 2010;10:242.

Akarolo-Anthony SN, Al-Mujtaba M, Famooto AO, Dareng EO, Olaniyan OB, Offiong R, et al. HIV associated high-risk HPV infection among Nigerian women. BMC Infect Dis 2013;13:521.

Alhamany Z, El Mzibri M, Kharbach A, Malihy A, Abouqal R, Jaddi H, et al. Prevalence of human papillomavirus genotype among Moroccan women during a local screening program. J Infect Dev Ctries 2010;4(11):732-9.

Allan B, Marais DJ, Hoffman M, Shapiro S, Williamson AL. Cervical human papillomavirus (HPV) infection in South African women: implications for HPV screening and vaccine strategies. J Clin Microbiol 2008;46(2):740-2.

Attoh S, Asmah R, Wiredu EK, Gyasi R, Tettey Y. Human papilloma virus genotypes in Ghanaian women with cervical carcinoma. East Afr Med J 2010;87(8):345-9.

Baay MF, Kjetland EF, Ndhlovu PD, Deschoolmeester V, Mduluza T, Gomo E, et al. Human papillomavirus in a rural community in Zimbabwe: the impact of HIV co-infection on HPV genotype distribution. J Med Virol 2004;73(3):481-5.

Banura C, Franceschi S, Doorn LJ, Arslan A, Wabwire-Mangen F, Mbidde EK, et al. Infection with human papillomavirus and HIV among young women in Kampala, Uganda. J Infect Dis 2008;197(4):555-62.

Banura C, Sandin S, Van Doorn LJ, Quint W, Kleter B, Wabwire-Mangen F, et al. Type-specific incidence, clearance and predictors of cervical human papillomavirus infections (HPV) among young women: a prospective study in Uganda. Infect Agent Cancer 2010;5:7.

Bayo S, Bosch FX, de Sanjose S, Munoz N, Combita AL, Coursaget P, et al. Risk factors of invasive cervical cancer in Mali. Int J Epidemiol 2002;31(1):202-9.

Bekele A, Baay M, Mekonnen Z, Suleman S, Chatterjee S. Human papillomavirus type distribution among women with cervical pathology - a study over 4 years at Jimma Hospital, southwest Ethiopia. Trop Med Int Health 2010;15(8):890-3.

Castellsague X, Menendez C, Loscertales MP, Kornegay JR, dos SF, Gomez-Olive FX, et al. Human papillomavirus genotypes in rural Mozambique. Lancet 2001;358(9291):1429-30.

Castellsague X, Klaustermeier J, Carrilho C, Albero G, Sacarlal J, Quint W, et al. Vaccine-related HPV genotypes in women with and without cervical cancer in Mozambique: burden and potential for prevention. Int J Cancer 2008;122(8):1901-4.

Dartell M, Rasch V, Kahesa C, Mwaiselage J, Ngoma T, Junge J, et al. Human papillomavirus prevalence and type distribution in 3603 HIV-positive and HIV-negative women in the general population of Tanzania: the PROTECT study. Sex Transm Dis 2012;39(3):201-8.

De Vuyst H, Steyaert S, Van Renterghem L, Claeys P, Muchiri L, Sitati S, et al. Distribution of human papillomavirus in a family planning population in Nairobi, Kenya. Sex Transm Dis 2003;30(2):137-42.

De Vuyst H, Gichangi P, Estambale B, Njuguna E, Franceschi S, Temmerman M. Human papillomavirus types in women with invasive cervical carcinoma by HIV status in Kenya. Int J Cancer 2008;122(1):244-6.

De Vuyst H, Parisi MR, Karani A, Mandaliya K, Muchiri L, Vaccarella S, et al. The prevalence of human papillomavirus infection in Mombasa, Kenya. Cancer Causes Control 2010;21(12):2309-13.

De Vuyst H, Ndirangu G, Moodley M, Tenet V, Estambale B, Meijer CJ, et al. Prevalence of human papillomavirus in women with invasive cervical carcinoma by HIV status in Kenya and South Africa. Int J Cancer 2012;131(4):949-55.

Denny L, Adewole I, Anorlu R, Dreyer G, Moodley M, Smith T, et al. Human papillomavirus prevalence and type distribution in invasive cervical cancer in sub-Saharan Africa. Int J Cancer 2014;134(6):1389-98.

Desruisseau AJ, Schmidt-Grimminger D, Welty E. Epidemiology of HPV in HIV-positive and HIV-negative fertile women in Cameroon, West Africa. Infect Dis Obstet Gynecol 2009;2009:810596.

Ezechi OC, Ostergren PO, Nwaokorie FO, Ujah IA, Odberg PK. The burden, distribution and risk factors for cervical oncogenic human papilloma virus infection in HIV positive Nigerian women. Virol J 2014;11(1):5.

Famooto A, Almujtaba M, Dareng E, Akarolo-Anthony S, Ogbonna C, Offiong R, et al. RPS19 and TYMS SNPs and Prevalent High Risk Human Papilloma Virus Infection in Nigerian Women. PLoS One 2013;8(6):e66930.

Fanta BE. The distribution of Human Papilloma Virus infection in women with cervical histological abnormalities from an area with high incidence of cervical cancer. Ethiop Med J 2005;43(3):151-8.

Fukuchi E, Sawaya GF, Chirenje M, Magure T, Tuveson J, Ma Y, et al. Cervical Human Papillomavirus Incidence and Persistence in a Cohort of HIV-Negative Women in Zimbabwe. Sex Transm Dis 2009;36(5):305-11.

Gage JC, Ajenifuja KO, Wentzensen NA, Adepiti AC, Eklund C, Reilly M, et al. The age-specific prevalence of human papillomavirus and risk of cytologic abnormalities in rural Nigeria: implications for screen-and-treat strategies. Int J Cancer 2012;130(9):2111-7.

Hammouda D, Munoz N, Herrero R, Arslan A, Bouhadef A, Oublil M, et al. Cervical carcinoma in Algiers, Algeria: human papillomavirus and lifestyle risk factors. Int J Cancer 2005;113(3):483-9.

Hammouda D, Clifford GM, Pallardy S, Ayyach G, Chekiri A, Boudrich A, et al. Human papillomavirus infection in a population-based sample of women in Algiers, Algeria. Int J Cancer 2011;128(9):2224-9.

Hanisch RA, Sow PS, Toure M, Dem A, Dembele B, Toure P, et al. Influence of HIV-1 and/or HIV-2 infection and CD4 count on cervical HPV DNA detection in women from Senegal, West Africa. J Clin Virol 2013;58(4):696-702.

Houlihan CF, de SS, Baisley K, Changalucha J, Ross DA, Kapiga S, et al. Prevalence of Human Papillomavirus in Adolescent Girls Before Reported Sexual Debut. J Infect Dis 2014;210(6):837-45.

Jaquet A, Horo A, Charbonneau V, Ekouevi DK, Roncin L, Toure B, et al. Cervical human papillomavirus and HIV infection in women of child-bearing age in Abidjan, Cote d'Ivoire, 2010. Br J Cancer 2012;107(3):556-63.

Jones HE, Allan BR, van de Wijgert JH, Altini L, Taylor SM, de Kock A, et al. Agreement between self- and clinician-collected specimen results for detection and typing of high-risk human papillomavirus in specimens from women in Gugulethu, South Africa. J Clin Microbiol 2007;45(6):1679-83.

Kay P, Soeters R, Nevin J, Denny L, Dehaeck CM, Williamson AL. High prevalence of HPV 16 in South African women with cancer of the cervix and cervical intraepithelial neoplasia. J Med Virol 2003;71(2):265-73.

Keita N, Clifford GM, Koulibaly M, Douno K, Kabba I, Haba M, et al. HPV infection in women with and without cervical cancer in Conakry, Guinea. Br J Cancer 2009;101(1):202-8.

Khair MM, Mzibri ME, Mhand RA, Benider A, Benchekroun N, Fahime EM, et al. Molecular detection and genotyping of human papillomavirus in cervical carcinoma biopsies in an area of high incidence of cancer from Moroccan women. J Med Virol 2009;81(4):678-84.

KrennHrubec K, Mrad K, Sriha B, Ben Ayed F, Bottalico DM, Ostolaza J, et al. HPV types and variants among cervical cancer tumors in three regions of Tunisia. J Med Virol 2011;83(4):651-7.

Marais DJ, Constant D, Allan B, Carrara H, Hoffman M, Shapiro S, et al. Cervical human papillomavirus (HPV) infection and HPV type 16 antibodies in South African women. J Clin Microbiol 2008;46(2):732-9.

Marais DJ, Passmore JA, Denny L, Sampson C, Allan BR, Williamson AL. Cervical and oral human papillomavirus types in HIV-1 positive and negative women with cervical disease in South Africa. J Med Virol 2008;80(6):953-9.

Maranga IO, Hampson L, Oliver AW, He X, Gichangi P, Rana F, et al. HIV Infection Alters the Spectrum of HPV Subtypes Found in Cervical Smears and Carcinomas from Kenyan Women. Open Virol J 2013;7:19-27.

Mbaye EHS, Gheit T, Dem A, McKay-Chopin S, Toure-Kane NC, Mboup S, et al. Human papillomavirus infection in women in four regions of Senegal. J Med Virol 2014;86(2):248-56.

McDonald AC, Denny L, Wang C, Tsai WY, Wright TC, Jr., Kuhn L. Distribution of high-risk human papillomavirus genotypes among HIV-negative women with and without cervical intraepithelial neoplasia in South Africa. PLoS ONE 2012;7(9):e44332.

Meftah El Khair MM, Ait Mhand R, Mzibri ME, Ennaji MM. Risk factors of invasive cervical cancer in Morocco. Cell Mol Biol (Noisy -le-grand) 2009;55 Suppl:OL1175-OL1185.

Naucler P, Mabota da CF, da Costa JL, Ljungberg O, Bugalho A, Dillner J. Human papillomavirus type-specific risk of cervical cancer in a population with high human immunodeficiency virus prevalence: case-control study. J Gen Virol 2011;92(Pt 12):2784-91.

Ndiaye C, Alemany L, Ndiaye N, Kamate B, Diop Y, Odida M, et al. Human papillomavirus distribution in invasive cervical carcinoma in sub-Saharan Africa: could HIV explain the differences? Trop Med Int Health 2012;17(12):1432-40.

Ng'andwe C, Lowe JJ, Richards PJ, Hause L, Wood C, Angeletti PC. The distribution of sexually-transmitted Human Papillomaviruses in HIV positive and negative patients in Zambia, Africa. BMC Infect Dis 2007;7:77.

Odida M, de Sanjose S, Quint W, Bosch XF, Klaustermeier J, Weiderpass E. Human Papillomavirus type distribution in invasive cervical cancer in Uganda. BMC Infect Dis 2008;8:85.

Odida M, Sandin S, Mirembe F, Kleter B, Quint W, Weiderpass E. HPV types, HIV and invasive cervical carcinoma risk in Kampala, Uganda: a case-control study. Infect Agent Cancer 2011;6(1):8.

Okolo C, Franceschi S, Adewole I, Thomas JO, Follen M, Snijders PJ, et al. Human papillomavirus infection in women with and without cervical cancer in Ibadan, Nigeria. Infect Agent Cancer 2010;5(1):24.

Passmore JA, Marais DJ, Sampson C, Allan B, Parker N, Milner M, et al. Cervicovaginal, oral, and serum IgG and IgA responses to human papillomavirus type 16 in women with cervical intraepithelial neoplasia. J Med Virol 2007;79(9):1375-80.

Pegoraro RJ, Rom L, Lanning PA, Moodley M, Naiker S, Moodley J. P53 codon 72 polymorphism and human papillomavirus type in relation to cervical cancer in South African women. Int J Gynecol Cancer 2002;12(4):383-8.

Piras F, Piga M, De MA, Zannou AR, Minerba L, Perra MT, et al. Prevalence of human papillomavirus infection in women in Benin, West Africa. Virol J 2011;8:514.

Safaeian M, Kiddugavu M, Gravitt PE, Gange SJ, Ssekasanvu J, Murokora D, et al. Prevalence and risk factors for carcinogenic human papillomavirus infections in rural Rakai, Uganda. Sex Transm Infect 2008;84(4):306-11.

Safaeian M, Kiddugavu M, Gravitt PE, Gange SJ, Ssekasanvu J, Murokora D, et al. Determinants of incidence and clearance of high-risk human papillomavirus infections in rural Rakai, Uganda. Cancer Epidemiol Biomarkers Prev 2008;17(6):1300-7.

Said HMA. HPV genotypes in women with squamous intraepithelial lesions and normal cervixes participating in a community-based microbicide study in Pretoria, South Africa. J Clin Virol 2009;44(4):318-21.

Stanczuk GA, Kay P, Sibanda E, Allan B, Chirara M, Tswana SA, et al. Typing of human papillomavirus in Zimbabwean patients with invasive cancer of the uterine cervix. Acta Obstet Gynecol Scand 2003;82(8):762-6.

Tesfalul M, Simbiri K, Wheat CM, Motsepe D, Goldbach H, Armstrong K, et al. Oncogenic viral prevalence in invasive vulvar cancer specimens from human immunodeficiency virus-positive and -negative women in botswana. Int J Gynecol Cancer 2014;24(4):758-65.

Thomas JO, Herrero R, Omigbodun AA, Ojemakinde K, Ajayi IO, Fawole A, et al. Prevalence of papillomavirus infection in women in Ibadan, Nigeria: a population-based study. Br J Cancer 2004;90(3):638-45.

Veldhuijzen NJ, Dhont N, Vyankandondera J, Gasarabwe A, Busasa R, Crucitti T, et al. Prevalence and concordance of HPV, HIV, and HSV-2 in heterosexual couples in Kigali, Rwanda. Sex Transm Dis 2012;39(2):128-35.

Vidal AC, Murphy SK, Hernandez BY, Vasquez B, Bartlett JA, Oneko O, et al. Distribution of HPV genotypes in cervical intraepithelial lesions and cervical cancer in Tanzanian women. Infect Agent Cancer 2011;6(1):20.

Watson-Jones D, Baisley K, Brown J, Kavishe B, Andreasen A, Changalucha J, et al. High prevalence and incidence of human papillomavirus in a cohort of healthy young African female subjects. Sex Transm Infect 2013;89(5):358-65.

Wawer MJ, Tobian AA, Kigozi G, Kong X, Gravitt PE, Serwadda D, et al. Effect of circumcision of HIV-negative men on transmission of human papillomavirus to HIV-negative women: a randomised trial in Rakai, Uganda. Lancet 2011;377(9761):209-18.

Zohoncon TM, Bisseye C, Djigma FW, Yonli AT, Compaore TR, Sagna T, et al. Prevalence of HPV High-Risk Genotypes in Three Cohorts of Women in Ouagadougou (Burkina Faso). Mediterr J Hematol Infect Dis 2013;5(1):e2013059.

1.2 Asia-Pacific

Abe S, Miura K, Kinoshita A, Mishima H, Miura S, Yamasaki K, et al. Single human papillomavirus 16 or 52 infection and later cytological findings in Japanese women with NILM or ASC-US. J Hum Genet 2014;59(5):251-5.

Abudukadeer A, Ding Y, Niyazi M, Ababaikeli A, Abudula A. Distribution of HPV genotypes in uterine cervical lesions among the Uighur women in Xinjiang province of China. Eur J Gynaecol Oncol 2010;31(3):315-8.

Aggarwal R, Gupta S, Nijhawan R, Suri V, Kaur A, Bhasin V, et al. Prevalence of high-risk human papillomavirus infections in women with benign cervical cytology: a hospital based study from North India. Indian J Cancer 2006;43(3):110-6.

Akcali S, Goker A, Ecemis T, Kandiloglu AR, Sanlidag T. Human papilloma virus frequency and genotype distribution in a Turkish population. Asian Pac J Cancer Prev 2013;14(1):503-6.

Al-Ahdal MN, Al-Arnous WK, Bohol MF, Abuzaid SM, Shoukri MM, Elrady KS, et al. Human papillomaviruses in cervical specimens of women residing in Riyadh, Saudi Arabia: a hospital-based study. J Infect Dev Ctries 2014;8(3):320-5.

Al Awadhi R, Chehadeh W, Kapila K. Prevalence of human papillomavirus among women with normal cervical cytology in Kuwait. J Med Virol 2011;83(3):453-60.

Al-Badawi IA, Al-Suwaine A, Al-Aker M, Asaad L, Alaidan A, Tulbah A, et al. Detection and genotyping of human papilloma virus in cervical cancer specimens from Saudi patients. Int J Gynecol Cancer 2011;21(5):907-10.

Alibegashvili T, Clifford GM, Vaccarella S, Baidoshvili A, Gogiashvili L, Tsagareli Z, et al. Human papillomavirus infection in women with and without cervical cancer in Tbilisi, Georgia. Cancer Epidemiol 2011;35(5):465-70.

Alsbeih G, Ahmed R, Al Harbi N, Venturina LA, Tulbah A, Balaraj K. Prevalence and genotypes' distribution of human papillomavirus in invasive cervical cancer in Saudi Arabia. Gynecol Oncol 2011;121(3):522-6.

An HJ, Cho NH, Lee SY, Kim IH, Lee C, Kim SJ, et al. Correlation of cervical carcinoma and precancerous lesions with human papillomavirus (HPV) genotypes detected with the HPV DNA chip microarray method. Cancer 2003;97(7):1672-80.

An HJ, Kim KR, Kim IS, Kim DW, Park MH, Park IA, et al. Prevalence of human papillomavirus DNA in various histological subtypes of cervical adenocarcinoma: a population-based study. Mod Pathol 2005;18(4):528-34.

Aruhuri B, Tarivonda L, Tenet V, Sinha R, Snijders P, Clifford G, et al. Prevalence of cervical human papillomavirus (HPV) infection in Vanuatu. Cancer Prev Res (Phila) 2012;5(5):746-53.

Asato T, Maehama T, Nagai Y, Kanazawa K, Uezato H, Kariya K. A large case-control study of cervical cancer risk associated with human papillomavirus infection in Japan, by nucleotide sequencing-based genotyping. J Infect Dis 2004;189(10):1829-32.

Aydin Y, Atis A, Tutuman T, Goker N. Prevalence of human papilloma virus infection in pregnant Turkish women compared with non-pregnant women. Eur J Gynaecol Oncol 2010;31(1):72-4.

Bae JH, Lee SJ, Kim CJ, Hur SY, Park YG, Lee WC, et al. Human papillomavirus (HPV) type distribution in Korean women: a meta-analysis. J Microbiol Biotechnol 2008;18(4):788-94.

Bao YP, Li N, Smith JS, Qiao YL. Human papillomavirus type distribution in women from Asia: a meta-analysis. Int J Gynecol Cancer 2008;18(1):71-9.

Bao YP, Li N, Smith JS, Qiao YL. Human papillomavirus type-distribution in the cervix of Chinese women: a meta-analysis. Int J STD AIDS 2008;19(2):106-11.

Baser E, Ozgu E, Erkilinc S, Togrul C, Caglar M, Gungor T. Risk factors for human papillomavirus persistence among women undergoing cold-knife conization for treatment of high-grade cervical intraepithelial neoplasia. Int J Gynaecol Obstet 2014;125(3):275-8.

Basu P, Roychowdhury S, Bafna UD, Chaudhury S, Kothari S, Sekhon R, et al. Human papillomavirus genotype distribution in cervical cancer in India: results from a multi-center study. Asian Pac J Cancer Prev 2009;10(1):27-34.

Bayram A, Erkilic S, Balat O, Eksi F, Ugur MG, Ozturk E, et al. Prevalence and genotype distribution of human papillomavirus in non-neoplastic cervical tissue lesion: cervical erosion. J Med Virol 2011;83(11):1997-2003.

Bhatla N, Dar L, Patro AR, Kriplani A, Gulati A, Verma K, et al. Human papillomavirus type distribution in cervical cancer in Delhi, India. Int J Gynecol Pathol 2006;25(4):398-402.

Bhatla N, Dar L, Rajkumar PA, Kumar P, Pati SK, Kriplani A, et al. Human papillomavirus-type distribution in women with and without cervical neoplasia in north India. Int J Gynecol Pathol 2008;27(3):426-30.

Bhatla N, Lal N, Bao YP, Ng T, Qiao YL. A meta-analysis of human papillomavirus type-distribution in women from South Asia: implications for vaccination. Vaccine 2008;26(23):2811-7.

Bhattarakosol P, Lertworapreecha M, Kitkumthorn N, Triratanachai S, Niruthisard S. Survey of human papillomavirus infection in cervical intraepithelial neoplasia in Thai women. J Med Assoc Thai 2002;85 Suppl 1:S360-S365.

Brestovac B, Harnett GB, Smith DW, Shellam GR, Frost FA. Human papillomavirus genotypes and their association with cervical neoplasia in a cohort of Western Australian women. J Med Virol 2005;76(1):106-10.

Brotherton JM. How much cervical cancer in Australia is vaccine preventable? A meta-analysis. Vaccine 2008;26(2):250-6.

Cai HB, Ding XH, Zhou YF, Lie DM. Risk factors for cervical cancer in China: a case-control study. Eur J Gynaecol Oncol 2008;29(1):72-5.

Cai HB, Ding XH, Chen CC. Prevalence of single and multiple human papillomavirus types in cervical cancer and precursor lesions in Hubei, China. Oncology 2009;76(3):157-61.

Chan PK, Chang AR, Cheung JL, Chan DP, Xu LY, Tang NL, et al. Determinants of cervical human papillomavirus infection: differences between high- and low-oncogenic risk types. J Infect Dis 2002;185(1):28-35.

Chan PK, Ho WC, Yu MY, Pong WM, Chan AC, Chan AK, et al. Distribution of human papillomavirus types in cervical cancers in Hong Kong: current situation and changes over the last decades. Int J Cancer 2009;125(7):1671-7.

Chan PK, Chang AR, Yu MY, Li WH, Chan MY, Yeung AC, et al. Age distribution of human papillomavirus infection and cervical neoplasia reflects caveats of cervical screening policies. Int J Cancer 2010;126(1):297-301.

Chan PK, Cheung TH, Li WH, Yu MY, Chan MY, Yim SF, et al. Attribution of human papillomavirus types to cervical intraepithelial neoplasia and invasive cancers in Southern China. Int J Cancer 2012;131(3):692-705.

Chansaenroj J, Lurchachaiwong W, Termrungruanglert W, Tresukosol D, Niruthisard S, Trivijitsilp P, et al. Prevalence and genotypes of human papillomavirus among Thai women. Asian Pac J Cancer Prev 2010;11(1):117-22.

Chansaenroj J, Junyangdikul P, Chinchai T, Swangvaree S, Karalak A, Gemma N, et al. Large scale study of HPV genotypes in cervical cancer and different cytological cervical specimens in Thailand. J Med Virol 2014;86(4):601-7.

Chao A, Hsu KH, Lai CH, Huang HJ, Hsueh S, Lin SR, et al. Cervical cancer screening program integrating Pap smear and HPV DNA testing: a population-based study. Int J Cancer 2008;122(12):2835-41.

Chao A, Chang CJ, Lai CH, Chao FY, Hsu YH, Chou HH, et al. Incidence and outcome of acquisition of human papillomavirus infection in women with normal cytology--a population-based cohort study from Taiwan. Int J Cancer 2010;126(1):191-8.

Chao A, Jao MS, Huang CC, Huang HJ, Cheng HH, Yang JE, et al. Human papillomavirus genotype in cervical intraepithelial neoplasia grades 2 and 3 of Taiwanese women. Int J Cancer 2011;128(3):653-9.

Chao A, Chen TC, Hsueh C, Huang CC, Yang JE, Hsueh S, et al. Human papillomavirus in vaginal intraepithelial neoplasia. Int J Cancer 2012;131(3):E259-68.

Chen CA, Liu CY, Chou HH, Chou CY, Ho CM, Twu NF, et al. The distribution and differential risks of human papillomavirus genotypes in cervical preinvasive lesions: A Taiwan Cooperative Oncologic Group Study. Int J Gynecol Cancer 2006;16(5):1801-8.

Chen HC, You SL, Hsieh CY, Schiffman M, Lin CY, Pan MH, et al. Prevalence of genotype-specific human papillomavirus infection and cervical neoplasia in Taiwan: a community-based survey of 10,602 women. Int J Cancer 2011;128(5):1192-203.

Chen HC, Schiffman M, Lin CY, Pan MH, You SL, Chuang LC, et al. Persistence of type-specific human papillomavirus infection and increased long-term risk of cervical cancer. J Natl Cancer Inst 2011;103(18):1387-96.

Chen Q, Xie LX, Qing ZR, Li LJ, Luo ZY, Lin M, et al. Epidemiologic characterization of human papillomavirus infection in rural Chaozhou, Eastern Guangdong province of China. PLoS ONE 2012;7(2):e32149.

Chen Q, Luo ZY, Lin M, Lin QL, Chen CY, Yang C, et al. Prevalence and genotype distribution of human papillomavirus infections in women attending hospitals in Chaozhou of Guangdong province. Asian Pac J Cancer Prev 2012;13(4):1519-24.

Chen W, Zhang X, Molijn A, Jenkins D, Shi JF, Quint W, et al. Human papillomavirus type-distribution in cervical cancer in China: the importance of HPV 16 and 18. Cancer Causes Control 2009;20(9):1705-13.

Chen Z, Meng W, Du R, Zhu Y, Zhang Y, Ding Y. Genotype distribution and the relative risk factors for human papillomavirus in Urumqi, China. Exp Ther Med 2013;6(1):85-90.

Chiang YC, Cheng WF, Chen YL, Chang MC, Hsieh CY, Lin MC, et al. High-risk human papillomavirus, other than type 16/18, in predominantly older Taiwanese women with high-grade cervical preinvasive lesions. Taiwan J Obstet Gynecol 2013;52(2):222-6.

Chinchai T, Chansaenroj J, Swangvaree S, Junyangdikul P, Poovorawan Y. Prevalence of human papillomavirus genotypes in cervical cancer. Int J Gynecol Cancer 2012;22(6):1063-8.

Cho NH, An HJ, Jeong JK, Kang S, Kim JW, Kim YT, et al. Genotyping of 22 human papillomavirus types by DNA chip in Korean women: comparison with cytologic diagnosis. Am J Obstet Gynecol 2003;188(1):56-62.

Choi MC, Jung SG, Park H, Lee SY, Lee C, Hwang YY, et al. Photodynamic therapy for management of cervical intraepithelial neoplasia II and III in young patients and obstetric outcomes. Lasers Surg Med 2013;45(9):564-72.

Chong PP, Asyikin N, Rusinahayati M, Halimatun S, Rozita R, Ng CK, et al. High prevalence of human papillomavirus DNA detected in cervical swabs from women in southern Selangor, Malaysia. Asian Pac J Cancer Prev 2010;11(6):1645-51.

Chui SH, Szeto YT, Lam CW. Human papillomavirus infection in Macau women. Public Health 2012;126(7):600-4.

Dai M, Bao YP, Li N, Clifford GM, Vaccarella S, Snijders PJ, et al. Human papillomavirus infection in Shanxi Province, People's Republic of China: a population-based study. Br J Cancer 2006;95(1):96-101.

Datta P, Bhatla N, Dar L, Patro AR, Gulati A, Kriplani A, et al. Prevalence of human papillomavirus infection among young women in North India. Cancer Epidemiol 2010;34(2):157-61.

Datta P, Bhatla N, Pandey RM, Dar L, Patro AR, Vasisht S, et al. Type-specific incidence and persistence of HPV infection among young women: a prospective study in North India. Asian Pac J Cancer Prev 2012;13(3):1019-24.

Das D, Rai AK, Kataki AC, Barmon D, Deka P, Sharma JD, et al. Nested multiplex PCR based detection of human papillomavirus in cervical carcinoma patients of north- East India. Asian Pac J Cancer Prev 2013;14(2):785-90.

Demir ET, Ceyhan M, Simsek M, Gunduz T, Arlier S, Aytac R, et al. The prevalence of different HPV types in Turkish women with a normal Pap smear. J Med Virol 2012;84(8):1242-7.

Deodhar K, Gheit T, Vaccarella S, Romao CC, Tenet V, Nene BM, et al. Prevalence of human papillomavirus types in cervical lesions from women in rural Western India. J Med Virol 2012;84(7):1054-60.

Ding DC, Hsu HC, Huang RL, Lai HC, Lin CY, Yu MH, et al. Type-specific distribution of HPV along the full spectrum of cervical carcinogenesis in Taiwan: an indication of viral oncogenic potential. Eur J Obstet Gynecol Reprod Biol 2008;140(2):245-51.

Dondog B, Clifford GM, Vaccarella S, Waterboer T, Unurjargal D, Avirmed D, et al. Human papillomavirus infection in Ulaanbaatar, Mongolia: a population-based study. Cancer Epidemiol Biomarkers Prev 2008;17(7):1731-8.

Dursun P, Senger SS, Arslan H, Kuscu E, Ayhan A. Human papillomavirus (HPV) prevalence and types among Turkish women at a gynecology outpatient unit. BMC Infect Dis 2009;9:191.

Eghbali SS, Amirinejad R, Obeidi N, Mosadeghzadeh S, Vahdat K, Azizi F, et al. Oncogenic human papillomavirus genital infection in southern Iranian women: population-based study versus clinic-based data. Virol J 2012;9:194.

Ekalaksananan T, Pientong C, Kotimanusvanij D, Kongyingyoes B, Sriamporn S, Jintakanon D. The relationship of human papillomavirus (HPV) detection to pap smear classification of cervical-scraped cells in asymptomatic women in northeast Thailand. J Obstet Gynaecol Res 2001;27(3):117-24.

Ekalaksananan T, Pientong C, Thinkhamrop J, Kongyingyoes B, Evans MF, Chaiwongkot A. Cervical cancer screening in north east Thailand using the visual inspection with acetic acid (VIA) test and its relationship to high-risk human papillomavirus (HR-HPV) status. J Obstet Gynaecol Res 2010;36(5):1037-43.

Eren F, Erenus M, Bas E, Ahiskali R, Yoldemir T. Prevalence of HPV infection by cytologic diagnosis and HPV DNA extraction and prevalence of the HPV genotypes detected in urban Turkish women. Int J Gynaecol Obstet 2010;109(3):235-8.

Esmaeili M, Bonyadi M, Dastranj A, Alizadeh M, Melli MS, Shobeiri MJ. HPV typing in women with cervical precancerous and cancerous lesions in northwestern Iran. Gynecol Obstet Invest 2008;66(1):68-72.

Ferdousi J, Nagai Y, Asato T, Hirakawa M, Inamine M, Kudaka W, et al. Impact of human papillomavirus genotype on response to treatment and survival in patients receiving radiotherapy for squamous cell carcinoma of the cervix. Exp Ther Med 2010;1(3):525-30.

Franceschi S, Rajkumar T, Vaccarella S, Gajalakshmi V, Sharmila A, Snijders PJ, et al. Human papillomavirus and risk factors for cervical cancer in Chennai, India: a case-control study. Int J Cancer 2003;107(1):127-33.

Franceschi S, Rajkumar R, Snijders PJ, Arslan A, Mahe C, Plummer M, et al. Papillomavirus infection in rural women in southern India. Br J Cancer 2005;92(3):601-6.

Futai M, Watanabe J, Jobo T, Tsunoda S, Nishimura Y, Watanabe K, et al. Clinical significance of human papillomavirus genotype by linear array assay in Japanese women with uterine cervical lesions and type 16 physical status by in situ hybridization. Int J Gynecol Cancer 2009;19(8):1396-401.

Gao YE, Zhang J, Wu J, Chen ZC, Yan XJ. Detection and genotyping of human papillomavirus DNA in cervical cancer tissues with fluorescence polarization. Sheng Wu Hua Xue Yu Sheng Wu Wu Li Xue Bao (Shanghai) 2003;35(11):1029-34.

Garland SM, Brotherton JM, Condon JR, McIntyre PB, Stevens MP, Smith DW, et al. Human papillomavirus prevalence among indigenous and non-indigenous Australian women prior to a national HPV vaccination program. BMC Med 2011;9:104.

Ghaffari SR, Sabokbar T, Mollahajian H, Dastan J, Ramezanzadeh F, Ensani F, et al. Prevalence of human papillomavirus genotypes in women with normal and abnormal cervical cytology in Iran. Asian Pac J Cancer Prev 2006;7(4):529-32.

Gheit T, Vaccarella S, Schmitt M, Pawlita M, Franceschi S, Sankaranarayanan R, et al. Prevalence of human papillomavirus types in cervical and oral cancers in central India. Vaccine 2009;27(5):636-9.

Guo J, Zhao F, Liu R, Mu Y. Prevalence and type distribution of human papillomavirus infection in women from Datong, China. Scand J Infect Dis 2010;42(1):72-5.

Haghshenas M, Golini-Moghaddam T, Rafiee A, Emadeian O, Shykhpour A, Ashrafi GH. Prevalence and type distribution of high-risk human papillomavirus in patients with cervical cancer: a population-based study. Infect Agent Cancer 2013;8(1):20.

Hajjaj AA, Senok AC, Al Mahmeed AE, Issa AA, Arzese AR, Botta GA. Human papillomavirus infection among women attending health facilities in the Kingdom of Bahrain. Saudi Med J 2006;27(4):487-91.

Hamzi Abdul RS, Isa NM, Zailani HA, Omar B, Abdullah MF, Mohd Amin WA, et al. Distribution of HPV genotypes in cervical cancer in multi- ethnic Malaysia. Asian Pac J Cancer Prev 2014;15(2):651-6.

Harima Y, Sawada S, Nagata K, Sougawa M, Ohnishi T. Human papilloma virus (HPV) DNA associated with prognosis of cervical cancer after radiotherapy. Int J Radiat Oncol Biol Phys 2002;52(5):1345-51.

Hlaing T, Yip YC, Ngai KL, Vong HT, Wong SI, Ho WC, et al. Distribution of human papillomavirus genotypes among cervical intraepithelial neoplasia and invasive cancers in Macao. J Med Virol 2010;82(9):1600-5.

Ho CM, Chien TY, Huang SH, Lee BH, Chang SF. Integrated human papillomavirus types 52 and 58 are infrequently found in cervical cancer, and high viral loads predict risk of cervical cancer. Gynecol Oncol 2006;102(1):54-60.

Hong D, Ye F, Chen H, Lu W, Cheng Q, Hu Y, et al. Distribution of human papillomavirus genotypes in the patients with cervical carcinoma and its precursors in Zhejiang Province, China. Int J Gynecol Cancer 2008;18(1):104-9.

Hou R, Xu C, Zhang S, Wu M, Zhang W. Distribution of human papillomavirus genotype and cervical neoplasia among women with abnormal cytology in Beijing, China. Int J Gynaecol Obstet 2012;119(3):257-61.

Huang HJ, Huang SL, Lin CY, Lin RW, Chao FY, Chen MY, et al. Human papillomavirus genotyping by a polymerase chain reaction-based genechip method in cervical carcinoma treated with neoadjuvant chemotherapy plus radical surgery. Int J Gynecol Cancer 2004;14(4):639-49.

Huang LW, Chao SL, Chen PH, Chou HP. Multiple HPV genotypes in cervical carcinomas: improved DNA detection and typing in archival tissues. J Clin Virol 2004;29(4):271-6.

Huang LW, Hwang JL, Lin YH. Type-specific distribution of human papillomavirus in relation to grades of cervical neoplasia. Int J Gynaecol Obstet 2006;92(2):143-4.

Huang Y-KY. Long-term outcomes of high-risk human papillomavirus infection support a long interval of cervical cancer screening. British Journal of Cancer 2008;98(5):863-9.

Hwang HS, Park M, Lee SY, Kwon KH, Pang MG. Distribution and prevalence of human papillomavirus genotypes in routine pap smear of 2,470 korean women determined by DNA chip. Cancer Epidemiol Biomarkers Prev 2004;13(12):2153-6.

Hwang TS, Jeong JK, Park M, Han HS, Choi HK, Park TS. Detection and typing of HPV genotypes in various cervical lesions by HPV oligonucleotide microarray. Gynecol Oncol 2003;90(1):51-6.

Inaba K, Nagasaka K, Kawana K, Arimoto T, Matsumoto Y, Tsuruga T, et al. High-risk human papillomavirus correlates with recurrence after laser ablation for treatment of patients with cervical intraepithelial neoplasia 3: A long-term follow-up retrospective study. J Obstet Gynaecol Res 2014;40(2):554-60.

Inal MM, Kose S, Yildirim Y, Ozdemir Y, Toz E, Ertopcu K, et al. The relationship between human papillomavirus infection and cervical intraepithelial neoplasia in Turkish women. Int J Gynecol Cancer 2007;17(6):1266-70.

Inoue M, Sakaguchi J, Sasagawa T, Tango M. The evaluation of human papillomavirus DNA testing in primary screening for cervical lesions in a large Japanese population. Int J Gynecol Cancer 2006;16(3):1007-13.

Jeng CJ, Phdl, Ko ML, Ling QD, Shen J, Lin HW, et al. Prevalence of cervical human papillomavirus in Taiwanese women. Clin Invest Med 2005;28(5):261-6.

Jin Q, Shen K, Li H, Zhou XR, Huang HF, Leng JH. Age-specific prevalence of human papillomavirus by grade of cervical cytology in Tibetan women. Chin Med J (Engl) 2010;123(15):2004-11.

Kang WD, Kim CH, Cho MK, Kim JW, Kim YH, Choi HS, et al. Comparison of the hybrid capture II assay with the human papillomavirus DNA chip test for the detection of high-grade cervical lesions. Int J Gynecol Cancer 2009;19(5):924-8.

Kang WD, Choi HS, Kim SM. Is vaccination with quadrivalent HPV vaccine after loop electrosurgical excision procedure effective in preventing recurrence in patients with high-grade cervical intraepithelial neoplasia (CIN2-3)? Gynecol Oncol 2013;130(2):264-8.

Karunaratne K, Ihalagama H, Rohitha S, Molijn A, Gopala K, Schmidt JE, et al. Human papillomavirus prevalence and type-distribution in women with cervical lesions: a cross-sectional study in Sri Lanka. BMC Cancer 2014;14:116.

Khodakarami N, Clifford GM, Yavari P, Farzaneh F, Salehpour S, Broutet N, et al. Human papillomavirus infection in women with and without cervical cancer in Tehran, Iran. Int J Cancer 2012;131(2):E156-61.

Khorasanizadeh F, Hassanloo J, Khaksar N, Mohammad TS, Marzaban M, Rashidi H, et al. Epidemiology of cervical cancer and human papilloma virus infection among Iranian women - analyses of national data and systematic review of the literature. Gynecol Oncol 2013;128(2):277-81.

Kim CJ, Lee YS, Kwack HS, Yoon WS, Park TC, Park JS. Specific human papillomavirus types and other factors on the risk of cervical intraepithelial neoplasia: a case-control study in Korea. Int J Gynecol Cancer 2010;20(6):1067-73.

Kim MA, Oh JK, Chay DB, Park DC, Kim SM, Kang ES, et al. Prevalence and seroprevalence of high-risk human papillomavirus infection. Obstet Gynecol 2010;116(4):932-40.

Kim MJ, Kim JJ, Kim S. Type-specific prevalence of high-risk human papillomavirus by cervical cytology and age: Data from the health check-ups of 7,014 Korean women. Obstet Gynecol Sci 2013;56(2):110-20.

Kim YJ, Kwon MJ, Woo HY, Paik SY. Prevalence of human papillomavirus infection and genotype distribution determined by the cyclic-catcher melting temperature analysis in Korean medical checkup population. J Microbiol 2013;51(5):665-70.

Kondo K, Uenoyama A, Kitagawa R, Tsunoda H, Kusumoto-Matsuo R, Mori S, et al. Genotype distribution of human papillomaviruses in Japanese women with abnormal cervical cytology. Open Virol J 2012;6:277-83.

Konno R, Tamura S, Dobbelaere K, Yoshikawa H. Prevalence and type distribution of human papillomavirus in healthy Japanese women aged 20 to 25 years old enrolled in a clinical study. Cancer Sci 2011;102(4):877-82.

Lai CH, Huang HJ, Hsueh S, Chao A, Lin CT, Huang SL, et al. Human papillomavirus genotype in cervical cancer: a population-based study. Int J Cancer 2007;120(9):1999-2006.

Lai CH, Chao A, Chang CJ, Huang CC, Wang LC, Hsueh S, et al. Age factor and implication of human papillomavirus type-specific prevalence in women with normal cervical cytology. Epidemiol Infect 2012;140(3):466-73.

Laskov I, Grisaru D, Efrat G, Trejo LL, Grisaru G, Avidor B. Are the human papillomavirus genotypes different in cervical cancer and intraepithelial neoplasia in Jewish Israeli women, a low-risk population? Int J Gynecol Cancer 2013;23(4):730-4.

Lee EH, Um TH, Chi HS, Hong YJ, Cha YJ. Prevalence and distribution of human papillomavirus infection in Korean women as determined by restriction fragment mass polymorphism assay. J Korean Med Sci 2012;27(9):1091-7.

Lee GY, Kim SM, Rim SY, Choi HS, Park CS, Nam JH. Human papillomavirus (HPV) genotyping by HPV DNA chip in cervical cancer and precancerous lesions. Int J Gynecol Cancer 2005;15(1):81-7.

Lee HS, Kim KM, Kim SM, Choi YD, Nam JH, Park CS, et al. Human papillomavirus genotyping using HPV DNA chip analysis in Korean women. Int J Gynecol Cancer 2007;17(2):497-501.

Lee K-O, Jeong S-J, Park M-Y, Seong H-S, Shin E-S, Choi K-H, et al. Prevalence of human papillomavirus genotypes in routine pap smear of 2,562 Korean women determined by PCR-DNA sequencing. Journal of Bacteriology and Virology 2009;39(4):337-44.

Lee SA, Kang D, Seo SS, Jeong JK, Yoo KY, Jeon YT, et al. Multiple HPV infection in cervical cancer screened by HPVDNAChip. Cancer Lett 2003;198(2):187-92.

Lee YS, Gong G, Sohn JH, Ryu KS, Lee JH, Khang SK, et al. Cytological Evaluation and REBA HPV-ID HPV Testing of Newly Developed Liquid-Based Cytology, EASYPREP: Comparison with SurePath. Korean J Pathol 2013;47(3):265-74.

Li C, Wu M, Wang J, Zhang S, Zhu L, Pan J, et al. A population-based study on the risks of cervical lesion and human papillomavirus infection among women in Beijing, People's Republic of China. Cancer Epidemiol Biomarkers Prev 2010;19(10):2655-64.

Li H, Zhang J, Chen Z, Zhou B, Tan Y. Prevalence of human papillomavirus genotypes among women in Hunan province, China. Eur J Obstet Gynecol Reprod Biol 2013;170(1):202-5.

Li J, Xie L, Gan X, Liu B, Zhang Y, Song B, et al. Association of inhibitor of differentiation 1 expression with human papillomaviruses infections in cervical carcinoma. Int J Gynecol Cancer 2011;21(7):1276-81.

Li J, Zhang D, Zhang Y, Wang X, Lin Y, Hu L. Prevalence and genotype distribution of human papillomavirus in women with cervical cancer or high-grade precancerous lesions in Chengdu, western China. Int J Gynaecol Obstet 2011;112(2):131-4.

Li J, Mei J, Wang X, Hu L, Lin Y, Yang P. Human papillomavirus type-specific prevalence in women with cervical intraepithelial neoplasm in Western China. J Clin Microbiol 2012;50(3):1079-81.

Li LK, Dai M, Clifford GM, Yao WQ, Arslan A, Li N, et al. Human papillomavirus infection in Shenyang City, People's Republic of China: A population-based study. Br J Cancer 2006;95(11):1593-7.

Li Y, Wang Y, Jia C, Ma Y, Lan Y, Wang S. Detection of human papillomavirus genotypes with liquid bead microarray in cervical lesions of northern Chinese patients. Cancer Genet Cytogenet 2008;182(1):12-7.

Lin H, Ma YY, Moh JS, Ou YC, Shen SY, ChangChien CC. High prevalence of genital human papillomavirus type 52 and 58 infection in women attending gynecologic practitioners in South Taiwan. Gynecol Oncol 2006;101(1):40-5.

Lin M, Yang LY, Li LJ, Wu JR, Peng YP, Luo ZY. Genital human papillomavirus screening by gene chip in Chinese women of Guangdong province. Aust N Z J Obstet Gynaecol 2008;48(2):189-94.

Liu HY, Zhou SL, Ku JW, Zhang DY, Li B, Han XN, et al. Prevalence of human papillomavirus infection in esophageal and cervical cancers in the high incidence area for the two diseases from 2007 to 2009 in Linzhou of Henan Province, Northern China. Arch Virol 2014;159(6):1393-401.

Liu J, Rose B, Huang X, Liao G, Carter J, Wu X, et al. Comparative analysis of characteristics of women with cervical cancer in high- versus low-incidence regions. Gynecol Oncol 2004;94(3):803-10.

Liu SS, Tsang PC, Chan KY, Cheung AN, Chan KK, Leung RC, et al. Distribution of six oncogenic types of human papillomavirus and type 16 integration analysis in Chinese women with cervical precancerous lesions and carcinomas. Tumour Biol 2008;29(2):105-13.

Liu SS, Chan KY, Leung RC, Chan KK, Tam KF, Luk MH, et al. Prevalence and risk factors of Human Papillomavirus (HPV) infection in southern Chinese women - a population-based study. PLoS ONE 2011;6(5):e19244.

Liu W, Wu EQ, Yu XH, Feng LH, Jiang CL, Zha X, et al. Detection of human papillomavirus genotypes associated with mucopurulent cervicitis and cervical cancer in Changchun, China. Int J Gynaecol Obstet 2013;120(2):124-6.

Liu X, Zhang S, Ruan Q, Ji Y, Ma L, Zhang Y. Prevalence and type distribution of human papillomavirus in women with cervical lesions in Liaoning Province, China. Int J Gynecol Cancer 2010;20(1):147-53.

Lo KWK. Clinical and prognostic significance of human papillomavirus in a Chinese population of cervical cancers. Gynecol Obstet Invest 2001;51(3):202-10.

Lo KW, Wong YF, Chan MK, Poon JS, Wang VW, Zhu SN, et al. Prevalence of human papillomavirus in cervical cancer: A multicenter study in China. Int J Cancer 2002;100(3):327-31.

Maehama T, Asato T, Kanazawa K. Prevalence of HPV infection in cervical cytology-normal women in Okinawa, Japan, as determined by a polymerase chain reaction. Int J Gynaecol Obstet 2000;69(2):175-6.

Maehama T, Asato T, Kanazawa K. Prevalence of human papillomavirus in cervical swabs in the Okinawa Islands, Japan. Arch Gynecol Obstet 2002;267(2):64-6.

Maehama T. Epidemiological study in Okinawa, Japan, of human papillomavirus infection of the uterine cervix. Infect Dis Obstet Gynecol 2005;13(2):77-80.

Masumoto N, Fujii T, Ishikawa M, Mukai M, Ono A, Iwata T, et al. Dominant human papillomavirus 16 infection in cervical neoplasia in young Japanese women; study of 881 outpatients. Gynecol Oncol 2004;94(2):509-14.

Matsukura T, Sugase M. Relationships between 80 human papillomavirus genotypes and different grades of cervical intraepithelial neoplasia: association and causality. Virology 2001;283(1):139-47.

Matsumoto K, Oki A, Furuta R, Maeda H, Yasugi T, Takatsuka N, et al. Predicting the progression of cervical precursor lesions by human papillomavirus genotyping: a prospective cohort study. Int J Cancer 2011;128(12):2898-910.

Miura S, Matsumoto K, Oki A, Satoh T, Tsunoda H, Yasugi T, et al. Do we need a different strategy for HPV screening and vaccination in East Asia? Int J Cancer 2006;119(11):2713-5.

Mortazavi S, Zali M, Raoufi M, Nadji M, Kowsarian P, Nowroozi A. The Prevalence of Human Papillomavirus in Cervical Cancer in Iran. Asian Pac J Cancer Prev 2002;3(1):69-72.

Nagai Y, Maehama T, Asato T, Kanazawa K. Detection of human papillomavirus DNA in primary and metastatic lesions of carcinoma of the cervix in women from Okinawa, Japan. Am J Clin Oncol 2001;24(2):160-6.

Nagai Y, Maehama T, Asato T, Kanazawa K. Persistence of human papillomavirus infection after therapeutic conization for CIN 3: is it an alarm for disease recurrence? Gynecol Oncol 2000;79(2):294-9.

Ngamkham J, Homcha-Aim P, Boonmark K, Phansri T, Swangvaree SS. Preliminary Study on Human Papillomavirus Frequency and Specific Type-distribution in Vulva Cancer from Thai Women. Asian Pac J Cancer Prev 2013;14(4):2355-9.

Nishiwaki M, Yamamoto T, Tone S, Murai T, Ohkawara T, Matsunami T, et al. Genotyping of human papillomaviruses by a novel one-step typing method with multiplex PCR and clinical applications. J Clin Microbiol 2008;46(4):1161-8.

Oh YL, Shin KJ, Han J, Kim DS. Significance of high-risk human papillomavirus detection by polymerase chain reaction in primary cervical cancer screening. Cytopathology 2001;12:75-83.

Oh JK, Ju YH, Franceschi S, Quint W, Shin HR. Acquisition of new infection and clearance of type-specific human papillomavirus infections in female students in Busan, South Korea: a follow-up study. BMC Infect Dis 2008;8:13.

Oh JK, Franceschi S, Kim BK, Kim JY, Ju YH, Hong EK, et al. Prevalence of human papillomavirus and Chlamydia trachomatis infection among women attending cervical cancer screening in the Republic of Korea. Eur J Cancer Prev 2009;18(1):56-61.

Oh JK, Alemany L, Suh JI, Rha SH, Munoz N, Bosch FX, et al. Type-specific human papillomavirus distribution in invasive cervical cancer in Korea, 1958-2004. Asian Pac J Cancer Prev 2010;11(4):993-1000.

Okadome M, Saito T, Tanaka H, Nogawa T, Furuta R, Watanabe K, et al. Potential impact of combined high- and low-risk human papillomavirus infection on the progression of cervical intraepithelial neoplasia 2. J Obstet Gynaecol Res 2014;40(2):561-9.

Onuki M, Matsumoto K, Satoh T, Oki A, Okada S, Minaguchi T, et al. Human papillomavirus infections among Japanese women: age-related prevalence and type-specific risk for cervical cancer. Cancer Sci 2009;100(7):1312-6.

Osakabe M, Hayashi M, Katayama Y, Emura I, Nemoto K, Umezu H, et al. Characteristics of vulvar squamous cell carcinoma in Japanese women. Pathol Int 2007;57(6):322-7.

Othman N, Othman NH. Detection of human papillomavirus DNA in routine cervical scraping samples: use for a national cervical cancer screening program in a developing nation. Asian Pac J Cancer Prev 2014;15(5):2245-9.

Ozalp SS, Us T, Arslan E, Oge T, Kasifoglu N. HPV DNA and Pap smear test results in cases with and without cervical pathology. J Turkish-German Gynecol Assoc 2012;13(1):8-14.

Park EK, Cho H, Lee SH, Lee SG, Lee SY, Kim KH, et al. Human Papillomavirus Prevalence and Genotype Distribution among HIV-Infected Women in Korea. J Korean Med Sci 2014;29(1):32-7.

Park JS, Kim YT, Lee A, Lee Y, Kim KT, Cho CH, et al. Prevalence and type distribution of human papillomavirus in cervical adenocarcinoma in Korean women. Gynecol Oncol 2013;130(1):115-20.

Park TC, Kim CJ, Koh YM, Lee KH, Yoon JH, Kim JH, et al. Human Papillomavirus Genotyping by the DNA Chip in the Cervical Neoplasia. DNA and Cell Biology 2004;23(2):119-25.

Peedicayil A, Abraham P, Sathish N, John S, Shah K, Sridharan G, et al. Human papillomavirus genotypes associated with cervical neoplasia in India. Int J Gynecol Cancer 2006;16(4):1591-5.

Pham TH, Nguyen TH, Herrero R, Vaccarella S, Smith JS, Nguyen Thuy TT, et al. Human papillomavirus infection among women in South and North Vietnam. Int J Cancer 2003;104(2):213-20.

Phongsavan K, Gustavsson I, Marions L, Phengsavanh A, Wahlstrom R, Gyllensten U. Detection of human papillomavirus among women in Laos: feasibility of using filter paper card and prevalence of high-risk types. Int J Gynecol Cancer 2012;22(8):1398-406.

Pillai RM, Babu JM, Jissa VT, Lakshmi S, Chiplunkar SV, Patkar M, et al. Region-wise distribution of high-risk human papillomavirus types in squamous cell carcinomas of the cervix in India. Int J Gynecol Cancer 2010;20(6):1046-51.

Qiu AD, Wu EQ, Yu XH, Jiang CL, Jin YH, Wu YG, et al. HPV prevalence, E6 sequence variation and physical state of HPV16 isolates from patients with cervical cancer in Sichuan, China. Gynecol Oncol 2007;104(1):77-85.

Quek SC, Lim BK, Domingo E, Soon R, Park JS, Vu TN, et al. Human papillomavirus type distribution in invasive cervical cancer and high-grade cervical intraepithelial neoplasia across 5 countries in Asia. Int J Gynecol Cancer 2013;23(1):148-56.

Rachmadi L, Jordanova ES, Kolkman-Uljee S, van dL-N, I, Purwoto G, Siregar B, et al. Cytomorphological analysis of uterine cervical pap smears in relation to human papillomavirus infection in indonesian women. Acta Cytol 2012;56(2):171-6.

Raza SA, Franceschi S, Pallardy S, Malik FR, Avan BI, Zafar A, et al. Human papillomavirus infection in women with and without cervical cancer in Karachi, Pakistan. Br J Cancer 2010;102(11):1657-60.

Rumbold AR, Tan SE, Condon JR, Taylor-Thomson D, Nickels M, Tabrizi SN, et al. Investigating a cluster of vulvar cancer in young women: a cross-sectional study of genital human papillomavirus prevalence. BMC Infect Dis 2012;12:243.

Sahiner F, Kubar A, Yapar M, Sener K, Dede M, Gumral R. Detection of major HPVs by a new multiplex real-time PCR assay using type-specific primers. J Microbiol Methods 2014;97:44-50.

Saito J, Hoshiai H, Noda K. Type of human papillomavirus and expression of p53 in elderly women with cervical cancer. Gynecol Obstet Invest 2000;49(3):190-3.

Samarawickrema NA, Tabrizi SN, Hewavisenthi J, Leong T, Garland SM. Distribution of human papillomavirus genotypes in archival cervical tissue from women with cervical cancer in urban Sri Lanka. Int J Gynaecol Obstet 2011;115(2):180-2.

Sasagawa T, Basha W, Yamazaki H, Inoue M. High-risk and multiple human papillomavirus infections associated with cervical abnormalities in Japanese women. Cancer Epidemiol Biomarkers Prev 2001;10(1):45-52.

Satoh T, Matsumoto K, Fujii T, Sato O, Gemma N, Onuki M, et al. Rapid genotyping of carcinogenic human papillomavirus by loop-mediated isothermal amplification using a new automated DNA test (Clinichip HPV). J Virol Methods 2013;188(1-2):83-93.

Sayyed DR, Song KS, Nimse SB, An H, Kim J, Kim T. HPV genotyping 9G membrane test. Viruses 2013;5(11):2840-55.

Schellekens MC, Dijkman A, Aziz MF, Siregar B, Cornain S, Kolkman-Uljee S, et al. Prevalence of single and multiple HPV types in cervical carcinomas in Jakarta, Indonesia. Gynecol Oncol 2004;93(1):49-53.

Settheetham-Ishida W, Kanjanavirojkul N, Kularbkaew C, Ishida T. Human papillomavirus genotypes and the p53 codon 72 polymorphism in cervical cancer of Northeastern Thailand. Microbiol Immunol 2005;49(5):417-21.

Settheetham-Ishida W, Yuenyao P, Tassaneeyakul W, Kanjanavirojkul N, Thawmor A, Kularbkaew C, et al. Selected risk factors, human papillomavirus infection and the p53 codon 72 polymorphism in patients with squamous intraepithelial lesions in northeastern Thailand. Asian Pac J Cancer Prev 2006;7(1):113-8.

Shah W, Hongwei C, Jin Z, Lifang D, Jun Y, Yili W. The prevalence of human papillomavirus type 58 in Chinese patients with cervical carcinoma and its influence on survival. Clin Oncol (R Coll Radiol) 2009;21(10):768-74.

Shahsiah R, Khademalhosseini M, Mehrdad N, Ramezani F, Nadji SA. Human papillomavirus genotypes in Iranian patients with cervical cancer. Pathol Res Pract 2011;207(12):754-7.

Shen Y, Gong JM, Li YQ, Gong YM, Lei DM, Cheng GM, et al. Epidemiology and genotype distribution of human papillomavirus (HPV) in women of Henan Province, China. Clin Chim Acta 2013;415:297-301.

Sherpa AT, Clifford GM, Vaccarella S, Shrestha S, Nygard M, Karki BS, et al. Human papillomavirus infection in women with and without cervical cancer in Nepal. Cancer Causes Control 2010;21(3):323-30.

Shin HR, Lee DH, Herrero R, Smith JS, Vaccarella S, Hong SH, et al. Prevalence of human papillomavirus infection in women in Busan, South Korea. Int J Cancer 2003;103(3):413-21.

Shin HR, Franceschi S, Vaccarella S, Roh JW, Ju YH, Oh JK, et al. Prevalence and determinants of genital infection with papillomavirus, in female and male university students in Busan, South Korea. J Infect Dis 2004;190(3):468-76.

Simonella LM, Lewis H, Smith M, Neal H, Bromhead C, Canfell K. Type-specific oncogenic human papillomavirus infection in high grade cervical disease in New Zealand. BMC Infect Dis 2013;13:114.

Singh A, Datta P, Jain SK, Bhatla N, Dutta GS, Dey B, et al. Human papilloma virus genotyping, variants and viral load in tumors, squamous intraepithelial lesions, and controls in a north Indian population subset. Int J Gynecol Cancer 2009;19(9):1642-8.

Siriaunkgul S, Suwiwat S, Settakorn J, Khunamornpong S, Tungsinmunkong K, Boonthum A, et al. HPV genotyping in cervical cancer in Northern Thailand: adapting the linear array HPV assay for use on paraffin-embedded tissue. Gynecol Oncol 2008;108(3):555-60.

Siriaunkgul S, Utaipat U, Settakorn J, Sukpan K, Srisomboon J, Khunamornpong S. HPV genotyping in neuroendocrine carcinoma of the uterine cervix in northern Thailand. Int J Gynaecol Obstet 2011;115(2):175-9.

Siriaunkgul S, Utaipat U, Suthipintawong C, Tungsinmunkong K, Triratanachat S, Khunamornpong S. HPV genotyping in adenocarcinoma of the uterine cervix in Thailand. Int J Gynaecol Obstet 2013;123(3):226-30.

Sowjanya AP, Jain M, Poli UR, Padma S, Das M, Shah KV, et al. Prevalence and distribution of high-risk human papilloma virus (HPV) types in invasive squamous cell carcinoma of the cervix and in normal women in Andhra Pradesh, India. BMC Infect Dis 2005;5:116.

Srivastava S, Gupta S, Roy JK. High prevalence of oncogenic HPV-16 in cervical smears of asymptomatic women of eastern Uttar Pradesh, India: a population-based study. J Biosci 2012;37(1):63-72.

Stevens MP, Tabrizi SN, Quinn MA, Garland SM. Human papillomavirus genotype prevalence in cervical biopsies from women diagnosed with cervical intraepithelial neoplasia or cervical cancer in Melbourne, Australia. Int J Gynecol Cancer 2006;16(3):1017-24.

Stevens MP, Garland SM, Tan JH, Quinn MA, Petersen RW, Tabrizi SN. HPV genotype prevalence in women with abnormal pap smears in Melbourne, Australia. J Med Virol 2009;81(7):1283-91.

Sukasem C, Pairoj W, Saekang N, Pombubpha H, Srichunrasami C, Pongtippan A, et al. Molecular epidemiology of human papillomavirus genotype in women with high-grade squamous intraepithelial lesion and cervical cancer: will a quadrivalent vaccine be necessary in Thailand? J Med Virol 2011;83(1):119-26.

Sukvirach S, Smith JS, Tunsakul S, Munoz N, Kesararat V, Opasatian O, et al. Population-based human papillomavirus prevalence in Lampang and Songkla, Thailand. J Infect Dis 2003;187(8):1246-56.

Sun B, He J, Chen X, He M, He Z, Wang Y, et al. Prevalence and genotype distribution of human papillomavirus infection in Harbin, Northeast China. Arch Virol 2014;159(5):1027-32.

Sun LL, Jin Q, Li H, Zhou XR, Song ZQ, Cheng XM, et al. Population-based study on the prevalence of and risk factors for human papillomavirus infection in Qujing of Yunnan province, Southwest China. Virol J 2012;9:153.

Suthipintawong C, Siriaunkgul S, Tungsinmunkong K, Pientong C, Ekalaksananan T, Karalak A, et al. Human papilloma virus prevalence, genotype distribution, and pattern of infection in Thai women. Asian Pac J Cancer Prev 2011;12(4):853-6.

Suwannarurk K, Tapanadechopol P, Pattaraarchachai J, Bhamarapravati S. Hospital-based prevalence and sensitivity of high-risk human papillomavirus in Thai urban population. Cancer Epidemiol 2009;33(1):56-60.

Swangvaree SS, Kongkaew P, Ngamkham J. Frequency and Type-distribution of Human Papillomavirus from Paraffin-embedded Blocks of High Grade Cervical Intraepithelial Neoplasia Lesions in Thailand. Asian Pac J Cancer Prev 2013;14(2):1023-6.

Tabone T, Garland SM, Mola G, O'Connor M, Danielewski J, Tabrizi SN. Prevalence of human papillomavirus genotypes in women with cervical cancer in Papua New Guinea. Int J Gynaecol Obstet 2012;117(1):30-2.

Tabrizi SN, Law I, Buadromo E, Stevens MP, Fong J, Samuela J, et al. Human papillomavirus genotype prevalence in cervical biopsies from women diagnosed with cervical intraepithelial neoplasia or cervical cancer in Fiji. Sex Health 2011;8(3):338-42.

Takehara K, Toda T, Nishimura T, Sakane J, Kawakami Y, Mizunoe T, et al. Human papillomavirus types 52 and 58 are prevalent in uterine cervical squamous lesions from Japanese women. Patholog Res Int 2011;2011:246936.

Tan SE, Garland SM, Rumbold AR, Zardawi I, Taylor-Thomson D, Condon JR, et al. Investigating a cluster of vulvar cancers in young women: distribution of human papillomavirus and HPV-16 variants in vulvar dysplastic or neoplastic biopsies. Sex Health 2013;10(1):18-25.

Tay SK, Oon LL. Prevalence of cervical human papillomvirus infection in healthy women is related to sexual behaviours and educational level: a cross-sectional study. Int J STD AIDS 2014;25(14):1013-21.

Thomas DB, Ray RM, Koetsawang A, Kiviat N, Kuypers J, Qin Q, et al. Human papillomaviruses and cervical cancer in Bangkok. I. Risk factors for invasive cervical carcinomas with human papillomavirus types 16 and 18 DNA. Am J Epidemiol 2001;153(8):723-31.

Tong SY, Lee YS, Park JS, Namkoong SE. Human papillomavirus genotype as a prognostic factor in carcinoma of the uterine cervix. Int J Gynecol Cancer 2007;17(6):1307-13.

Tsao KC, Huang CG, Kuo YB, Chang TC, Sun CF, Chang CA, et al. Prevalence of human papillomavirus genotypes in northern Taiwanese women. J Med Virol 2010;82(10):1739-45.

Tsuda H, Hashiguchi Y, Nishimura S, Kawamura N, Inoue T, Yamamoto K. Relationship between HPV typing and abnormality of G1 cell cycle regulators in cervical neoplasm. Gynecol Oncol 2003;91(3):476-85.

Twu NF, Yen MS, Lau HY, Chen YJ, Yu BK, Lin CY. Type-specific human papillomavirus DNA testing with the genotyping array: a comparison of cervical and vaginal sampling. Eur J Obstet Gynecol Reprod Biol 2011;156(1):96-100.

Usubutun A, Alemany L, Kucukali T, Ayhan A, Yuce K, de Sanjose S, et al. Human papillomavirus types in invasive cervical cancer specimens from Turkey. Int J Gynecol Pathol 2009;28(6):541-8.

Vet JN, de Boer MA, van den Akker BE, Siregar B, Lisnawati, Budiningsih S, et al. Prevalence of human papillomavirus in Indonesia: a population-based study in three regions. Br J Cancer 2008;99(1):214-8.

Vu L, Le H, Luong O, Tran H, Nguyen N, Luu H. Prevalence of cervical human papillomavirus infection among married women in hanoi, Vietnam, 2010. Asia Pac J Public Health 2012;24(2):385-90.

Vu LT, Le HT. Cervical human papilloma virus infection among the general female population in Vietnam: a situation analysis. Asian Pac J Cancer Prev 2011;12(2):561-6.

Vu LT, Bui D, Le HT. Prevalence of cervical infection with HPV type 16 and 18 in Vietnam: implications for vaccine campaign. BMC Cancer 2013;13:53.

Wang CH, Garvilles RG, Chen CY. Characterization of human papillomavirus infection in north Taiwan. J Med Virol 2010;82(8):1416-23.

Watari H, Michimata R, Yasuda M, Ishizu A, Tomaru U, Xiong Y, et al. High prevalence of multiple human papillomavirus infection in Japanese patients with invasive uterine cervical cancer. Pathobiology 2011;78(4):220-6.

Wentzensen N, Wilson LE, Wheeler CM, Carreon JD, Gravitt PE, Schiffman M, et al. Hierarchical clustering of human papilloma virus genotype patterns in the ASCUS-LSIL triage study. Cancer Res 2010;70(21):8578-86.

Williamson D, Nagappan R, Sirikonda R, Rahnama F, Thomas S, Lovell-Smith M, et al. Distribution of HPV genotypes in women with cervical cancer in Auckland, New Zealand; a review of 50 specimens between 2000-2006. Aust N Z J Obstet Gynaecol 2011;51(1):67-70.

Wu D, Cai L, Huang M, Zheng Y, Yu J. Prevalence of genital human papillomavirus infection and genotypes among women from Fujian province, PR China. Eur J Obstet Gynecol Reprod Biol 2010;151(1):86-90.

Wu D, Zheng Y, Chen W, Guo C, Yu J, Chen G, et al. Prediction of residual/recurrent disease by HPV genotype after loop excision procedure for high-grade cervical intraepithelial neoplasia with negative margins. Aust N Z J Obstet Gynaecol 2011;51(2):114-8.

Wu EQ, Zhang GN, Yu XH, Ren Y, Fan Y, Wu YG, et al. Evaluation of high-risk human papillomaviruses type distribution in cervical cancer in Sichuan province of China. BMC Cancer 2008;8:202.

Wu EQ, Yu XH, Zha X, Zhang GN, Wang JH, Fan Y, et al. Distribution of human papillomavirus genotypes in archival cervical lesions in eastern inner Mongolian autonomous region, China. Int J Gynecol Cancer 2009;19(5):919-23.

Wu EQ, Liu B, Cui JF, Chen W, Wang JB, Lu L, et al. Prevalence of type-specific human papillomavirus and pap results in Chinese women: a multi-center, population-based cross-sectional study. Cancer Causes Control 2013;24(4):795-803.

Wu RF, Dai M, Qiao YL, Clifford GM, Liu ZH, Arslan A, et al. Human papillomavirus infection in women in Shenzhen City, People's Republic of China, a population typical of recent Chinese urbanisation. Int J Cancer 2007;121(6):1306-11.

Wu X, Zhang C, Feng S, Liu C, Li Y, Yang Y, et al. Detection of HPV types and neutralizing antibodies in Gansu province, China. J Med Virol 2009;81(4):693-702.

Wu Y, Zhang Q, Liu B, Yu G. The analysis of the entire HLA, partial non-HLA and HPV for Chinese women with cervical cancer. J Med Virol 2008;80(10):1808-13.

Ye J, Cheng X, Chen X, Ye F, Lu W, Xie X. Prevalence and risk profile of cervical Human papillomavirus infection in Zhejiang Province, southeast China: a population-based study. Virol J 2010;7:66.

Yip YC, Ngai KL, Vong HT, Tzang LC, Ji S, Yang M, et al. Prevalence and genotype distribution of cervical human papillomavirus infection in Macao. J Med Virol 2010;82(10):1724-9.

Yousefzadeh A, Mostafavizadeh SM, Jarollahi A, Raeisi M, Garshasbi M, Siavashvahabi Z, et al. Human papillomavirus (HPV) prevalence and types among women attending regular gynecological visit in Tehran, Iran. Clin Lab 2014;60(2):267-73.

Yousuf S, Syed S, Moazzam A, Lucky MH. Frequency of high risk human papillomavirus types in squamous cell carcinoma of cervix among women. J Pak Med Assoc 2010;60(3):193-6.

Yu XW, Zhang XW, Wang L, Li F, Xu J. Status of human papillomavirus infection in the rural female population in Northwestern China: an observational study. J Low Genit Tract Dis 2013;17(1):17-22.

Yuan X, Yang Y, Gu D, Liu H, Yang H, Wang M. Prevalence of human papillomavirus infection among women with and without normal cervical histology in Shandong Province, China. Arch Gynecol Obstet 2011;283(6):1385-9.

Yuce K, Pinar A, Salman MC, Alp A, Sayal B, Dogan S, et al. Detection and genotyping of cervical HPV with simultaneous cervical cytology in Turkish women: a hospital-based study. Arch Gynecol Obstet 2012;286(1):203-8.

Zandi K, Eghbali SS, Hamkar R, Ahmadi S, Ramedani E, Deilami I, et al. Prevalence of various human papillomavirus (HPV) genotypes among women who subjected to routine Pap smear test in Bushehr city (south west of Iran) 2008-2009. Virol J 2010;7:65.

Zhang L, Wang Y, Peng M, She Q, Xiang Q, Chen Q, et al. Prevalence and type distribution of high-risk human papillomavirus infections among women in Wufeng County, China. Arch Gynecol Obstet 2012;286(3):695-9.

Zhang R, Shi TY, Ren Y, Lu H, Wei ZH, Hou WJ, et al. Risk factors for human papillomavirus infection in Shanghai suburbs: a population-based study with 10,000 women. J Clin Virol 2013;58(1):144-8.

Zhang R, Velicer C, Chen W, Liaw KL, Wu EQ, Liu B, et al. Human papillomavirus genotype distribution in cervical intraepithelial neoplasia grades 1 or worse among 4215 Chinese women in a population-based study. Cancer Epidemiol 2013;37(6):939-45.

Zhao FH, Jeronimo J, Qiao YL, Schweizer J, Chen W, Valdez M, et al. An evaluation of novel, lower-cost molecular screening tests for human papillomavirus in rural China. Cancer Prev Res (Phila) 2013;6(9):938-48.

Zhao FH, Zhu FC, Chen W, Li J, Hu YM, Hong Y, et al. Baseline prevalence and type distribution of human papillomavirus in healthy Chinese women aged 18-25 years enrolled in a clinical trial. Int J Cancer 2014;135(11):2604-11.

Zhao R, Zhang WY, Wu MH, Zhang SW, Pan J, Zhu L, et al. Human papillomavirus infection in Beijing, People's Republic of China: a population-based study. Br J Cancer 2009;101(9):1635-40.

Zhao Y, Lin H, Shen D, Xuan Y, Lin Z. Distribution of HPV genotypes in uterine cervical lesions in Yanbian, northern China. Pathol Int 2008;58(10):643-7.

1.3 Europe

Agarossi A, Ferrazzi E, Parazzini F, Perno CF, Ghisoni L. Prevalence and type distribution of high-risk human papillomavirus infection in women undergoing voluntary cervical cancer screening in Italy. J Med Virol 2009;81(3):529-35.

Agodi A, Barchitta M, La Rosa N, Cipresso R, Guarnaccia M, Caruso M, et al. Human papillomavirus infection: low-risk and high-risk genotypes in women in Catania, Sicily. Int J Gynecol Cancer 2009;19(6):1094-8.

Agorastos T, Lambropoulos AF, Sotiriadis A, Mikos T, Togaridou E, Emmanouilides CJ. Prevalence and distribution of high-risk human papillomavirus in Greece. Eur J Cancer Prev 2009;18(6):504-9.

Alemany L, Perez C, Tous S, Llombart-Bosch A, Lloveras B, Lerma E, et al. Human papillomavirus genotype distribution in cervical cancer cases in Spain. Implications for prevention. Gynecol Oncol 2012;124(3):512-7.

Alonso I, Felix A, Torne A, Fuste V, Del PM, Castillo P, et al. Human papillomavirus as a favorable prognostic biomarker in squamous cell carcinomas of the vagina. Gynecol Oncol 2012;125(1):194-9.

Ambrosio MR, Onorati M, Rocca BJ, Santopietro R. Vulvar cancer and HPV infection: analysis of 22 cases. Pathologica 2008;100(5):405-7.

Ammatuna P, Giovannelli L, Matranga D, Ciriminna S, Perino A. Prevalence of genital human papilloma virus infection and genotypes among young women in Sicily, South Italy. Cancer Epidemiol Biomarkers Prev 2008;17(8):2002-6.

Anderson L, O'Rorke M, Jamison J, Wilson R, Gavin A. Prevalence of human papillomavirus in women attending cervical screening in the UK and Ireland: new data from northern Ireland and a systematic review and meta-analysis. J Med Virol 2013;85(2):295-308.

Andersson S, Larson B, Hjerpe A, Silfversward C, Sallstrom J, Wilander E, et al. Adenocarcinoma of the uterine cervix: the presence of human papillomavirus and the method of detection. Acta Obstet Gynecol Scand 2003;82(10):960-5.

Andersson S, Rylander E, Larson B, Sigurdardottir S, Backlund I, Sallstrom J, et al. Types of human papillomavirus revealed in cervical adenocarcinomas after DNA sequencing. Oncol Rep 2003;10(1):175-9.

Andersson S, Mints M, Sallstrom J, Wilander E. The relative distribution of oncogenic types of human papillomavirus in benign, pre-malignant and malignant cervical biopsies. A study with human papillomavirus deoxyribonucleic acid sequence analysis. Cancer Detect Prev 2005;29(1):37-41.

Andersson S, Safari H, Mints M, Lewensohn-Fuchs I, Gyllensten U, Johansson B. Type distribution, viral load and integration status of high-risk human papillomaviruses in pre-stages of cervical cancer (CIN). Br J Cancer 2005;92(12):2195-200.

Anton G, Peltecu G, Socolov D, Cornitescu F, Bleotu C, Sgarbura Z, et al. Type-specific human papillomavirus detection in cervical smears in Romania. APMIS 2011;119(1):1-9.

Arbyn M, Benoy I, Simoens C, Bogers J, Beutels P, Depuydt C. Prevaccination distribution of human papillomavirus types in women attending at cervical cancer screening in Belgium. Cancer Epidemiol Biomarkers Prev 2009;18(1):321-30.

Argyri E, Tsimplaki E, Daskalopoulou D, Stravopodis DJ, Kouikoglou O, Terzakis E, et al. E6/E7 mRNA expression of high-risk HPV types in 849 Greek women. Anticancer Res 2013;33(9):4007-11.

Baalbergen A, Smedts F, Ewing P, Snijders PJ, Meijer CJ, Helmerhorst TJ. HPV-type has no impact on survival of patients with adenocarcinoma of the uterine cervix. Gynecol Oncol 2013;128(3):530-4.

Baandrup L, Munk C, Andersen KK, Junge J, Iftner T, Kjaer SK. HPV16 is associated with younger age in women with cervical intraepithelial neoplasia grade 2 and 3. Gynecol Oncol 2012;124(2):281-5.

Baay MF, Tjalma WA, Weyler J, Goovaerts G, Buytaert P, Van Marck EA, et al. Human papillomavirus infection in the female population of Antwerp, Belgium: prevalence in healthy women, women with premalignant lesions and cervical cancer. Eur J Gynaecol Oncol 2001;22(3):204-8.

Baay MF, Tjalma WA, Lambrechts HA, Pattyn GG, Lardon F, Weyler J, et al. Combined Pap and HPV testing in primary screening for cervical abnormalities: should HPV detection be delayed until age 35? Eur J Cancer 2005;41(17):2704-8.

Bachtiary B, Obermair A, Dreier B, Birner P, Breitenecker G, Knocke TH, et al. Impact of multiple HPV infection on response to treatment and survival in patients receiving radical radiotherapy for cervical cancer. Int J Cancer 2002;102(3):237-43.

Bardin A, Vaccarella S, Clifford GM, Lissowska J, Rekosz M, Bobkiewicz P, et al. Human papillomavirus infection in women with and without cervical cancer in Warsaw, Poland. Eur J Cancer 2008;44(4):557-64.

Barzon L, Militello V, Pagni S, Franchin E, Dal Bello F, Mengoli C, et al. Distribution of human papillomavirus types in the anogenital tract of females and males. J Med Virol 2010;82(8):1424-30.

Barzon L, Militello V, Pagni S, Palu G. Comparison of INNO-LiPA genotyping extra and hybrid capture 2 assays for detection of carcinogenic human papillomavirus genotypes. J Clin Virol 2012;55(3):256-61.

Baudu A, Pretet J-L, Riethmuller D, Chotard M, Mougin C, Mercier M. Prevalence and risk factors of human papillomavirus infection types 16/18/45 in a cohort of French females aged 15-23years. J Epidemiol Glob Health 2014;4(1):35-43.

Beby-Defaux A, Bourgoin A, Ragot S, Battandier D, Lemasson JM, Renaud O, et al. Human papillomavirus infection of the cervix uteri in women attending a Health Examination Center of the French social security. J Med Virol 2004;73(2):262-8.

Bekkers RL, Bulten J, Wiersma-van TA, Mravunac M, Schijf CP, Massuger LF, et al. Coexisting high-grade glandular and squamous cervical lesions and human papillomavirus infections. Br J Cancer 2003;89(5):886-90.

Bello BD, Spinillo A, Alberizzi P, Cesari S, Gardella B, D'Ambrosio G, et al. Cervical infections by multiple human papillomavirus (HPV) genotypes: Prevalence and impact on the risk of precancerous epithelial lesions. J Med Virol 2009;81(4):703-12.

Bernal M, Burillo I, Mayordomo JI, Moros M, Benito R, Gil J. Human papillomavirus (HPV) infection and intraepithelial neoplasia and invasive cancer of the uterine cervix: A case-control study in Zaragoza, Spain. Infectious Agents and Cancer 2008;3:8.

Bertelsen BI, Kugarajh K, Skar R, Laerum OD. HPV subtypes in cervical cancer biopsies between 1930 and 2004: detection using general primer pair PCR and sequencing. Virchows Arch 2006;449(2):141-7.

Birner P, Bachtiary B, Dreier B, Schindl M, Joura EA, Breitenecker G, et al. Signal-amplified colorimetric in situ hybridization for assessment of human papillomavirus infection in cervical lesions. Mod Pathol 2001;14(7):702-9.

Bonvicini F, Venturoli S, Ambretti S, Paterini P, Santini D, Ceccarelli C, et al. Presence and type of oncogenic human papillomavirus in classic and in differentiated vulvar intraepithelial neoplasia and keratinizing vulvar squamous cell carcinoma. J Med Virol 2005;77(1):102-6.

Brentnall AR, Vasiljevic N, Scibior-Bentkowska D, Cadman L, Austin J, Szarewski A, et al. A DNA methylation classifier of cervical precancer based on human papillomavirus and human genes. Int J Cancer 2014;135(6):1425-32.

Broccolo F, Chiari S, Piana A, Castiglia P, Dell'Anna T, Garcia-Parra R, et al. Prevalence and viral load of oncogenic human papillomavirus types associated with cervical carcinoma in a population of North Italy. J Med Virol 2009;81(2):278-87.

Broccolo F, Fusetti L, Rosini S, Caraceni D, Zappacosta R, Ciccocioppo L, et al. Comparison of oncogenic HPV type-specific viral DNA load and E6/E7 mRNA detection in cervical samples: results from a multicenter study. J Med Virol 2013;85(3):472-82.

Bryant D, Rai N, Rowlands G, Hibbitts S, Jones J, Tristram A, et al. Human papillomavirus type distribution in vulval intraepithelial neoplasia determined using PapilloCheck DNA Microarray. J Med Virol 2011;83(8):1358-61.

Bulk S, Berkhof J, Bulkmans NW, Zielinski GD, Rozendaal L, van Kemenade FJ, et al. Preferential risk of HPV16 for squamous cell carcinoma and of HPV18 for adenocarcinoma of the cervix compared to women with normal cytology in The Netherlands. Br J Cancer 2006;94(1):171-5.

Bulk S, Berkhof J, Rozendaal L, Fransen Daalmeijer NC, Gok M, de Schipper FA, et al. The contribution of HPV18 to cervical cancer is underestimated using high-grade CIN as a measure of screening efficiency. Br J Cancer 2007;96(8):1234-6.

Bulkmans NW, Bleeker MC, Berkhof J, Voorhorst FJ, Snijders PJ, Meijer CJ. Prevalence of types 16 and 33 is increased in high-risk human papillomavirus positive women with cervical intraepithelial neoplasia grade 2 or worse. Int J Cancer 2005;117(2):177-81.

Carozzi F, De ML, Gillio-Tos A, Del MA, Girlando S, Baboci L, et al. Age and geographic variability of human papillomavirus high-risk genotype distribution in a large unvaccinated population and of vaccination impact on HPV prevalence. J Clin Virol 2014;60(3):257-63.

Carozzi FM, Confortini M, Cecchini S, Bisanzi S, Cariaggi MP, Pontenani G, et al. Triage with human papillomavirus testing of women with cytologic abnormalities prompting referral for colposcopy assessment. Cancer 2005;105(1):2-7.

Carozzi FM, Tornesello ML, Burroni E, Loquercio G, Carillo G, Angeloni C, et al. Prevalence of human papillomavirus types in high-grade cervical intraepithelial neoplasia and cancer in Italy. Cancer Epidemiol Biomarkers Prev 2010;19(9):2389-400.

Casalegno JS, Benchaib M, Le Bail CK, Piaton E, Mathevet P, Mekki Y. Human papillomavirus genotype distribution among French women with and without cervical abnormalities. Int J Gynaecol Obstet 2011;114(2):116-9.

Castellsague X, Iftner T, Roura E, Vidart JA, Kjaer SK, Bosch FX, et al. Prevalence and genotype distribution of human papillomavirus infection of the cervix in Spain: the CLEOPATRE study. J Med Virol 2012;84(6):947-56.

Centurioni MG, Puppo A, Merlo DF, Pasciucco G, Cusimano ER, Sirito R, et al. Prevalence of human papillomavirus cervical infection in an Italian asymptomatic population. BMC Infect Dis 2005;5:77.

Cercato MC, Mariani L, Vocaturo A, Carrone A, Terrenato I, Morano G, et al. Predictors of human papilloma virus (HPV) infection in Italian women. J Med Virol 2010;82(11):1921-7.

Chironna M, Neve A, Sallustio A, De RA, Quarto M, Germinario C, et al. Frequency of human papillomavirus infection and genotype distribution among women with known cytological diagnosis in a Southern Italian region. J Prev Med Hyg 2010;51(4):139-45.

Ciotti M, Paba P, Bonifacio D, Di Bonito L, Benedetto A, Favalli C. Single or multiple HPV types in cervical cancer and associated metastases. Oncol Rep 2006;15(1):143-8.

Conesa-Zamora P, Ortiz-Reina S, Moya-Biosca J, Domenech-Peris A, Orantes-Casado FJ, Perez-Guillermo M, et al. Genotype distribution of human papillomavirus (HPV) and co-infections in cervical cytologic specimens from two outpatient gynecological clinics in a region of southeast Spain. BMC Infect Dis 2009;9:124.

Confortini M, Carozzi F, Zappa M, Ventura L, Iossa A, Cariaggi P, et al. Human papillomavirus infection and risk factors in a cohort of Tuscan women aged 18-24: results at recruitment. BMC Infect Dis 2010;10:157-67.

Costa S, Venturoli S, Mennini FS, Marcellusi A, Pesaresi M, Leo E, et al. Population-based frequency assessment of HPV-induced lesions in patients with borderline Pap tests in the Emilia-Romagna Region: the PATER study. Curr Med Res Opin 2011;27(3):569-78.

Coupe VM, Berkhof J, Bulkmans NW, Snijders PJ, Meijer CJ. Age-dependent prevalence of 14 high-risk HPV types in the Netherlands: implications for prophylactic vaccination and screening. Br J Cancer 2008;98(3):646-51.

Cuschieri KS, Cubie HA, Whitley MW, Seagar AL, Arends MJ, Moore C, et al. Multiple high risk HPV infections are common in cervical neoplasia and young women in a cervical screening population. J Clin Pathol 2004;57(1):68-72.

Cuschieri K, Brewster DH, Williams AR, Millan D, Murray G, Nicoll S, et al. Distribution of HPV types associated with cervical cancers in Scotland and implications for the impact of HPV vaccines. Br J Cancer 2010;102(5):930-2.

Cuschieri K, Kavanagh K, Sinka K, Robertson C, Cubie H, Moore C, et al. Effect of HPV assay choice on perceived prevalence in a population-based sample. Diagn Mol Pathol 2013;22(2):85-90.

Cuzick J, Terry G, Ho L, Monaghan J, Lopes A, Clarkson P, et al. Association between high-risk HPV types, HLA DRB1* and DQB1* alleles and cervical cancer in British women. Br J Cancer 2000;82(7):1348-52.

Dabic MM, Hlupic L, Babic D, Jukic S, Seiwerth S. Comparison of polymerase chain reaction and catalyzed signal amplification in situ hybridization methods for human papillomavirus detection in paraffin-embedded cervical preneoplastic and neoplastic lesions. Arch Med Res 2004;35(6):511-6.

Dabic MM, Nola M, Tomicic I, Dotlic S, Petrovecki M, Jukic S. Adenocarcinoma of the uterine cervix: prognostic significance of clinicopathologic parameters, flow cytometry analysis and HPV infection. Acta Obstet Gynecol Scand 2008;87(3):366-72.

Dahlstrom LA, Ylitalo N, Sundstrom K, Palmgren J, Ploner A, Eloranta S, et al. Prospective study of human papillomavirus and risk of cervical adenocarcinoma. Int J Cancer 2010;127(8):1923-30.

Darlin L, Borgfeldt C, Forslund O, Henic E, Hortlund M, Dillner J, et al. Comparison of use of vaginal HPV self-sampling and offering flexible appointments as strategies to reach long-term non-attending women in organized cervical screening. J Clin Virol 2013;58(1):155-60.

de Bie RP, van de Nieuwenhof HP, Bekkers RL, Melchers WJ, Siebers AG, Bulten J, et al. Patients with usual vulvar intraepithelial neoplasia-related vulvar cancer have an increased risk of cervical abnormalities. Br J Cancer 2009;101(1):27-31.

De Francesco MA, Gargiulo F, Schreiber C, Ciravolo G, Salinaro F, Manca N. Detection and genotyping of human papillomavirus in cervical samples from Italian patients. J Med Virol 2005;75(4):588-92.

De Francesco MA, Gargiulo F, Schreiber C, Ciravolo G, Salinaro F, Manca N. Prevaccination distribution of human papillomavirus types in Italian women with high-risk lesions and cervical neoplasia. Intervirology 2010;53(6):417-25.

de Jonge M, Busecke G, Heinecke A, Bettendorf O. Human papillomavirus genotype distribution in cytologically screened women from northwest Germany. Acta Cytol 2013;57(6):591-8.

de Sanjose S, Almirall R, Lloveras B, Font R, Diaz M, Munoz N, et al. Cervical human papillomavirus infection in the female population in Barcelona, Spain. Sex Transm Dis 2003;30(10):788-93.

Del Prete R, Di Taranto AM, Lipsi MR, Nirchio V, Antonetti R, Miragliotta G. Prevalence and genotypes identification of human papillomavirus infection in a population of South Italy. J Clin Virol 2008;42(2):211-4.

Delere Y, Remschmidt C, Leuschner J, Schuster M, Fesenfeld M, Schneider A, et al. Human Papillomavirus prevalence and probable first effects of vaccination in 20 to 25 year-old women in Germany: a population-based cross-sectional study via home-based self-sampling. BMC Infect Dis 2014;14:87.

Depuydt CE, Vereecken AJ, Salembier GM, Vanbrabant AS, Boels LA, van Herck E, et al. Thin-layer liquid-based cervical cytology and PCR for detecting and typing human papillomavirus DNA in Flemish women. Br J Cancer 2003;88(4):560-6.

Dobec M, Bannwart F, Kaeppeli F, Cassinotti P. Automation of the linear array HPV genotyping test and its application for routine typing of human papillomaviruses in cervical specimens of women without cytological abnormalities in Switzerland. J Clin Virol 2009;45(1):23-7.

Du J, Nasman A, Carlson JW, Ramqvist T, Dalianis T. Prevalence of human papillomavirus (HPV) types in cervical cancer 2003-2008 in Stockholm, Sweden, before public HPV vaccination. Acta Oncol 2011;50(8):1215-9.

Duvlis S, Plaseska-Karanfilska D. A variant of human papillomavirus (HPV) type 66 is common among HPV-infected women from the Republic of Macedonia. Balkan Journal of Medical Genetics 2001;4(3-4):53.

Dybikowska A, Licznerski P, Podhajska A. HPV detection in cervical cancer patients in northern Poland. Oncol Rep 2002;9(4):871-4.

Faust H, Jelen MM, Poljak M, Klavs I, Ucakar V, Dillner J. Serum antibodies to human papillomavirus (HPV) pseudovirions correlate with natural infection for 13 genital HPV types. J Clin Virol 2013;56(4):336-41.

Ferreira M, Crespo M, Martins L, Felix A. HPV DNA detection and genotyping in 21 cases of primary invasive squamous cell carcinoma of the vagina. Mod Pathol 2008;21(8):968-72.

Filipi K, Tedeschini A, Paolini F, Celicu S, Morici S, Kota M, et al. Genital human papillomavirus infection and genotype prevalence among Albanian women: a cross-sectional study. J Med Virol 2010;82(7):1192-6.

Forslund O, Antonsson A, Edlund K, van den Brule AJ, Hansson BG, Meijer CJ, et al. Population-based type-specific prevalence of high-risk human papillomavirus infection in middle-aged Swedish women. J Med Virol 2002;66(4):535-41.

Fuste V, del Pino M, Perez A, Garcia A, Torne A, Pahisa J, et al. Primary squamous cell carcinoma of the vagina: human papillomavirus detection, p16(INK4A) overexpression and clinicopathological correlations. Histopathology 2010;57(6):907-16.

Garcia-Espinosa B, Moro-Rodriguez E, Alvarez-Fernandez E. Genotype distribution of human papillomavirus (HPV) in histological sections of cervical intraepithelial neoplasia and invasive cervical carcinoma in Madrid, Spain. BMC Cancer 2012;12:533.

Gargiulo F, De Francesco MA, Schreiber C, Ciravolo G, Salinaro F, Valloncini B, et al. Prevalence and distribution of single and multiple HPV infections in cytologically abnormal cervical samples from Italian women. Virus Res 2007;125(2):176-82.

Giambi C, Donati S, Carozzi F, Salmaso S, Declich S, Atti ML, et al. A cross-sectional study to estimate high-risk human papillomavirus prevalence and type distribution in Italian women aged 18-26 years. BMC Infect Dis 2013;13:74.

Giorgi Rossi P, Bisanzi S, Paganini I, Di Iasi A, Angeloni C, Scalisi A, et al. Prevalence of HPV high and low risk types in cervical samples from the Italian general population: a population based study. BMC Infect Dis 2010;10:214.

Giorgi Rossi P, Chini F, Bisanzi S, Burroni E, Carillo G, Lattanzi A, et al. Distribution of high and low risk HPV types by cytological status: a population based study from Italy. Infect Agent Cancer 2011;6(1):2.

Giorgi Rossi P, Sideri M, Carozzi FM, Vocaturo A, Buonaguro FM, Tornesello ML, et al. HPV type distribution in invasive cervical cancers in Italy: pooled analysis of three large studies. Infect Agent Cancer 2012;7(1):26.

Giovannelli L, Vassallo R, Matranga D, Affronti M, Caleca MP, Bellavia C, et al. Prevalence of cervical human papillomavirus infection and types among women immigrated to Sicily, Italy. Acta Obstet Gynecol Scand 2009;88(6):737-42.

Giuffre G, Simone A, Todaro P, Le Donne M, Caruso C, Pizzo A, et al. Detection and genotyping of human papillomavirus in gynaecologic outpatients of Messina, eastern Sicily, Italy. Oncol Rep 2010;23(3):745-50.

Goldman B, Rebolj M, Rygaard C, Preisler S, Ejegod DM, Lynge E, et al. Patterns of cervical coinfection with multiple human papilloma virus types in a screening population in Denmark. Vaccine 2013;31(12):1604-9.

Gonzalez-Bosquet E, Esteva C, Munoz-Almagro C, Ferrer P, Perez M, Lailla JM. Identification of vaccine human papillomavirus genotypes in squamous intraepithelial lesions (CIN2-3). Gynecol Oncol 2008;111(1):9-12.

Grahovac M, Racic I, Hadzisejdic I, Doric A, Grahovac B. Prevalence of human papillomavirus among Croatian women attending regular gynecological visit. Coll Antropol 2007;31 Suppl 2:73-7.

Gudleviciene Z, Didziapetriene J, Ramael M, Uleckiene S, Valuckas KP. Human papillomavirus and p53 polymorphism in Lithuanian cervical cancer patients. Gynecol Oncol 2006;102(3):530-3.

Gudleviciene Z, Kanopiene D, Didziapetriene J, Smolyakova R, Gutkovskaya E, Zhukovec A, et al. Differences on the prevalence of cervical HPV between Lithuania and Belarus. Cent Eur J Med 2014;9(2):285-91.

Guido M, Tinelli A, De DA, Bruno AR, Tagliaferro L, Fedele A, et al. Prevalence and distribution of human papillomavirus genotype in south eastern Italy, in the period 2006-2011: implications for intervention. Curr Pharm Des 2013;19(8):1498-507.

Hadzisejdic I, Simat M, Bosak A, Krasevic M, Grahovac B. Prevalence of human papillomavirus genotypes in cervical cancer and precursor lesions. Coll Antropol 2006;30(4):879-83.

Hadzisejdc I, Krasevic M, Haller H, Grahovac B. Distribution of human papillomavirus types in different histological subtypes of cervical adenocarcinoma. Coll Antropol 2007;31 Suppl 2:97-102.

Hall JS, Iype R, Armenoult LS, Taylor J, Miller CJ, Davidson S, et al. Poor prognosis associated with human papillomavirus alpha7 genotypes in cervical carcinoma cannot be explained by intrinsic radiosensitivity. Int J Radiat Oncol Biol Phys 2013;85(5):e223-e229.

Hampl M, Sarajuuri H, Wentzensen N, Bender HG, Kueppers V. Effect of human papillomavirus vaccines on vulvar, vaginal, and anal intraepithelial lesions and vulvar cancer. Obstet Gynecol 2006;108(6):1361-8.

Hampl M, Deckers-Figiel S, Hampl JA, Rein D, Bender HG. New aspects of vulvar cancer: changes in localization and age of onset. Gynecol Oncol 2008;109(3):340-5.

Heard I, Tondeur L, Arowas L, Falguieres M, Demazoin MC, Favre M. Human papillomavirus types distribution in organised cervical cancer screening in france. PLoS One 2013;8(11):e79372.

Hellman K, Lindquist D, Ranhem C, Wilander E, Andersson S. Human papillomavirus, p16(INK4A), and Ki-67 in relation to clinicopathological variables and survival in primary carcinoma of the vagina. Br J Cancer 2014;110(6):1561-70.

Herraez-Hernandez E, Alvarez-Perez M, Navarro-Bustos G, Esquivias J, Alonso S, Aneiros-Fernandez J, et al. HPV Direct Flow CHIP: a new human papillomavirus genotyping method based on direct PCR from crude-cell extracts. J Virol Methods 2013;193(1):9-17.

Hibbitts S, Jones J, Powell N, Dallimore N, McRea J, Beer H, et al. Human papillomavirus prevalence in women attending routine cervical screening in South Wales, UK: a cross-sectional study. Br J Cancer 2008;99(11):1929-33.

Hibbitts S, Tristram A, Beer H, McRea J, Rose B, Hauke A, et al. UK population based study to predict impact of HPV vaccination. J Clin Virol 2014;59(2):109-14.

Houghton O, Jamison J, Wilson R, Carson J, McCluggage WG. p16 Immunoreactivity in unusual types of cervical adenocarcinoma does not reflect human papillomavirus infection. Histopathology 2010;57(3):342-50.

Howell-Jones R, Bailey A, Beddows S, Sargent A, de Silva N, Wilson G, et al. Multi-site study of HPV type-specific prevalence in women with cervical cancer, intraepithelial neoplasia and normal cytology, in England. Br J Cancer 2010;103(2):209-16.

Howell-Jones R, de SN, Akpan M, Oakeshott P, Carder C, Coupland L, et al. Prevalence of human papillomavirus (HPV) infections in sexually active adolescents and young women in England, prior to widespread HPV immunisation. Vaccine 2012;30(26):3867-75.

Iftner T, Eberle S, Iftner A, Holz B, Banik N, Quint W, et al. Prevalence of low-risk and high-risk types of human papillomavirus and other risk factors for HPV infection in Germany within different age groups in women up to 30 years of age: an epidemiological observational study. J Med Virol 2010;82(11):1928-39.

Ivansson EL, Gustavsson IM, Wilander E, Magnusson PK, Gyllensten UB. Temporal trends over 3 decades and intrafamilial clustering of HPV types in Swedish patients with cervical cancer in situ. Int J Cancer 2009;125(12):2930-5.

Jacobs MV, Walboomers JM, Snijders PJ, Voorhorst FJ, Verheijen RH, Fransen-Daalmeijer N, et al. Distribution of 37 mucosotropic HPV types in women with cytologically normal cervical smears: the age-related patterns for high-risk and low-risk types. Int J Cancer 2000;87(2):221-7.

Jalal H, Stephen H, Bibby DF, Sonnex C, Carne CA. Molecular epidemiology of genital human papillomavirus and Chlamydia trachomatis among patients attending a genitourinary medicine clinic - will vaccines protect? Int J STD AIDS 2007;18(9):617-21.

Jancar N, Kocjan BJ, Poljak M, Lunar MM, Bokal EV. Distribution of human papillomavirus genotypes in women with cervical cancer in Slovenia. Eur J Obstet Gynecol Reprod Biol 2009;145(2):184-8.

Jovanovic AM, Dikic SD, Jovanovic V, Zamurovic M, Nikolic B, Krsic V, et al. Correlation of human papilloma virus infection with cytology, colposcopy and histopathological examination of the bioptic tissue in low- and high-grade intraepithelial lesions. Eur J Gynaecol Oncol 2012;33(5):512-6.

Kaliterna V, Andelinovic S, Pejkovic L, Hofman ID. Human papillomavirus DNA typing in the cervical specimens among women of Split and Dalmatian County. Coll Antropol 2007;31 Suppl 2:79-82.

Kaliterna V, Kaliterna M, Pejkovic L, Hofman ID, Andelinovic S. Prevalence and genotyping of the human papillomavirus in the cervical specimens among women of Southern Croatia (Dalmatia County). Cent Eur J Public Health 2013;21(1):26-9.

Kavanagh K, Sinka K, Cuschieri K, Love J, Potts A, Pollock KG, et al. Estimation of HPV prevalence in young women in Scotland; monitoring of future vaccine impact. BMC Infect Dis 2013;13:519.

Kavanagh K, Pollock KG, Potts A, Love J, Cuschieri K, Cubie H, et al. Introduction and sustained high coverage of the HPV bivalent vaccine leads to a reduction in prevalence of HPV 16/18 and closely related HPV types. Br J Cancer 2014;110(11):2804-11.

Keegan H, Pilkington L, McInerney J, Jeney C, Benczik M, Cleary S, et al. Human papillomavirus detection and genotyping, by HC2, full-spectrum HPV and molecular beacon real-time HPV assay in an Irish colposcopy clinic. J Virol Methods 2014;201:93-100.

Kirschner B, Junge J, Holl K, Rosenlund M, De Souza SC, Quint W, et al. HPV- genotypes in invasive cervical cancer in Danish women. Acta Obstet Gynecol Scand 2013;92(9):1023-31.

Kirschner B, Schledermann D, Holl K, Rosenlund M, Raillard A, Quint W, et al. HPV-genotypes in high-grade intraepithelial cervical lesions in Danish women. Acta Obstet Gynecol Scand 2013; 92(9):1032-40.

Kjaer SK, van den Brule AJ, Paull G, Svare EI, Sherman ME, Thomsen BL, et al. Type specific persistence of high risk human papillomavirus (HPV) as indicator of high grade cervical squamous intraepithelial lesions in young women: population based prospective follow up study. BMJ 2002;325(7364):572

Kjaer SK, Breugelmans G, Munk C, Junge J, Watson M, Iftner T. Population-based prevalence, type- and age-specific distribution of HPV in women before introduction of an HPV-vaccination program in Denmark. Int J Cancer 2008;123(8):1864-70.

Kjaer SK, Frederiksen K, Munk C, Iftner T. Long-term absolute risk of cervical intraepithelial neoplasia grade 3 or worse following human papillomavirus infection: role of persistence. J Natl Cancer Inst 2010;102(19):1478-88.

Kjaer SK, Munk C, Junge J, Iftner T. Carcinogenic HPV prevalence and age-specific type distribution in 40,382 women with normal cervical cytology, ASCUS/LSIL, HSIL, or cervical cancer: what is the potential for prevention? Cancer Causes Control 2014;25(2):179-89.

Klemba A, Kowalewska M, Kukwa W, Tonska K, Szybinska A, Mossakowska M, et al. Mitochondrial genotype in vulvar carcinoma - cuckoo in the nest. J Biomed Sci 2010;17:73.

Klug SJ, Hukelmann M, Hollwitz B, Duzenli N, Schopp B, Petry KU, et al. Prevalence of human papillomavirus types in women screened by cytology in Germany. J Med Virol 2007;79(5):616-25.

Konidaris S, Kouskouni EE, Panoskaltsis T, Kreatsas G, Patsouris ES, Sarivalassis A, et al. Human papillomavirus infection in malignant and benign gynaecological conditions: a study in Greek women. Health Care Women Int 2007;28(2):182-91.

Kovachev S, Slavov V, Slavova K. Prevalence of human papillomavirus infection in women in some cities and regions of Bulgaria. J Med Virol 2013;85(9):1577-84.

Kovanda A, Juvan U, Sterbenc A, Kocjan BJ, Seme K, Jancar N, et al. Pre-vaccination distribution of human papillomavirus (HPV) genotypes in women with cervical intraepithelial neoplasia grade 3 (CIN 3) lesions in Slovenia. Acta Dermatovenerol Alp Panonica Adriat 2009;18(2):47-52.

Kowalewska M, Szkoda MT, Radziszewski J, Ptaszynski K, Bidzinski M, Siedlecki JA. The frequency of human papillomavirus infection in polish patients with vulvar squamous cell carcinoma. Int J Gynecol Cancer 2010;20(3):434-7.

Kraus I, Molden T, Holm R, Lie AK, Karlsen F, Kristensen GB, et al. Presence of E6 and E7 mRNA from human papillomavirus types 16, 18, 31, 33, and 45 in the majority of cervical carcinomas. J Clin Microbiol 2006;44(4):1310-7.

Kulmala SM, Shabalova IP, Petrovitchev N, Syrjanen KJ, Gyllensten UB, Syrjanen SM. Prevalence of the most common high-risk HPV genotypes among women in three new independent states of the former Soviet Union. J Med Virol 2007;79(6):771-81.

Larsson GL, Helenius G, Andersson S, Elgh F, Sorbe B, Karlsson MG. Human papillomavirus (HPV) and HPV 16-variant distribution in vulvar squamous cell carcinoma in Sweden. Int J Gynecol Cancer 2012;22(8):1413-9.

Larsson GL, Helenius G, Andersson S, Sorbe B, Karlsson MG. Prognostic impact of human papilloma virus (HPV) genotyping and HPV-16 subtyping in vaginal carcinoma. Gynecol Oncol 2013;129(2):406-11.

Le Donne M, Giuffre G, Caruso C, Nicotina PA, Alibrandi A, Scalisi R, et al. Human Papillomavirus Types Distribution in Eastern Sicilian Females with cervical lesions. A Correlation with Colposcopic and Histological Findings. Pathol Oncol Res 2013;19(3):481-7.

Leinonen MK, Anttila A, Malila N, Dillner J, Forslund O, Nieminen P. Type- and age-specific distribution of human papillomavirus in women attending cervical cancer screening in Finland. Br J Cancer 2013;109(11):2941-50.

Lenselink CH, Melchers WJ, Quint WG, Hoebers AM, Hendriks JC, Massuger LF, et al. Sexual behaviour and HPV infections in 18 to 29 year old women in the pre-vaccine era in the Netherlands. PLoS ONE 2008;3(11):e3743.

Lindell G, Nasman A, Jonsson C, Ehrsson RJ, Jacobsson H, Danielsson KG, et al. Presence of human papillomavirus (HPV) in vulvar squamous cell carcinoma (VSCC) and sentinel node. Gynecol Oncol 2010;117(2):312-6.

Louvanto K, Rintala MA, Syrjanen KJ, Grenman SE, Syrjanen SM. Incident cervical infections with high- and low-risk human papillomavirus (HPV) infections among mothers in the prospective Finnish Family HPV Study. BMC Infect Dis 2011;11:179.

Lukaszuk K, Liss J, Wozniak I, Sliwinski W, Emerich J, Wojcikowski C. HPV and histological status of pelvic lymph node metastases in cervical cancer: a prospective study. J Clin Pathol 2004;57(5):472-6.

Mariani L, Monfulleda N, Alemany L, Vizza E, Marandino F, Vocaturo A, et al. Human papillomavirus prevalence and type-specific relative contribution in invasive cervical cancer specimens from Italy. BMC Cancer 2010;10:259.

Martin P, Kilany L, Garcia D, Lopez-Garcia AM, Martin-Azana MJ, Abraira V, et al. Human papillomavirus genotype distribution in Madrid and correlation with cytological data. BMC Infect Dis 2011;11:316.

Martorell M, Garcia-Garcia JA, Ortiz C, Perez-Valles A, Calabuig C, Gomez-Cabrero D, et al. Prevalence and distribution of human papillomavirus findings in swab specimens from gynaecology clinics of the east coast of Spain. Scand J Infect Dis 2010;42(6-7):549-53.

Martro E, Valencia MJ, Tarrats A, Castella E, Llatjos M, Franquesa S, et al. Comparison between two human papillomavirus genotyping assays targeting the L1 or E6/E7 region in cervical cancer biopsies. Enferm Infecc Microbiol Clin 2012;30(5):225-9.

Mateos Lindemann ML, Sanchez Calvo JM, Chacon de AJ, Sanz I, Diaz E, Rubio MD, et al. Prevalence and Distribution of High-Risk Genotypes of HPV in Women with Severe Cervical Lesions in Madrid, Spain: Importance of Detecting Genotype 16 and Other High-Risk Genotypes. Adv Prev Med 2011;2011:269468.

Mazarico E, Gonzalez-Bosquet E. Prevalence of infection by different genotypes of human papillomavirus in women with cervical pathology. Gynecol Oncol 2012;125(1):181-5.

Mejlhede N, Bonde J, Fomsgaard A. High frequency of multiple HPV types in cervical specimens from Danish women. APMIS 2009;117(2):108-14.

Menegazzi P, Barzon L, Palu G, Reho E, Tagliaferro L. Human papillomavirus type distribution and correlation with cyto-histological patterns in women from the South of Italy. Infect Dis Obstet Gynecol 2009;2009:198425.

Mesher D, Soldan K, Howell-Jones R, Panwar K, Manyenga P, Jit M, et al. Reduction in HPV 16/18 prevalence in sexually active young women following the introduction of HPV immunisation in England. Vaccine 2013;32(1):26-32.

Michala L, Argyri E, Tsimplaki E, Tsitsika A, Bakoula C, Antsaklis A, et al. Human Papilloma Virus infection in sexually active adolescent girls. Gynecol Oncol 2012;126(2):207-10.

Milanova E, Naumov J, Stojovski M, Todorovska I, Daneva K. Operative treatment of cervical premalignant lesions and the presence of high-risk human papilloma virus as etiologic agent. Bratisl Lek Listy 2004;105(10-11):365-7.

Molden T, Kraus I, Karlsen F, Skomedal H, Nygard JF, Hagmar B. Comparison of human papillomavirus messenger RNA and DNA detection: a cross-sectional study of 4,136 women >30 years of age with a 2-year follow-up of high-grade squamous intraepithelial lesion. Cancer Epidemiol Biomarkers Prev 2005;14(2):367-72.

Mollers M, Scherpenisse M, van der Klis FR, King AJ, van Rossum TG, van Logchem EM, et al. Prevalence of genital HPV infections and HPV serology in adolescent girls, prior to vaccination. Cancer Epidemiol 2012;36(6):519-24.

Mollers M, Boot HJ, Vriend HJ, King AJ, van den Broek Ingrid VF, van Bergen Jan EA, et al. Prevalence, incidence and persistence of genital HPV infections in a large cohort of sexually active young women in the Netherlands. Vaccine 2013;31(2):394-401.

Monsonego J, Zerat L, Syrjanen K, Zerat JC, Smith JS, Halfon P. Prevalence of type-specific human papillomavirus infection among women in France: Implications for screening, vaccination, and a future generation of multivalent HPV vaccines. Vaccine 2012;30(35):5215-21.

Murphy N, Ring M, Killalea AG, Uhlmann V, O'Donovan M, Mulcahy F, et al. p16INK4A as a marker for cervical dyskaryosis: CIN and cGIN in cervical biopsies and ThinPrep smears. J Clin Pathol 2003;56(1):56-63.

Nielsen A, Kjaer SK, Munk C, Iftner T. Type-specific HPV infection and multiple HPV types: prevalence and risk factor profile in nearly 12,000 younger and older Danish women. Sex Transm Dis 2008;35(3):276-82.

Nielsen A, Iftner T, Munk C, Kjaer SK. Acquisition of high-risk human papillomavirus infection in a population-based cohort of Danish women. Sex Transm Dis 2009;36(10):609-15.

Nobre RJ, Cruz E, Real O, de Almeida LP, Martins TC. Characterization of common and rare human papillomaviruses in Portuguese women by the polymerase chain reaction, restriction fragment length polymorphism and sequencing. J Med Virol 2010;82(6):1024-32.

Oakeshott P, Aghaizu A, Reid F, Howell-Jones R, Hay PE, Sadiq ST, et al. Frequency and risk factors for prevalent, incident, and persistent genital carcinogenic human papillomavirus infection in sexually active women: community based cohort study. BMJ 2012;344:e4168.

Oliveira A, Verdasca N, Pista A. Use of the NucliSENS EasyQ HPV assay in the management of cervical intraepithelial neoplasia. J Med Virol 2013;85(7):1235-41.

Orlando G, Fasolo M, Mazza F, Ricci E, Esposito S, Frati E, et al. Risk of cervical HPV infection and prevalence of vaccine-type and other high-risk HPV types among sexually active teens and young women (13-26 years) enrolled in the VALHIDATE study. Hum Vaccin Immunother 2014;10(4):986-94.

Ortiz M, Torres M, Munoz L, Fernandez-Garcia E, Canals J, Cabornero AI, et al. Oncogenic human papillomavirus (HPV) type distribution and HPV type 16 E6 variants in two Spanish population groups with different levels of HPV infection risk. J Clin Microbiol 2006;44(4):1428-34.

Otero-Motta AP, Ordonez JL, Gonzalez-Celador R, Rivas B, Macias MC, Bullon A, et al. Prevalence of human papillomavirus genotypes in cytologic abnormalities from unvaccinated women living in north-western Spain. APMIS 2011;119(3):204-15.

Panatto D, Amicizia D, Tanzi E, Bianchi S, Frati ER, Zotti CM, et al. Prevalence of human papillomavirus in young Italian women with normal cytology: how should we adapt the national vaccination policy? BMC Infect Dis 2013;13:575.

Pannier-Stockman C, Segard C, Bennamar S, Gondry J, Boulanger JC, Sevestre H, et al. Prevalence of HPV genotypes determined by PCR and DNA sequencing in cervical specimens from French women with or without abnormalities. J Clin Virol 2008;42(4):353-60.

Panotopoulou E, Tserkezoglou A, Kouvousi M, Tsiaousi I, Chatzieleftheriou G, Daskalopoulou D, et al. Prevalence of human papillomavirus types 6, 11, 16, 18, 31, and 33 in a cohort of Greek women. J Med Virol 2007;79(12):1898-905.

Perez C, Klaustermeier JE, Alemany L, Tous S, de SS, Velasco J. Comparison of 2 different PCR-based technologies for the detection of human papilloma virus from paraffin-embedded tissue: genomica clinical arrays versus SPF(10)-LiPA(25). Diagn Mol Pathol 2012;21(1):45-52.

Perez C, Castillo M, Alemany L, Tous S, Klaustermeier J, de SS, et al. Evaluation of p16INK4a Overexpression in a Large Series of Cervical Carcinomas: Concordance With SPF10-LiPA25 PCR. Int J Gynecol Pathol 2014;33(1):74-82.

Perez-Castro S, Lorenzo-Mahia Y, Inarrea FA, Lamas-Gonzalez MJ, Saran-Diez MT, Rubio-Alarcon J, et al. Cervical intraepithelial neoplasia grade 2 or worse in Galicia, Spain: HPV 16 prevalence and vaccination impact. Enferm Infecc Microbiol Clin 2014;32(8):479-85.

Pete I, Szirmai K, Csapo Z, Szantho A, Fule T, Gallai M, et al. Detection of high-risk HPV (16, 18, 33) in situ cancer of the cervix by PCR technique. Eur J Gynaecol Oncol 2002;23(1):74-8.

Petry KU, Luyten A, Justus A, Iftner A, Strehlke S, Reinecke-Luthge A, et al. Prevalence of high-risk HPV types and associated genital diseases in women born in 1988/89 or 1983/84--results of WOLVES, a population-based epidemiological study in Wolfsburg, Germany. BMC Infect Dis 2013;13:135.

Piana A, Sotgiu G, Castiglia P, Pischedda S, Cocuzza C, Capobianco G, et al. Prevalence and type distribution of human papillomavirus infection in women from North Sardinia, Italy. BMC Public Health 2011;11:785.

Piana A, Sotgiu G, Cocuzza C, Musumeci R, Marras V, Pischedda S, et al. High HPV-51 Prevalence in Invasive Cervical Cancers: Results of a Pre-Immunization Survey in North Sardinia, Italy. PLoS ONE 2013;8(5):e63395.

Pista A, de Oliveira CF, Cunha MJ, Paixao MT, Real O. Prevalence of human papillomavirus infection in women in Portugal: the CLEOPATRE Portugal study. Int J Gynecol Cancer 2011;21(6):1150-8.

Pista A, Oliveira A, Verdasca N, Ribeiro F. Single and multiple human papillomavirus infections in cervical abnormalities in Portuguese women. Clin Microbiol Infect 2011;17(6):941-6.

Pista A, de Oliveira CF, Lopes C, Cunha MJ. Human papillomavirus type distribution in cervical intraepithelial neoplasia grade 2/3 and cervical cancer in Portugal: a CLEOPATRE II Study. Int J Gynecol Cancer 2013;23(3):500-6.

Powell NG, Hibbitts SJ, Boyde AM, Newcombe RG, Tristram AJ, Fiander AN. The risk of cervical cancer associated with specific types of human papillomavirus: a case-control study in a UK population. Int J Cancer 2011;128(7):1676-82.

Powell N, Cuschieri K, Cubie H, Hibbitts S, Rosillon D, De Souza SC, et al. Cervical cancers associated with human papillomavirus types 16, 18 and 45 are diagnosed in younger women than cancers associated with other types: a cross-sectional observational study in Wales and Scotland (UK). J Clin Virol 2013;58(3):571-4.

Pretet JL, Jacquard AC, Carcopino X, Charlot JF, Bouhour D, Kantelip B, et al. Human papillomavirus (HPV) genotype distribution in invasive cervical cancers in France: EDITH study. Int J Cancer 2008;122(2):428-32.

Pretet JL, Jacquard AC, Carcopino X, Monnier-Benoit S, Averous G, Soubeyrand B, et al. Human papillomavirus genotype distribution in high grade cervical lesions (CIN 2/3) in France: EDITH study. Int J Cancer 2008;122(2):424-7.

Ramqvist T, Du J, Lunden M, Ahrlund-Richter S, Ferreira J, Marions L, et al. Pre-vaccination prevalence of human papillomavirus types in the genital tract of 15-23-year-old women attending a youth health clinic in Stockholm, Sweden. Scand J Infect Dis 2011;43(2):115-21.

Reesink-Peters N, Burger MP, Kleter B, Quint WG, Bossuyt PM, Adriaanse AH. Using a new HPV detection system in epidemiological research: change of views on cervical dyskaryosis? Eur J Obstet Gynecol Reprod Biol 2001;98(2):199-204.

Reuschenbach M, Roos J, Panayotopoulos D, Baldus SE, Schnurch HG, Berger A, et al. Characterization of squamous cell cancers of the vulvar anterior fourchette by human papillomavirus, p16INK4a, and p53. J Low Genit Tract Dis 2013;17(3):289-97.

Ribaldone R, Boldorini R, Capuano A, Arrigoni S, Di Oto A, Surico N. Role of HPV testing in the follow-up of women treated for cervical dysplasia. Arch Gynecol Obstet 2010;282(2):193-7.

Ripabelli G, Grasso GM, Del R, I, Tamburro M, Sammarco ML. Prevalence and genotype identification of human papillomavirus in women undergoing voluntary cervical cancer screening in Molise, Central Italy. Cancer Epidemiol 2010;34(2):162-7.

Roberts CC, Tadesse AS, Sands J, Halvorsen T, Schofield TL, Dalen A, et al. Detection of HPV in Norwegian cervical biopsy specimens with type-specific PCR and reverse line blot assays. J Clin Virol 2006;36(4):277-82.

Roccio M, Dal BB, Gardella B, Carrara M, Gulminetti R, Mariani B, et al. HPV infection and intraepithelial lesions: comparison between HIV positive and negative women. Curr HIV Res 2012;10(7):614-9.

Rogovskaya SI, Shabalova IP, Mikheeva IV, Minkina GN, Podzolkova NM, Shipulina OY, et al. Human Papillomavirus Prevalence and Type-Distribution, Cervical Cancer Screening Practices and Current Status of Vaccination Implementation in Russian Federation, the Western Countries of the former Soviet Union, Caucasus Region and Central Asia. Vaccine 2013;31 Suppl 7:H46-H58.

Ronco G, Ghisetti V, Segnan N, Snijders PJ, Gillio-Tos A, Meijer CJ, et al. Prevalence of human papillomavirus infection in women in Turin, Italy. Eur J Cancer 2005;41(2):297-305.

Rossler L, Reich O, Horvat R, De Souza SC, Holl K, Joura EA. Human papillomavirus in high-grade cervical lesions: Austrian data of a European multicentre study. Wien Klin Wochenschr 2013;125(19-20):591-9.

Sandri MT, Riggio D, Salvatici M, Passerini R, Zorzino L, Boveri S, et al. Typing of human papillomavirus in women with cervical lesions: prevalence and distribution of different genotypes. J Med Virol 2009;81(2):271-7.

Sargent A, Bailey A, Almonte M, Turner A, Thomson C, Peto J, et al. Prevalence of type-specific HPV infection by age and grade of cervical cytology: data from the ARTISTIC trial. Br J Cancer 2008;98(10):1704-9.

Schmeink CE, Massuger LF, Lenselink CH, Quint WG, Witte BI, Berkhof J, et al. Prospective follow-up of 2,065 young unscreened women to study human papillomavirus incidence and clearance. Int J Cancer 2013;133(1):172-81.

Schmitt M, Depuydt C, Benoy I, Bogers J, Antoine J, Arbyn M, et al. Prevalence and viral load of 51 genital human papillomavirus types and three subtypes. Int J Cancer 2013;132(10):2395-403.

Shipitsyna E, Zolotoverkhaya E, Kuevda D, Nasonova V, Romanyuk T, Khachaturyan A, et al. Prevalence of high-risk human papillomavirus types and cervical squamous intraepithelial lesions in women over 30 years of age in St. Petersburg, Russia. Cancer Epidemiol 2011;35(2):160-4.

Sideri M, Cristoforoni P, Casadio C, Boveri S, Igidbashian S, Schmitt M, et al. Distribution of human papillomavirus genotypes in invasive cervical cancer in Italy: a representative, single institution case series. Vaccine 2009;27 Suppl 1:A30-A33.

Sigurdsson K, Taddeo FJ, Benediktsdottir KR, Olafsdottir K, Sigvaldason H, Oddsson K, et al. HPV genotypes in CIN 2-3 lesions and cervical cancer: a population-based study. Int J Cancer 2007;121(12):2682-7.

Silins I, Wang X, Tadesse A, Jansen KU, Schiller JT, Avall-Lundqvist E, et al. A population-based study of cervical carcinoma and HPV infection in Latvia. Gynecol Oncol 2004;93(2):484-92.

Silva J, Ribeiro J, Sousa H, Cerqueira F, Teixeira AL, Baldaque I, et al. Oncogenic HPV Types Infection in Adolescents and University Women from North Portugal: From Self-Sampling to Cancer Prevention. J Oncol 2011;2011:953469.

Sjoeborg KD, Trope A, Lie AK, Jonassen CM, Steinbakk M, Hansen M, et al. HPV genotype distribution according to severity of cervical neoplasia. Gynecol Oncol 2010;118(1):29-34.

Skapa P, Zamecnik J, Hamsikova E, Salakova M, Smahelova J, Jandova K, et al. Human papillomavirus (HPV) profiles of vulvar lesions: possible implications for the classification of vulvar squamous cell carcinoma precursors and for the efficacy of prophylactic HPV vaccination. Am J Surg Pathol 2007;31(12):1834-43.

Soderlund-Strand A, Dillner J. High-throughput monitoring of human papillomavirus type distribution. Cancer Epidemiol Biomarkers Prev 2013;22(2):242-50.

Soderlund-Strand A, Kjellberg L, Dillner J. Human papillomavirus type-specific persistence and recurrence after treatment for cervical dysplasia. J Med Virol 2014;86(4):634-41.

Spinillo A, Dal Bello B, Alberizzi P, Cesari S, Gardella B, Roccio M, et al. Clustering patterns of human papillomavirus genotypes in multiple infections. Virus Res 2009;142(1-2):154-9.

Spinillo A, Gardella B, Roccio M, Alberizzi P, Silini EM, Dal BB. Untypable human papillomavirus infection and risk of cervical intraepithelial neoplasia among women with abnormal cervical cytology. J Med Virol 2014;86(7):1145-52.

Stamataki P, Papazafiropoulou A, Elefsiniotis I, Giannakopoulou M, Brokalaki H, Apostolopoulou E, et al. Prevalence of HPV infection among Greek women attending a gynecological outpatient clinic. BMC Infect Dis 2010;10:27.

Stojanovic J, Magic Z, Milacic M, Nenadic D, Stanimirovic B, Vukicevic D. Distribution of high-risk HPV types in Yugoslav women with cervical neoplasia. J BUON 2002;7(3):251-6.

Sundstrom K, Eloranta S, Sparen P, Arnheim DL, Gunnell A, Lindgren A, et al. Prospective study of human papillomavirus (HPV) types, HPV persistence, and risk of squamous cell carcinoma of the cervix. Cancer Epidemiol Biomarkers Prev 2010;19(10):2469-78.

Szostek S, Klimek M, Zawilinska B, Kosz-Vnenchak M. Genotype-specific human papillomavirus detection in cervical smears. Acta Biochim Pol 2008;55(4):687-92.

Tachezy R, Smahelova J, Salakova M, Arbyn M, Rob L, Skapa P, et al. Human papillomavirus genotype distribution in Czech women and men with diseases etiologically linked to HPV. PLoS ONE 2011;6(7):e21913.

Tachezy R, Smahelova J, Kaspirkova J, Salakova M. Human papillomavirus type-specific prevalence in the cervical cancer screening population of Czech women. PLoS One 2013;8(11):e79156.

Tamalet C, Richet H, Carcopino X, Henry M, Leretraite L, Heid P, et al. Testing for human papillomavirus and measurement of viral load of HPV 16 and 18 in self-collected vaginal swabs of women who do not undergo cervical cytological screening in Southern France. J Med Virol 2010;82(8):1431-7.

Tamalet C, Le RL, Leandri FX, Heid P, Sancho GH, Piana L. Vaginal self-sampling is an adequate means of screening HR-HPV types in women not participating in regular cervical cancer screening. Clin Microbiol Infect 2013;19(1):E44-E50.

Tempfer C, Grimm C, Harwanegg C, Huber M, Mueller MW, Buerkle B, et al. Frequency of 23 human papillomavirus types using DNA microarray in women with and without cytological anomalies. Anticancer Research 2007;27(3 B):1721-6.

Tjalma WA, Fiander A, Reich O, Powell N, Nowakowski AM, Kirschner B, et al. Differences in human papillomavirus type distribution in high-grade cervical intraepithelial neoplasia and invasive cervical cancer in Europe. Int J Cancer 2013;132(4):854-67.

Tornesello ML, Duraturo ML, Botti G, Greggi S, Piccoli R, De Palo G, et al. Prevalence of alpha-papillomavirus genotypes in cervical squamous intraepithelial lesions and invasive cervical carcinoma in the Italian population. J Med Virol 2006;78(12):1663-72.

Tornesello ML, Cassese R, De RN, Buonaguro L, Masucci A, Vallefuoco G, et al. High prevalence of human papillomavirus infection in Eastern European and West African women immigrants in South Italy. APMIS 2011;119(10):701-9.

Tornesello ML, Losito S, Benincasa G, Fulciniti F, Botti G, Greggi S, et al. Human papillomavirus (HPV) genotypes and HPV16 variants and risk of adenocarcinoma and squamous cell carcinoma of the cervix. Gynecol Oncol 2011;121(1):32-42.

Tsimplaki E, Argyri E, Michala L, Kouvousi M, Apostolaki A, Magiakos G, et al. Human papillomavirus genotyping and e6/e7 mRNA expression in greek women with intraepithelial neoplasia and squamous cell carcinoma of the vagina and vulva. J Oncol 2012;2012:893275.

Tsiodras S, Georgoulakis J, Chranioti A, Voulgaris Z, Psyrri A, Tsivilika A, et al. Hybrid Capture vs. PCR screening of Cervical Human Papilloma Virus Infections. Cytological and Histological associations in 1270 women. BMC Cancer 2010;10(1):53.

Tsiodras S, Hatzakis A, Spathis A, Margari N, Meristoudis C, Chranioti A, et al. Molecular epidemiology of HPV infection using a clinical array methodology in 2952 women in Greece. Clin Microbiol Infect 2011;17(8):1185-8.

Ucakar V, Poljak M, Klavs I. Pre-vaccination prevalence and distribution of high-risk human papillomavirus (HPV) types in Slovenian women: a cervical cancer screening based study. Vaccine 2012;30(2):116-20.

Uuskula A, Kals M, Kosenkranius L, McNutt LA, Dehovitz J. Population-based type-specific prevalence of high-risk human papillomavirus infection in Estonia. BMC Infect Dis 2010;10(1):63.

van der Avoort I, Shirango H, Hoevenaars BM, Grefte JM, de Hullu JA, de Wilde PC, et al. Vulvar squamous cell carcinoma is a multifactorial disease following two separate and independent pathways. Int J Gynecol Pathol 2006;25(1):22-9.

van Esch EM, Dam MC, Osse ME, Putter H, Trimbos BJ, Fleuren G, et al. Clinical characteristics associated with development of recurrence and progression in usual-type vulvar intraepithelial neoplasia. Int J Gynecol Cancer 2013;23(8):1476-83.

Van Seters M, ten Kate FJ, Van Beurden M, Verheijen RH, Meijer CJ, Burger MP, et al. In the absence of (early) invasive carcinoma, vulvar intraepithelial neoplasia associated with lichen sclerosus is mainly of undifferentiated type: new insights in histology and aetiology. J Clin Pathol 2007;60(5):504-9.

Van Seters M, Beckmann I, Heijmans-Antonissen C, van BM, Ewing PC, Zijlstra FJ, et al. Disturbed patterns of immunocompetent cells in usual-type vulvar intraepithelial neoplasia. Cancer Res 2008;68(16):6617-22.

Vaucel E, Coste-Burel M, Laboisse C, Dahlab A, Lopes P. Human papillomavirus genotype distribution in cervical samples collected in routine clinical practice at the Nantes University Hospital, France. Arch Gynecol Obstet 2011;284(4):989-98.

Venturoli S, Ambretti S, Cricca M, Leo E, Costa S, Musiani M, et al. Correlation of high-risk human papillomavirus genotypes persistence and risk of residual or recurrent cervical disease after surgical treatment. J Med Virol 2008;80(8):1434-40.

Verteramo R, Pierangeli A, Calzolari E, Patella A, Recine N, Mancini E, et al. Direct sequencing of HPV DNA detected in gynaecologic outpatients in Rome, Italy. Microbes Infect 2006;8(9-10):2517-21.

Verteramo R, Pierangeli A, Mancini E, Calzolari E, Bucci M, Osborn J, et al. Human Papillomaviruses and genital co-infections in gynaecological outpatients. BMC Infect Dis 2009;12(9):16.

Vieira L, Almeida A. The cytology and DNA detection by the PapilloCheck((R)) test in the diagnosis of human papillomavirus infection. Eur J Microbiol Immunol (Bp) 2013;3(1):61-7.

Vujosevic D, Vuksanovic V, Poljak M, Jokmanovic N. Human papillomavirus genotype spectrum in studied group of Montenegrin women. Acta Medica (Hradec Kralove) 2012;55(3):130-2.

Weyn C, Garbar C, Noel JC, Weynand B, Verhest A, d'Olne D, et al. Inter-laboratory variability in the presence of human papillomavirus in normal and abnormal cervical cytology samples. Cancer Epidemiol 2013;37(4):457-61.

Widschwendter A, Blassnig A, Wiedemair A, Muller-Holzner E, Muller HM, Marth C. Human papillomavirus DNA in sera of cervical cancer patients as tumor marker. Cancer Lett 2003;202(2):231-9.

Woodman CB, Collins S, Winter H, Bailey A, Ellis J, Prior P, et al. Natural history of cervical human papillomavirus infection in young women: a longitudinal cohort study. Lancet 2001;357(9271):1831-6.

Zappacosta B, Romano L, Guerriero M, Graziano M, Vitrani A, De Ninno M, et al. Detection of 14 human papillomavirus genotypes in cervical samples in women from a central-southern area of Italy showing different Pap test results. New Microbiol 2009;32(4):351-8.

Zielinski GD, Snijders PJ, Rozendaal L, Daalmeijer NF, Risse EK, Voorhorst FJ, et al. The presence of high-risk HPV combined with specific p53 and p16INK4a expression patterns points to high-risk HPV as the main causative agent for adenocarcinoma in situ and adenocarcinoma of the cervix. J Pathol 2003;201(4):535-43.

1.4 Latin America and the Caribbean

Afonso LA, Rocha WM, Carestiato FN, Dobao EA, Pesca LF, Passos MR, et al. Human papillomavirus infection among sexual partners attending a Sexually Transmitted Disease Clinic in Rio de Janeiro, Brazil. Braz J Med Biol Res 2013;46(6):533-8.

Alonio LV, Picconi MA, Dalbert D, Mural J, Bartt O, Bazan G, et al. Ha-ras oncogene mutation associated to progression of papillomavirus induced lesions of uterine cervix. J Clin Virol 2003;27(3):263-9.

Amaro-Filho SM, Golub JE, Nuovo GJ, Cunha CB, Levi JE, Villa LL, et al. A comparative analysis of clinical and molecular factors with the stage of cervical cancer in a Brazilian cohort. PLoS One 2013;8(3):e57810.

Amaro Filho SM, Nuovo GJ, Cunha CB, Pereira LD, Oliveira-Silva M, Russomano F, et al. Correlation of MCM2 detection with stage and virology of cervical cancer. Int J Biol Markers 2014;29(4):e363-71.

Andall-Brereton GM, Hosein F, Salas RA, Mohammed W, Monteil MA, Goleski V, et al. Human papillomavirus genotypes and their prevalence in a cohort of women in Trinidad. Rev Panam Salud Publica 2011;29(4):220-6.

Badano I, Pedrozo RW, Ruiz Diaz LS, Galuppo JA, Picconi MA, Campos RH, et al. Human papillomavirus (HPV) detection and Papanicolaou cytology in low-resource women in Posadas city, Misiones, Argentina. Rev Argent Microbiol 2011;43(4):263-7.

Barr E, Gause CK, Bautista OM, Railkar RA, Lupinacci LC, Insinga RP, et al. Impact of a prophylactic quadrivalent human papillomavirus (types 6, 11, 16, 18) L1 virus-like particle vaccine in a sexually active population of North American women. Am J Obstet Gynecol 2008;198(3):261-11.

Bedoya AM, Jaramillo R, Baena A, Castano J, Olaya N, Zea AH, et al. Location and density of immune cells in precursor lesions and cervical cancer. Cancer Microenvironment 2013;6(1):69-77.

Berois N, De CP, Mazal D, Sica A, Cedeira M, Caserta B, et al. Prevalence and distribution of high-risk human papillomavirus genotypes in invasive carcinoma of the uterine cervix in Uruguay. Int J Gynecol Cancer 2013;23(3):527-32.

Berois N, Heard I, Fort Z, Alonso R, Sica A, Moerzinger P, et al. Prevalence of type-specific HPV infection in Uruguay. J Med Virol 2014;86(4):647-52.

Brown CR, Leon ML, Munoz K, Fagioni A, Amador LG, Frain B, et al. Human papillomavirus infection and its association with cervical dysplasia in Ecuadorian women attending a private cancer screening clinic. Braz J Med Biol Res 2009;42(7):629-36.

Camargo M, Soto-De Leon SC, Sanchez R, Perez-Prados A, Patarroyo ME, Patarroyo MA. Frequency of human papillomavirus infection, coinfection, and association with different risk factors in Colombia. Ann Epidemiol 2011;21(3):204-13.

Canche JC, Lopez IR, Suarez NG, Acosta GC, Conde-Ferraez L, Cetina TC, et al. High prevalence and low E6 genetic variability of human papillomavirus 58 in women with cervical cancer and precursor lesions in Southeast Mexico. Mem Inst Oswaldo Cruz 2010;105(2):144-8.

Cathro HP, Loya T, Dominguez F, Howe SL, Howell R, Orndorff K, et al. Human papillomavirus profile of women in Belize City, Belize: correlation with cervical cytopathologic findings. Hum Pathol 2009;40(7):942-9.

Cavalcanti SM, Zardo LG, Passos MR, Oliveira LH. Epidemiological aspects of human papillomavirus infection and cervical cancer in Brazil. J Infect 2000;40(1):80-7.

Cecchini G, Paganini G, D'Amico M, Cannone M, Bertuletti C, Barberis MC. Cervical cancer screening programs in low-income communities. Experiences from Ecuador. Low cost detection of HPV infection in a developing country. Pathologica 2009;101(2):76-9.

Chouhy D, Gil LB, Nocito AL, Wojdyla D, Ornella L, Cittadini J, et al. Development and evaluation of a colorimetric PCR system for the detection and typing of human papillomaviruses. Int J Mol Med 2006;18(5):995-1003.

Chouhy D, D'Andrea RM, Iglesias M, Messina A, Ivancovich JJ, Cerda B, et al. Prevalence of human papillomavirus infection in Argentinean women attending two different hospitals prior to the implementation of the National Vaccination Program. J Med Virol 2013;85(4):655-66.

Ciapponi A, Bardach A, Glujovsky D, Gibbons L, Picconi MA. Type-specific HPV prevalence in cervical cancer and high-grade lesions in Latin America and the Caribbean: systematic review and meta-analysis. PLoS ONE 2011;6(10):e25493.

Clarke M, Schiffman M, Wacholder S, Rodriguez AC, Hildesheim A, Quint W. A prospective study of absolute risk and determinants of human papillomavirus incidence among young women in Costa Rica. BMC Infect Dis 2013;13:308.

Correnti M, Medina F, Cavazza ME, Rennola A, Avila M, Fernandes A. Human papillomavirus (HPV) type distribution in cervical carcinoma, low-grade, and high-grade squamous intraepithelial lesions in Venezuelan women. Gynecol Oncol 2011;121(3):527-31.

Coser J, da Rocha BT, Simon D, Kazantzi Fonseca AS, Ikuta N, Lunge VR. Prevalence and genotypic diversity of cervical human papillomavirus infection among women from an urban center in Brazil. Genet Mol Res 2013;12(4):4276-85.

da Silva MC, Martins HP, de Souza JL, Tognim MC, Svidzinski TI, Teixeira JJ, et al. Prevalence of HPV infection and genotypes in women with normal cervical cytology in the state of Parana, Brazil. Arch Gynecol Obstet 2012;286(4):1015-22.

da Silva Barros NK, Costa MC, Alves RR, Villa LL, Derchain SF, Zeferino LC, et al. Association of HPV infection and Chlamydia trachomatis seropositivity in cases of cervical neoplasia in Midwest Brazil. J Med Virol 2012;84(7):1143-50.

de Almeida FG, Machado AP, Fernandes CE, Ferreira AT, Padovani CT, Tozetti IA. Molecular epidemiology of the human papillomavirus infection in self-collected samples from young women. J Med Virol 2014;86(2):266-71.

de Oliveira CM, Fregnani JH, Carvalho JP, Longatto-Filho A, Levi JE. Human papillomavirus genotypes distribution in 175 invasive cervical cancer cases from Brazil. BMC Cancer 2013;13:357.

Deluca GD, Basiletti J, Schelover E, Vasquez ND, Alonso JM, Marin HM, et al. Chlamydia trachomatis as a probable cofactor in human papillomavirus infection in aboriginal women from northeastern Argentina. Braz J Infect Dis 2011;15(6):567-72.

Deluca GD, Basiletti J, Gonzalez JV, Diaz VN, Lucero RH, Picconi MA. Human papilloma virus risk factors for infection and genotype distribution in aboriginal women from Northern Argentina. Medicina (B Aires) 2012;72(6):461-6.

Fernandes J, Carvalho M, de FT, Araujo J, Azevedo P, Azevedo J, et al. Prevalence of human papillomavirus type 58 in women with or without cervical lesions in northeast Brazil. Ann Med Health Sci Res 2013;3(4):504-10.

Fernandes JV. Prevalence of HPV infection by cervical cytologic status in Brazil. Int J Gynaecol Obstet 2009;105(1):21-4.

Fernandes JV, Meissner RV, Carvalho MG, Fernandes TA, Azevedo PR, Sobrinho JS, et al. Prevalence of human papillomavirus in archival samples obtained from patients with cervical pre-malignant and malignant lesions from Northeast Brazil. BMC Res Notes 2010;3(1):96.

Fernandes JV, Meissner R, V, Carvalho MG, Fernandes TA, Azevedo PR, de Azevedo JW, et al. Human papillomavirus prevalence in women with normal cytology and with cervical cancer in Natal, Brazil. Mol Med Report 2011;4(6):1321-6.

Fernandez-Tilapa G, I. Prevalence of human papillomavirus types among Mexican women with intraepithelial lesions and cervical cancer: Detection with MY09/MY011 and GP5+/GP6+ primer systems. American Journal of Infectious Diseases 2007;3(2):62-7.

Ferreccio C, Prado RB, Luzoro AV, Ampuero SL, Snijders PJ, Meijer CJ, et al. Population-based prevalence and age distribution of human papillomavirus among women in Santiago, Chile. Cancer Epidemiol Biomarkers Prev 2004;13(12):2271-6.

Ferreccio C, Corvalan A, Margozzini P, Viviani P, Gonzalez C, Aguilera X, et al. Baseline assessment of prevalence and geographical distribution of HPV types in Chile using self-collected vaginal samples. BMC Public Health 2008;8:78.

Ferrera A, Tabora N, Flores Y, Zelaya A, Massuger L, Melchers WJ. Assessment of HPV infection among female university students in Honduras via Roche linear array. Int J Gynaecol Obstet 2011;113(2):96-9.

Figueiredo Alves RR, Turchi MD, Santos LE, Guimaraes EM, Garcia MM, Seixas MS, et al. Prevalence, genotype profile and risk factors for multiple human papillomavirus cervical infection in unimmunized female adolescents in Goiania, Brazil: a community-based study. BMC Public Health 2013;13(1):1041.

Garcia DA, Cid-Arregui A, Schmitt M, Castillo M, Briceno I, Aristizabal FA. Highly sensitive detection and genotyping of HPV by PCR multiplex and Luminex technology in a cohort of Colombian women with abnormal cytology. Open Virol J 2011;5:70-9.

Garcia DA, Briceno I, Castillo M, Aristizabal FA. Detection of gene amplification in MYCN, C-MYC, MYCL1, ERBB2, EGFR, AKT2, and human papilloma virus in samples from cervical smear normal cytology, intraepithelial cervical neoplasia (CIN I, II, III), and cervical cancer. Colombia Medica 2011;42(2):144-53.

Giuliano AR, Papenfuss M, Abrahamsen M, Denman C, de Zapien JG, Henze JL, et al. Human papillomavirus infection at the United States-Mexico border: implications for cervical cancer prevention and control. Cancer Epidemiol Biomarkers Prev 2001;10(11):1129-36.

Golijow CD, Abba MC, Mouron SA, Laguens RM, Dulout FN, Smith JS. Chlamydia trachomatis and Human papillomavirus infections in cervical disease in Argentine women. Gynecol Oncol 2005;96(1):181-6.

Gonzalez-Losa MdR, Rosado-Lopez I, Valdez-Gonzalez N, Puerto-Solis M. High prevalence of human papillomavirus type 58 in Mexican colposcopy patients. J Clin Virol 2004;29(3):202-5.

Herrero R, Hildesheim A, Bratti C, Sherman ME, Hutchinson M, Morales J, et al. Population-based study of human papillomavirus infection and cervical neoplasia in rural Costa Rica. J Natl Cancer Inst 2000;92(6):464-74.

Herrero R, Castle PE, Schiffman M, Bratti MC, Hildesheim A, Morales J, et al. Epidemiologic profile of type-specific human papillomavirus infection and cervical neoplasia in Guanacaste, Costa Rica. J Infect Dis 2005;191(11):1796-807.

Hindryckx P, Garcia A, Claeys P, Gonzalez C, Velasquez R, Bogers J, et al. Prevalence of high risk human papillomavirus types among Nicaraguan women with histological proved pre-neoplastic and neoplastic lesions of the cervix. Sex Transm Infect 2006;82(4):334-6.

Hosein F, Mohammed W, Zubach V, Legall G, Severini A. Human papillomavirus genotypes in invasive cervical squamous cell carcinoma in Trinidad. Rev Panam Salud Publica 2013;33(4):267-70.

Ili CG, Brebi P, Lopez J, Garcia P, Leal P, Suarez E, et al. Genotyping of human papillomavirus in cervical intraepithelial neoplasia in a high-risk population. J Med Virol 2011;83(5):833-7.

Illades-Aguiar B, Cortes-Malagon EM, Antonio-Vejar V, Zamudio-Lopez N, Alarcon-Romero LC, Fernandez-Tilapa G, et al. Cervical carcinoma in Southern Mexico: Human papillomavirus and cofactors. Cancer Detect Prev 2009;32(4):300-7.

Illades-Aguiar B, Alarcon-Romero LD, Antonio-Vejar V, Zamudio-Lopez N, Sales-Linares N, Flores-Alfaro E, et al. Prevalence and distribution of human papillomavirus types in cervical cancer, squamous intraepithelial lesions, and with no intraepithelial lesions in women from Southern Mexico. Gynecol Oncol 2010;117(2):291-6.

Kasamatsu E, Cubilla AL, Alemany L, Chaux A, Tous S, Mendoza L, et al. Type-specific human papillomavirus distribution in invasive cervical carcinomas in Paraguay. A study of 432 cases. J Med Virol 2012;84(10):1628-35.

Kightlinger RS, Irvin WP, Archer KJ, Huang NW, Wilson RA, Doran JR, et al. Cervical cancer and human papillomavirus in indigenous Guyanese women. Am J Obstet Gynecol 2010;202(6):626-7.

Krambeck WM, Cadide RM, Dalmarco EM, de Cordova CM. HPV detection and genotyping as an earlier approach in cervical cancer screening of the female genital tract. Clin Exp Obstet Gynecol 2008;35(3):175-8.

Lavorato-Rocha AM, de Melo MB, Rodrigues IS, Stiepcich MM, Baiocchi G, da Silva Cestari FM, et al. Prognostication of vulvar cancer based on p14ARF status: molecular assessment of transcript and protein. Ann Surg Oncol 2013;20(1):31-9.

Lazcano-Ponce E, Herrero R, Munoz N, Cruz A, Shah KV, Alonso P, et al. Epidemiology of HPV infection among Mexican women with normal cervical cytology. Int J Cancer 2001;91(3):412-20.

Lewis-Bell K, Luciani S, Unger ER, Hariri S, McFarlane S, Steinau M, et al. Genital human papillomaviruses among women of reproductive age in Jamaica. Rev Panam Salud Publica 2013;33(3):159-65.

Lippman SA, Sucupira MC, Jones HE, Luppi CG, Palefsky J, van de Wijgert JH, et al. Prevalence, distribution and correlates of endocervical human papillomavirus types in Brazilian women. Int J STD AIDS 2010;21(2):105-9.

Lopez-Revilla R, Martinez-Contreras LA, Sanchez-Garza M. Prevalence of high-risk human papillomavirus types in Mexican women with cervical intraepithelial neoplasia and invasive carcinoma. Infect Agent Cancer 2008;3(1):3.

Lorenzato F, Ho L, Terry G, Singer A, Santos LC, De Lucena BR, et al. The use of human papillomavirus typing in detection of cervical neoplasia in Recife (Brazil). Int J Gynecol Cancer 2000;10(2):143-50.

Lorenzato FR, Singer A, Ho L, Santos LC, Batista RL, Lubambo TM, et al. Human papillomavirus detection for cervical cancer prevention with polymerase chain reaction in self-collected samples. Am J Obstet Gynecol 2002;186(5):962-8.

Martorell M, Garcia-Garcia JA, Gomez-Cabrero D, Del AA. Comparison of the prevalence and distribution of human papillomavirus infection and cervical lesions between urban and native habitants of an Amazonian region of Peru. Genet Mol Res 2012;11(3):2099-106.

Matos E, Loria D, Amestoy GM, Herrera L, Prince MA, Moreno J, et al. Prevalence of human papillomavirus infection among women in Concordia, Argentina: a population-based study. Sex Transm Dis 2003;30(8):593-9.

Mendez K, Romaguera J, Perez CM, Soto-Salgado M, Tortolero-Luna G, Palefsky JM, et al. Cervical human papillomavirus infection in a sample of Hispanic women living in Puerto Rico: comparison with cervical cytology reports. P R Health Sci J 2013;32(1):3-7.

Mendoza LP, Arbiza J, Paez M, Kasamatsu E, Castro A, Gimenez G, et al. Distribution of human papillomavirus genotypes in Paraguayan women according to the severity of the cervical lesion. J Med Virol 2011;83(8):1351-7.

Michelli E, Tellez L, Mendoza JA, Noguera ME, Milano M, Vera R, et al. Amplification of human papillomavirus early genes for detection of nine genotypes in Venezuelan women. Invest Clin 2013;54(4):392-405.

Miranda PM, Pitol BC, Moran MS, Silva NN, Felix PM, Lima-Filho JL, et al. Human papillomavirus infection in Brazilian women with normal cervical cytology. Genet Mol Res 2012;11(2):1752-61.

Molano M, Posso H, Weiderpass E, van den Brule AJ, Ronderos M, Franceschi S, et al. Prevalence and determinants of HPV infection among Colombian women with normal cytology. Br J Cancer 2002;87(3):324-33.

Molano M, van den Brule AJ, Posso H, Weiderpass E, Ronderos M, Franceschi S, et al. Low grade squamous intra-epithelial lesions and human papillomavirus infection in Colombian women. Br J Cancer 2002;87(12):1417-21.

Molano M, Acosta PM, Bravo MM. Types and variants of human papillomavirus in patients with cervical cancer submitted to radiotherapy. Biosalud 2007;6:45-57.

Montalvo MT, Lobato I, Villanueva H, Borquez C, Navarrete D, Abarca J, et al. Prevalence of human papillomavirus in university young women. Oncol Lett 2011;2(4):701-6.

Munoz N, Mendez F, Posso H, Molano M, van den Brule AJ, Ronderos M, et al. Incidence, duration, and determinants of cervical human papillomavirus infection in a cohort of Colombian women with normal cytological results. J Infect Dis 2004;190(12):2077-87.

Murillo R, Molano M, Martinez G, Mejia JC, Gamboa O. HPV prevalence in Colombian women with cervical cancer: implications for vaccination in a developing country. Infect Dis Obstet Gynecol 2009;2009:653598.

Oliveira FA, Ehrig V, Lang K, Heukelbach J, Stoffler-Meilicke M, Ignatius R, et al. Human papillomavirus genotype distribution and risk factors for infection in women from a small municipality in north east Brazil. Int J STD AIDS 2012;23(9):e5-10.

Oliveira LH, Ferreira MD, Augusto EF, Melgaco FG, Santos LS, Cavalcanti SM, et al. Human papillomavirus genotypes in asymptomatic young women from public schools in Rio de Janeiro, Brazil. Rev Soc Bras Med Trop 2010;43(1):4-8.

Orozco-Colin A, Carrillo-Garcia A, Mendez-Tenorio A, Ponce-de-Leon S, Mohar A, Maldonado-Rodriguez R, et al. Geographical variation in human papillomavirus prevalence in Mexican women with normal cytology. Int J Infect Dis 2010;14(12):e1082-e1087.

Ortiz AP, Romaguera J, Perez CM, Otero Y, Soto-Salgado M, Mendez K, et al. Human papillomavirus infection in women in Puerto Rico: agreement between physician-collected and self-collected anogenital specimens. J Low Genit Tract Dis 2013;17(2):210-7.

Parada R, Morales R, Giuliano AR, Cruz A, Castellsague X, Lazcano-Ponce E. Prevalence, concordance and determinants of human papillomavirus infection among heterosexual partners in a rural region in central Mexico. BMC Infect Dis 2010;10:223.

Peralta-Rodriguez R, Romero-Morelos P, Villegas-Ruiz V, Mendoza-Rodriguez M, Taniguchi-Ponciano K, Gonzalez-Yebra B, et al. Prevalence of human papillomavirus in the cervical epithelium of Mexican women: meta-analysis. Infect Agent Cancer 2012;7(1):34.

Pereira CR, Rosa ML, Vasconcelos GA, Faria PC, Cavalcanti SM, Oliveira LH. Human papillomavirus prevalence and predictors for cervical cancer among high-risk women from Rio de Janeiro, Brazil. Int J Gynecol Cancer 2007;17(3):651-60.

Perez LO, Crivaro A, Barbisan G, Poleri L, Golijow CD. XRCC2 R188H (rs3218536), XRCC3 T241M (rs861539) and R243H (rs77381814) single nucleotide polymorphisms in cervical cancer risk. Pathol Oncol Res 2013;19(3):553-8.

Pina-Sanchez P, Hernandez-Hernandez DM, Lopez-Romero R, Vazquez-Ortiz G, Perez-Plasencia C, Lizano-Soberon M, et al. Human papillomavirus-specific viral types are common in Mexican women affected by cervical lesions. Int J Gynecol Cancer 2006;16(3):1041-7.

Pinto AP, Schlecht NF, Pintos J, Kaiano J, Franco EL, Crum CP, et al. Prognostic significance of lymph node variables and human papillomavirus DNA in invasive vulvar carcinoma. Gynecol Oncol 2004;92(3):856-65.

Pitta DR, Sarian LO, Campos EA, Rabelo-Santos SH, Syrjanen K, Derchain SF. Phylogenetic classification of human papillomavirus genotypes in high-grade cervical intraepithelial neoplasia in women from a densely populated Brazilian urban region. Sao Paulo Med J 2009;127(3):122-7.

Rabelo-Santos SH, Zeferino L, Villa LL, Sobrinho JP, Amaral RG, Magalhaes AV. Human papillomavirus prevalence among women with cervical intraepithelial neoplasia III and invasive cervical cancer from Goiania, Brazil. Mem Inst Oswaldo Cruz 2003;98(2):181-4.

Ragin CC, Wheeler VW, Wilson JB, Bunker CH, Gollin SM, Patrick AL, et al. Distinct distribution of HPV types among cancer-free Afro-Caribbean women from Tobago. Biomarkers 2007;12(5):510-22.

Ramas V, Mirazo S, Bonilla S, Mendoza L, Lago O, Basiletti J, et al. Human papillomavirus genotypes distribution in cervical samples from Uruguayan women. J Med Virol 2013;85(5):845-51.

Resende LS, Rabelo-Santos SH, Sarian LO, Alves RR, Ribeiro AA, Zeferino LC, et al. A portrait of single and multiple HPV type infections in Brazilian women of different age strata with squamous or glandular cervical lesions. BMC Infect Dis 2014;14(1):214.

Ribeiro AA, Figueiredo Alves RR, Costa MC, Villa LL, Zeferino LC, Mauricette Derchain SF, et al. Association between HPV types and species groups and cervical neoplasia from a high-risk area for cervical cancer, Goiania, Brazil. Int J Gynecol Pathol 2011;30(3):288-94.

Roa JC, Garcia P, Gomez J, Fernandez W, Gaete F, Espinoza A, et al. HPV genotyping from invasive cervical cancer in Chile. Int J Gynaecol Obstet 2009;105(2):150-3.

Rolon PA, Smith JS, Munoz N, Klug SJ, Herrero R, Bosch X, et al. Human papillomavirus infection and invasive cervical cancer in Paraguay. Int J Cancer 2000;85(4):486-91.

Rosa MI, Fachel JM, Rosa DD, Medeiros LR, Igansi CN, Bozzetti MC. Persistence and clearance of human papillomavirus infection: a prospective cohort study. Am J Obstet Gynecol 2008;199(6):617.

Roteli-Martins CM, De Carvalho NS, Naud P, Teixeira J, Borba P, Derchain S, et al. Prevalence of human papillomavirus infection and associated risk factors in young women in Brazil, Canada, and the United States: a multicenter cross-sectional study. Int J Gynecol Pathol 2011;30(2):173-84.

Safaeian M, Herrero R, Hildesheim A, Quint W, Freer E, Van Doorn LJ, et al. Comparison of the SPF10-LiPA system to the Hybrid Capture 2 Assay for detection of carcinogenic human papillomavirus genotypes among 5,683 young women in Guanacaste, Costa Rica. J Clin Microbiol 2007;45(5):1447-54.

Sanchez-Anguiano LF, Alvarado-Esquivel C, Reyes-Romero MA, Carrera-Rodriguez M. Human papillomavirus infections in women seeking cervical Papanicolaou cytology of Durango, Mexico: prevalence and genotypes. BMC Infect Dis 2006;6:27.

Sanchez-Lander J, Cortinas P, Loureiro CL, Pujol FH, Medina F, Capote-Negrin L, et al. Human papillomavirus in invasive cervical cancer and cervical intraepithelial neoplasia 2 and 3 in Venezuela: a cross-sectional study. Cancer Epidemiol 2012;36(5):e284-e287.

Santos C, Munoz N, Klug S, Almonte M, Guerrero I, Alvarez M, et al. HPV types and cofactors causing cervical cancer in Peru. Br J Cancer 2001;85(7):966-71.

Soto Y, Mune M, Morales E, Goicolea A, Mora J, Sanchez L, et al. Human papillomavirus infections in Cuban women with cervical intraepithelial neoplasia. Sex Transm Dis 2007;34(12):974-6.

Soto Y, Torres G, Kouri V, Limia CM, Goicolea A, Capo V, et al. Molecular epidemiology of human papillomavirus infections in cervical samples from Cuban women older than 30 years. J Low Genit Tract Dis 2014;18(3):210-7.

Soto-De Leon SC, Camargo M, Sanchez R, Leon S, Urquiza M, Acosta J, et al. Prevalence of infection with high-risk human papillomavirus in women in Colombia. Clinical Microbiology & Infection 2009;15(1):100-2.

Tabora N, Bakkers JM, Quint WG, Massuger LF, Matute JA, Melchers WJ, et al. Human papillomavirus infection in Honduran women with normal cytology. Cancer Causes Control 2009;20(9):1663-70.

Tabora N, Bulnes R, Toro LA, Claros JM, Massuger LF, Quint WG, et al. Human papillomavirus infection in Honduran women with cervical intraepithelial neoplasia or cervical cancer. J Low Genit Tract Dis 2011;15(1):48-53.

Tavares MC, de Macedo JL, de Lima Junior SF, de Andrade HS, Amorim MM, de Mascena Diniz MM, et al. Chlamydia trachomatis infection and human papillomavirus in women with cervical neoplasia in Pernambuco-Brazil. Mol Biol Rep 2014;41(2):865-74.

Trottier H, Mahmud S, Prado JC, Sobrinho JS, Costa MC, Rohan TE, et al. Type-specific duration of human papillomavirus infection: implications for human papillomavirus screening and vaccination. J Infect Dis 2008;197(10):1436-47.

Valles X, Murga GB, Hernandez G, Sabido M, Chuy A, Lloveras B, et al. High prevalence of human papillomavirus infection in the female population of Guatemala. Int J Cancer 2009;125(5):1161-7.

Walmer DK, Eder PS, Bell L, Salim H, Kobayashi L, Ndirangu J, et al. Human papillomavirus prevalence in a population of women living in Port-au-Prince and Leogane, Haiti. PLoS One 2013;8(10):e76110.

1.5 Northern America

Antonishyn NA, Horsman GB, Kelln RA, Saggar J, Severini A. The impact of the distribution of human papillomavirus types and associated high-risk lesions in a colposcopy population for monitoring vaccine efficacy. Arch Pathol Lab Med 2008;132(1):54-60.

Banister CE, Messersmith AR, Chakraborty H, Wang Y, Spiryda LB, Glover SH, et al. HPV prevalence at enrollment and baseline results from the Carolina Women's Care Study, a longitudinal study of HPV persistence in women of college age. Int J Womens Health 2013;5:379-88.

Barr E, Gause CK, Bautista OM, Railkar RA, Lupinacci LC, Insinga RP, et al. Impact of a prophylactic quadrivalent human papillomavirus (types 6, 11, 16, 18) L1 virus-like particle vaccine in a sexually active population of North American women. Am J Obstet Gynecol 2008;198(3):261-11.

Castle PE, Schiffman M, Wheeler CM, Wentzensen N, Gravitt PE. Human papillomavirus genotypes in cervical intraepithelial neoplasia grade 3. Cancer Epidemiol Biomarkers Prev 2010;19(7):1675-81.

Castle PE, Shaber R, Lamere B, Kinney W, Fetterman B, Poitras N, et al. Human papillomavirus (HPV) genotypes in women with cervical precancer and cancer at Kaiser Permanente Northern California. Cancer Epidemiol Biomarkers Prev 2011;20(5):946-53.

Chaturvedi AK, Dumestre J, Gaffga AM, Mire KM, Clark RA, Braly PS, et al. Prevalence of human papillomavirus genotypes in women from three clinical settings. J Med Virol 2005;75(1):105-13.

Cibas ES, Hong X, Crum CP, Feldman S. Age-specific detection of high risk HPV DNA in cytologically normal, computer-imaged ThinPrep Pap samples. Gynecol Oncol 2007;104(3):702-6.

Coutlee F, Ratnam S, Ramanakumar AV, Insinga RR, Bentley J, Escott N, et al. Distribution of human papillomavirus genotypes in cervical intraepithelial neoplasia and invasive cervical cancer in Canada. J Med Virol 2011;83(6):1034-41.

Crum CP, Beach KJ, Hedley ML, Yuan L, Lee KR, Wright TC, et al. Dynamics of human papillomavirus infection between biopsy and excision of cervical intraepithelial neoplasia: results from the ZYC101a protocol. J Infect Dis 2004;189(8):1348-54.

Daling JR, Madeleine MM, Schwartz SM, Shera KA, Carter JJ, McKnight B, et al. A population-based study of squamous cell vaginal cancer: HPV and cofactors. Gynecol Oncol 2002;84(2):263-70.

de Koning MN, Quint WG, Pirog EC. Prevalence of mucosal and cutaneous human papillomaviruses in different histologic subtypes of vulvar carcinoma. Mod Pathol 2008;21(3):334-44.

Demers AA, Shearer B, Severini A, Lotocki R, Kliewer EV, Stopera S, et al. Distribution of human papillomavirus types, cervical cancer screening history, and risk factors for infection in Manitoba. Chronic Dis Inj Can 2012;32(4):177-85.

Dunne EF, Unger ER, Sternberg M, McQuillan G, Swan DC, Patel SS, et al. Prevalence of HPV infection among females in the United States. JAMA 2007;297(8):813-9.

Evans MF, Adamson CS, Papillo JL, St John TL, Leiman G, Cooper K. Distribution of human papillomavirus types in ThinPrep Papanicolaou tests classified according to the Bethesda 2001 terminology and correlations with patient age and biopsy outcomes. Cancer 2006;106(5):1054-64.

Evans MF, Peng Z, Clark KM, Adamson CS, Ma XJ, Wu X, et al. HPV E6/E7 RNA in situ hybridization signal patterns as biomarkers of three-tier cervical intraepithelial neoplasia grade. PLoS One 2014;9(3):e91142.

Gaffga NH, Flagg EW, Weinstock HS, Shlay JC, Ghanem KG, Koutsky LA, et al. Monitoring HPV type-specific prevalence over time through clinic-based surveillance: a perspective on vaccine effectiveness. Vaccine 2012;30(11):1959-64.

Gargano JW, Nisenbaum R, Lee DR, Ruffin MT, Steinau M, Horowitz IR, et al. Age-group differences in human papillomavirus types and cofactors for cervical intraepithelial neoplasia 3 among women referred to colposcopy. Cancer Epidemiol Biomarkers Prev 2012;21(1):111-21.

Gargano JW, Wilkinson EJ, Unger ER, Steinau M, Watson M, Huang Y, et al. Prevalence of human papillomavirus types in invasive vulvar cancers and vulvar intraepithelial neoplasia 3 in the United States before vaccine introduction. J Low Genit Tract Dis 2012;16(4):471-9.

Giuliano AR, Papenfuss M, Abrahamsen M, Denman C, de Zapien JG, Henze JL, et al. Human papillomavirus infection at the United States-Mexico border: implications for cervical cancer prevention and control. Cancer Epidemiol Biomarkers Prev 2001;10(11):1129-36.

Giuliano AR, Harris R, Sedjo RL, Baldwin S, Roe D, Papenfuss MR, et al. Incidence, prevalence, and clearance of type-specific human papillomavirus infections: The Young Women's Health Study. J Infect Dis 2002;186(4):462-9.

Goodman MT, Shvetsov YB, McDuffie K, Wilkens LR, Zhu X, Thompson PJ, et al. Prevalence, acquisition, and clearance of cervical human papillomavirus infection among women with normal cytology: Hawaii Human Papillomavirus Cohort Study. Cancer Res 2008;68(21):8813-24.

Guo M, Sneige N, Silva EG, Jan YJ, Cogdell DE, Lin E, et al. Distribution and viral load of eight oncogenic types of human papillomavirus (HPV) and HPV 16 integration status in cervical intraepithelial neoplasia and carcinoma. Mod Pathol 2007;20(2):256-66.

Hamlin-Douglas LK, Coutlee F, Roger M, Franco EL, Brassard P. Prevalence and age distribution of human papillomavirus infection in a population of Inuit women in Nunavik, Quebec. Cancer Epidemiol Biomarkers Prev 2008;17(11):3141-9.

Hariri S, Unger ER, Sternberg M, Dunne EF, Swan D, Patel S, et al. Prevalence of genital human papillomavirus among females in the United States, the National Health And Nutrition Examination Survey, 2003-2006. J Infect Dis 2011;204(4):566-73.

Hariri S, Steinau M, Rinas A, Gargano JW, Ludema C, Unger ER, et al. HPV genotypes in high grade cervical lesions and invasive cervical carcinoma as detected by two commercial DNA assays, North Carolina, 2001-2006. PLoS ONE 2012;7(3):e34044.

Hariri S, Unger ER, Powell SE, Bauer HM, Bennett NM, Bloch KC, et al. Human papillomavirus genotypes in high-grade cervical lesions in the United States. J Infect Dis 2012;206(12):1878-86.

Hopenhayn C, Christian A, Christian WJ, Watson M, Unger ER, Lynch CF, et al. Prevalence of human papillomavirus types in invasive cervical cancers from 7 US cancer registries before vaccine introduction. J Low Genit Tract Dis 2014;18(2):182-9.

Hu L, Guo M, He Z, Thornton J, McDaniel LS, Hughson MD. Human papillomavirus genotyping and p16INK4a expression in cervical intraepithelial neoplasia of adolescents. Mod Pathol 2005;18(2):267-73.

Insinga RP, Liaw KL, Johnson LG, Madeleine MM. A systematic review of the prevalence and attribution of human papillomavirus types among cervical, vaginal, and vulvar precancers and cancers in the United States. Cancer Epidemiol Biomarkers Prev 2008;17(7):1611-22.

Jiang Y, Brassard P, Severini A, Goleski V, Santos M, Leamon A, et al. Type-specific prevalence of Human Papillomavirus infection among women in the Northwest Territories, Canada. J Infect Public Health 2011;4(5-6):219-27.

Jiang Y, Brassard P, Severini A, Mao Y, Li YA, Laroche J, et al. The prevalence of human papillomavirus and its impact on cervical dysplasia in Northern Canada. Infect Agent Cancer 2013;8(1):25.

Kelly JJ, Unger ER, Dunne EF, Murphy NJ, Tiesinga J, Koller KR, et al. HPV genotypes detected in cervical cancers from Alaska Native women, 1980-2007. Int J Circumpolar Health 2013;72:21115.

Koushik A, Ghosh A, Duarte-Franco E, Forest P, Voyer H, Matlashewski G, et al. The p53 codon 72 polymorphism and risk of high-grade cervical intraepithelial neoplasia. Cancer Detect Prev 2005;29(4):307-16.

Kulasingam SL, Hughes JP, Kiviat NB, Mao C, Weiss NS, Kuypers JM, et al. Evaluation of human papillomavirus testing in primary screening for cervical abnormalities: comparison of sensitivity, specificity, and frequency of referral. JAMA 2002;288(14):1749-57.

Lee SH, Vigliotti VS, Pappu S. HPV infection among women in a representative rural and suburban population of the USA. Int J Gynaecol Obstet 2009;105(3):210-4.

Likes W, Bloom L. Human papillomavirus distribution in vulvar intraepithelial neoplasia. Appl Nurs Res 2012;25(4):280-2.

Logani S, Lu D, Quint WG, Ellenson LH, Pirog EC. Low-grade vulvar and vaginal intraepithelial neoplasia: correlation of histologic features with human papillomavirus DNA detection and MIB-1 immunostaining. Mod Pathol 2003;16(8):735-41.

Malik ZA, Hailpern SM, Burk RD. Persistent antibodies to HPV virus-like particles following natural infection are protective against subsequent cervicovaginal infection with related and unrelated HPV. Viral Immunol 2009;22(6):445-9.

Markowitz LE, Hariri S, Lin C, Dunne EF, Steinau M, McQuillan G, et al. Reduction in human papillomavirus (HPV) prevalence among young women following HPV vaccine introduction in the United States, National Health and Nutrition Examination Surveys, 2003-2010. J Infect Dis 2013;208(3):385-93.

Moore RA, Ogilvie G, Fornika D, Moravan V, Brisson M, Amirabbasi-Beik M, et al. Prevalence and type distribution of human papillomavirus in 5,000 British Columbia women-implications for vaccination. Cancer Causes Control 2009;20:1387-96.

Moscicki AB, Ellenberg JH, Farhat S, Xu J. Persistence of human papillomavirus infection in HIV-infected and -uninfected adolescent girls: risk factors and differences, by phylogenetic type. J Infect Dis 2004;190(1):37-45.

Namugenyi SB, Balsan MJ, Glick SN, Jordan JA. Prevalence and genotype distribution of human papillomavirus in cytology specimens containing atypical glandular cells: a case-control study. J Clin Virol 2013;58(2):432-6.

Ogilvie GS, Cook DA, Taylor DL, Rank C, Kan L, Yu A, et al. Population-based evaluation of type-specific HPV prevalence among women in British Columbia, Canada. Vaccine 2013;31(7):1129-33.

Peyton CL, Gravitt PE, Hunt WC, Hundley RS, Zhao M, Apple RJ, et al. Determinants of genital human papillomavirus detection in a US population. J Infect Dis 2001;183(11):1554-64.

Pirog EC, Kleter B, Olgac S, Bobkiewicz P, Lindeman J, Quint WG, et al. Prevalence of human papillomavirus DNA in different histological subtypes of cervical adenocarcinoma. Am J Pathol 2000;157(4):1055-62.

Quint KD, de Koning MN, Geraets DT, Quint WG, Pirog EC. Comprehensive analysis of Human Papillomavirus and Chlamydia trachomatis in in-situ and invasive cervical adenocarcinoma. Gynecol Oncol 2009;114(3):390-4.

Quint KD, de Koning MN, Van Doorn LJ, Quint WG, Pirog EC. HPV genotyping and HPV16 variant analysis in glandular and squamous neoplastic lesions of the uterine cervix. Gynecol Oncol 2010;117(2):297-301.

Ralston Howe E, Li Z, McGlennen RC, Hellerstedt WL, Downs LS, Jr. Type-specific prevalence and persistence of human papillomavirus in women in the United States who are referred for typing as a component of cervical cancer screening. Am J Obstet Gynecol 2009;200(3):245-7.

Reiter PL, Katz ML, Ruffin MT, Hade EM, Degraffenreid CR, Patel DA, et al. HPV prevalence among women from Appalachia: results from the CARE project. PLoS One 2013;8(8):e74276.

Richardson H, Kelsall G, Tellier P, Voyer H, Abrahamowicz M, Ferenczy A, et al. The natural history of type-specific human papillomavirus infections in female university students. Cancer Epidemiol Biomarkers Prev 2003;12(6):485-90.

Richardson H, Franco E, Pintos J, Bergeron J, Arella M, Tellier P. Determinants of low-risk and high-risk cervical human papillomavirus infections in Montreal University students. Sex Transm Dis 2000;27(2):79-86.

Roteli-Martins CM, De Carvalho NS, Naud P, Teixeira J, Borba P, Derchain S, et al. Prevalence of human papillomavirus infection and associated risk factors in young women in Brazil, Canada, and the United States: a multicenter cross-sectional study. Int J Gynecol Pathol 2011;30(2):173-84.

Saraiya M, Benard VB, Greek AA, Steinau M, Patel S, Massad LS, et al. Type-specific HPV and Pap test results among low income, underserved women: providing iInsights into management strategies. Am J Obstet Gynecol 2014;211(4):354.e1-354.e6.

Schiffman M, Glass AG, Wentzensen N, Rush BB, Castle PE, Scott DR, et al. A long-term prospective study of type-specific human papillomavirus infection and risk of cervical neoplasia among 20,000 women in the Portland Kaiser Cohort Study. Cancer Epidemiol Biomarkers Prev 2011;20(7):1398-409.

Schwartz SM, Daling JR, Shera KA, Madeleine MM, McKnight B, Galloway DA, et al. Human papillomavirus and prognosis of invasive cervical cancer: a population-based study. J Clin Oncol 2001;19(7):1906-15.

Severini A, Jiang Y, Brassard P, Morrison H, Demers AA, Oguntuase E, et al. Type-specific prevalence of human papillomavirus in women screened for cervical cancer in Labrador, Canada. Int J Circumpolar Health 2013;72:19743.

Shikary T, Bernstein DI, Jin Y, Zimet GD, Rosenthal SL, Kahn JA. Epidemiology and risk factors for human papillomavirus infection in a diverse sample of low-income young women. J Clin Virol 2009;46(2):107-11.

Sinno AK, Saraiya M, Thompson TD, Hernandez BY, Goodman MT, Steinau M, et al. Human papillomavirus genotype prevalence in invasive vaginal cancer from a registry-based population. Obstet Gynecol 2014;123(4):817-21.

Srodon M, Stoler MH, Baber GB, Kurman RJ. The distribution of low and high-risk HPV types in vulvar and vaginal intraepithelial neoplasia (VIN and VaIN). Am J Surg Pathol 2006;30(12):1513-8.

Sutton BC, Allen RA, Moore WE, Dunn ST. Distribution of human papillomavirus genotypes in invasive squamous carcinoma of the vulva. Mod Pathol 2008;21(3):345-54.

Tarkowski TA, Koumans EH, Sawyer M, Pierce A, Black CM, Papp JR, et al. Epidemiology of human papillomavirus infection and abnormal cytologic test results in an urban adolescent population. J Infect Dis 2004;189(1):46-50.

Thomas KK, Hughes JP, Kuypers JM, Kiviat NB, Lee SK, Adam DE, et al. Concurrent and sequential acquisition of different genital human papillomavirus types. J Infect Dis 2000;182(4):1097-102.

Tricco AC, Ng CH, Gilca V, Anonychuk A, Pham B, Berliner S. Canadian oncogenic human papillomavirus cervical infection prevalence: systematic review and meta-analysis. BMC Infect Dis 2011;11:235.

Wentzensen N, Schiffman M, Dunn T, Zuna RE, Gold MA, Allen RA, et al. Multiple human papillomavirus genotype infections in cervical cancer progression in the study to understand cervical cancer early endpoints and determinants. Int J Cancer 2009;125(9):2151-8.

Wentzensen N, Wilson LE, Wheeler CM, Carreon JD, Gravitt PE, Schiffman M, et al. Hierarchical clustering of human papilloma virus genotype patterns in the ASCUS-LSIL triage study. Cancer Res 2010;70(21):8578-86.

Wheeler CM, Hunt WC, Joste NE, Key CR, Quint WG, Castle PE. Human papillomavirus genotype distributions: implications for vaccination and cancer screening in the United States. J Natl Cancer Inst 2009;101(7):475-87.

Wheeler CM, Hunt WC, Cuzick J, Langsfeld E, Pearse A, Montoya GD, et al. A population-based study of human papillomavirus genotype prevalence in the United States: baseline measures prior to mass human papillomavirus vaccination. Int J Cancer 2013;132(1):198-207.

Widdice LE, Brown DR, Bernstein DI, Ding L, Patel D, Shew M, et al. Prevalence of human papillomavirus infection in young women receiving the first quadrivalent vaccine dose. Arch Pediatr Adolesc Med 2012;166(8):774-6.

Winer RL, Lee SK, Hughes JP, Adam DE, Kiviat NB, Koutsky LA. Genital human papillomavirus infection: incidence and risk factors in a cohort of female university students. Am J Epidemiol 2003;157(3):218-26.

Winer RL, Hughes JP, Feng Q, O'Reilly S, Kiviat NB, Holmes KK, et al. Condom use and the risk of genital human papillomavirus infection in young women. N Engl J Med 2006;354(25):2645-54.

Wright AA, Howitt BE, Myers AP, Dahlberg SE, Palescandolo E, Van HP, et al. Oncogenic mutations in cervical cancer: genomic differences between adenocarcinomas and squamous cell carcinomas of the cervix. Cancer 2013;119(21):3776-83.

Wright JD, Li J, Gerhard DS, Zhang Z, Huettner PC, Powell MA, et al. Human papillomavirus type and tobacco use as predictors of survival in early stage cervical carcinoma. Gynecol Oncol 2005;98(1):84-91.

Wright TC, Jr., Stoler MH, Sharma A, Zhang G, Behrens C, Wright TL. Evaluation of HPV-16 and HPV-18 genotyping for the triage of women with high-risk HPV+ cytology-negative results. Am J Clin Pathol 2011;136(4):578-86.

Zuna RE, Allen RA, Moore WE, Lu Y, Mattu R, Dunn ST. Distribution of HPV genotypes in 282 women with cervical lesions: evidence for three categories of intraepithelial lesions based on morphology and HPV type. Mod Pathol 2007;20(2):167-74.

1.6 World

Alemany L, de SS, Tous S, Quint W, Vallejos C, Shin HR, et al. Time trends of human papillomavirus types in invasive cervical cancer, from 1940 to 2007. Int J Cancer 2014;135(1):88-95.

Bernard E, Pons-Salort M, Favre M, Heard I, Delarocque-Astagneau E, Guillemot D, et al. Comparing human papillomavirus prevalences in women with normal cytology or invasive cervical cancer to rank genotypes according to their oncogenic potential: a meta-analysis of observational studies. BMC Infect Dis 2013;13(1):373.

Bosch FX, Burchell AN, Schiffman M, Giuliano AR, de Sanjose S, Bruni L, et al. Epidemiology and natural history of human papillomavirus infections and type-specific implications in cervical neoplasia. Vaccine 2008;26 Suppl 10:K1-16.

Bruni L, Diaz M, Castellsague X, Ferrer E, Bosch FX, de Sanjose S. Cervical human papillomavirus prevalence in 5 continents: meta-analysis of 1 million women with normal cytological findings. J Infect Dis 2010;202(12):1789-99.

Bzhalava D, Guan P, Franceschi S, Dillner J, Clifford G. A systematic review of the prevalence of mucosal and cutaneous human papillomavirus types. Virology 2013;445(1-2):224-31.

Castellsague X, Diaz M, de Sanjose S, Munoz N, Herrero R, Franceschi S, et al. Worldwide human papillomavirus etiology of cervical adenocarcinoma and its cofactors: implications for screening and prevention. J Natl Cancer Inst 2006;98(5):303-15.

Clifford GM, Gallus S, Herrero R, Munoz N, Snijders PJ, Vaccarella S, et al. Worldwide distribution of human papillomavirus types in cytologically normal women in the International Agency for Research on Cancer HPV prevalence surveys: a pooled analysis. Lancet 2005;366(9490):991-8.

de Sanjose S, Diaz M, Castellsague X, Clifford G, Bruni L, Munoz N, et al. Worldwide prevalence and genotype distribution of cervical human papillomavirus DNA in women with normal cytology: a meta-analysis. Lancet Infect Dis 2007;7(7):453-9.

de Sanjose S, Quint WG, Alemany L, Geraets DT, Klaustermeier JE, Lloveras B, et al. Human papillomavirus genotype attribution in invasive cervical cancer: a retrospective cross-sectional worldwide study. Lancet Oncol 2010;11(11):1048-56.

de Sanjose S, Alemany L, Ordi J, Tous S, Alejo M. Worldwide human papillomavirus genotype attribution in over 2000 cases of intraepithelial and invasive lesions of the vulva. Eur J Cancer 2013;49(16):3450-61.

De Vuyst H, Clifford GM, Nascimento MC, Madeleine MM, Franceschi S. Prevalence and type distribution of human papillomavirus in carcinoma and intraepithelial neoplasia of the vulva, vagina and anus: a meta-analysis. Int J Cancer 2009;124(7):1626-36.

Garland SM, Insinga RP, Sings HL, Haupt RM, Joura EA. Human papillomavirus infections and vulvar disease development. Cancer Epidemiol Biomarkers Prev 2009;18(6):1777-84.

Guan P, Howell-Jones R, Li N, Bruni L, de SS, Franceschi S, et al. Human papillomavirus types in 115,789 HPV-positive women: A meta-analysis from cervical infection to cancer. Int J Cancer 2012;131(10):2349-59.

Li N, Franceschi S, Howell-Jones R, Snijders PJ, Clifford GM. Human papillomavirus type distribution in 30,848 invasive cervical cancers worldwide: Variation by geographical region, histological type and year of publication. Int J Cancer 2011;128(4):927-35.

Liu P, Xu L, Sun Y, Wang Z. The prevalence and risk of human papillomavirus infection in pregnant women. Epidemiol Infect 2014;142(8):1567-78.

Munoz N, Bosch FX, Castellsague X, Diaz M, de Sanjose S, Hammouda D, et al. Against which human papillomavirus types shall we vaccinate and screen? The international perspective. Int J Cancer 2004;111(2):278-85.

Pirog EC, Lloveras B, Molijn A, Tous S, Guimera N, Alejo M, et al. HPV prevalence and genotypes in different histological subtypes of cervical adenocarcinoma, a worldwide analysis of 760 cases. Mod Pathol 2014;27(12):1559-67.

Smith JS, Lindsay L, Hoots B, Keys J, Franceschi S, Winer R, et al. Human papillomavirus type distribution in invasive cervical cancer and high-grade cervical lesions: a meta-analysis update. Int J Cancer 2007;121(3):621-32.

Smith JS, Backes DM, Hoots BE, Kurman RJ, Pimenta JM. Human papillomavirus type-distribution in vulvar and vaginal cancers and their associated precursors. Obstet Gynecol 2009;113(4):917-24.
